# Dataset of the aqueous solution and petrochemical wastewater treatment containing ammonia using low cost and efficient bio-adsorbents

**DOI:** 10.1016/j.dib.2019.104308

**Published:** 2019-08-22

**Authors:** Golan Yeganeh, Bahman Ramavandi, Hossein Esmaeili, Sajad Tamjidi

**Affiliations:** a Young Researchers and Elite Club, Bushehr Branch, Islamic Azad University, Bushehr, Iran; b Department of Environmental Health Engineering, Faculty of Health and Nutrition, Bushehr University of Medical Sciences, Bushehr, Iran; c Systems Environmental Health and Energy Research Center, The Persian Gulf Biomedical Sciences Research Institute, Bushehr University of Medical Sciences, Bushehr, Iran; d Department of Chemical Engineering, Bushehr Branch, Islamic Azad University, Bushehr, Iran

**Keywords:** Adsorption, *Sargassum oligocystum*, *Ziziphus spina-Christi*, Ammonia, Petrochemical wastewater

## Abstract

In this dataset, the removal of ammonia from synthetic and real wastewater was studied using the *Ziziphus spina-christi* activated carbon (ZSAC) and the biochar of *Sargassum oligocystum* (BSO). Several analyses such as FTIR, SEM, EDS, XRD, and BET were used to determine the physical and surface properties of the adsorbents. The BET analysis showed a high specific surface area of 112.5 and 45.8 m^2^/g for ZSAC and BSO, respectively. Also, the results indicated that the highest adsorption of ammonia from synthetic wastewater using ZSAC and BSO were obtained 97.9% and 96.2%, at contact time of 80 min, 25 °C, pH 8, and adsorbent dosage of 5 g/L. In addition, the adsorption results of real wastewater from Asaluyeh Pardis Petrochemical Company demonstrated that both adsorbents had the removal efficiency of approximately 90%, which indicates high adsorption efficiency using two adsorbents. Moreover, equilibrium studies showed that the adsorption process of ammonia from wastewater using both adsorbents follows the Freundlich model and the maximum adsorption capacity using the Langmuir isotherm were calculated to be 25.77 mg/g and 7.46 mg/g for ZSAC and BSO, respectively. Furthermore, the thermodynamic study showed that the adsorption process using the bio-adsorbents was spontaneous and exothermic.

**Table:** Specifications Table

Subject area	Chemical Engineering
More specific subject area	Environmental Chemical Engineering
Type of data	Table, image, figure
How data was acquired	– SEM analysis (VEGA III, TESCAN; Czech Republic) was used to determine the morphology and identify the structure of bio-adsorbents before and after adsorption. – FTIR analysis (Avatar, THERMO, USA) was applied to specify the functional groups. – BET analysis (Micromeritics, ASAP 2020, USA) was used to measure the surface area of the adsorbent. – XRD analysis (Bruker AXS-D8, pw1730, Germany) was applied to determine the crystalline structure and phases on the adsorbent surface. – EDS test (VEGA, TESCAN; Czech Republic) was used to determine the percentage of the elements in the adsorbents. – A flame atomic absorption spectroscopy (GBC, Avanta) with acetylene-air fuel was used to determine the amount of ammonia ion in the wastewater.
Data format	Analyzed
Experimental factors	– The effect of bio-adsorbent dosage, temperature, pH and contact time was acquired. – The isotherm and thermodynamic parameters were revealed. – A petrochemical wastewater containing ammonia was treated using the bio-adsorbents.
Experimental features	Ammonia ions adsorption by ZSAC and BSO
Data source location	Islamic Azad University of Bushehr, Iran
Data accessibility	Data represented with the article

**Table:** 

**Value of the data** • For the first time, two efficient bio-adsorbents namely *Z. spina-christi* activated carbon and the brown alga of *S. oligocystum* were used for treatment of ammonia-laden petrochemical wastewater. • This data can be useful for managers of wastewater treatment plants or petrochemical companies facing ammonia problem in wastewater. • The used bio-adsorbents could be beneficial as fertilizer in the agricultural lands.

## 1 Data

**Fig. 1:**
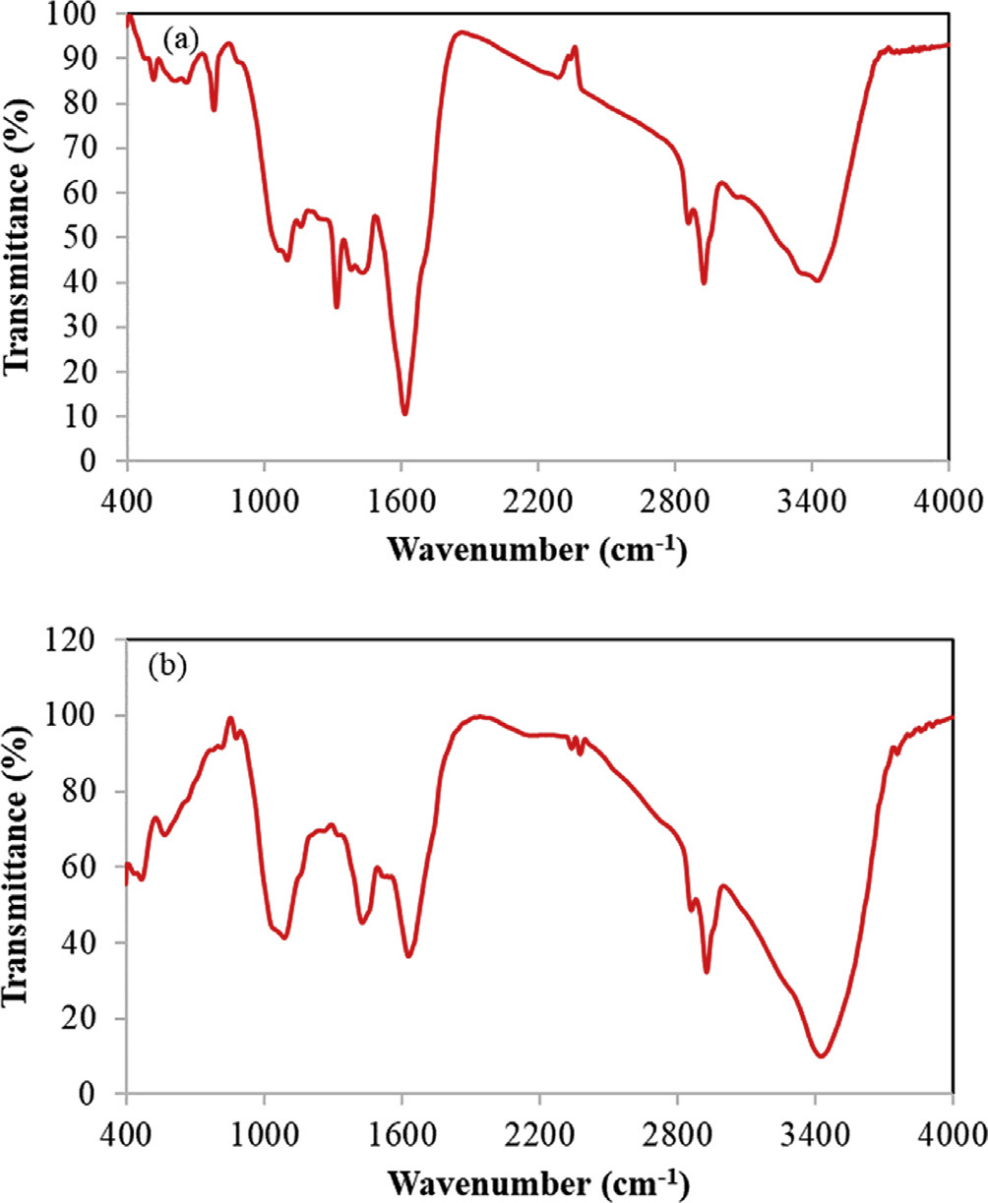
The FTIR analysis for **(a)** ZSAC and **(b)** BSO.

**Fig. 2:**
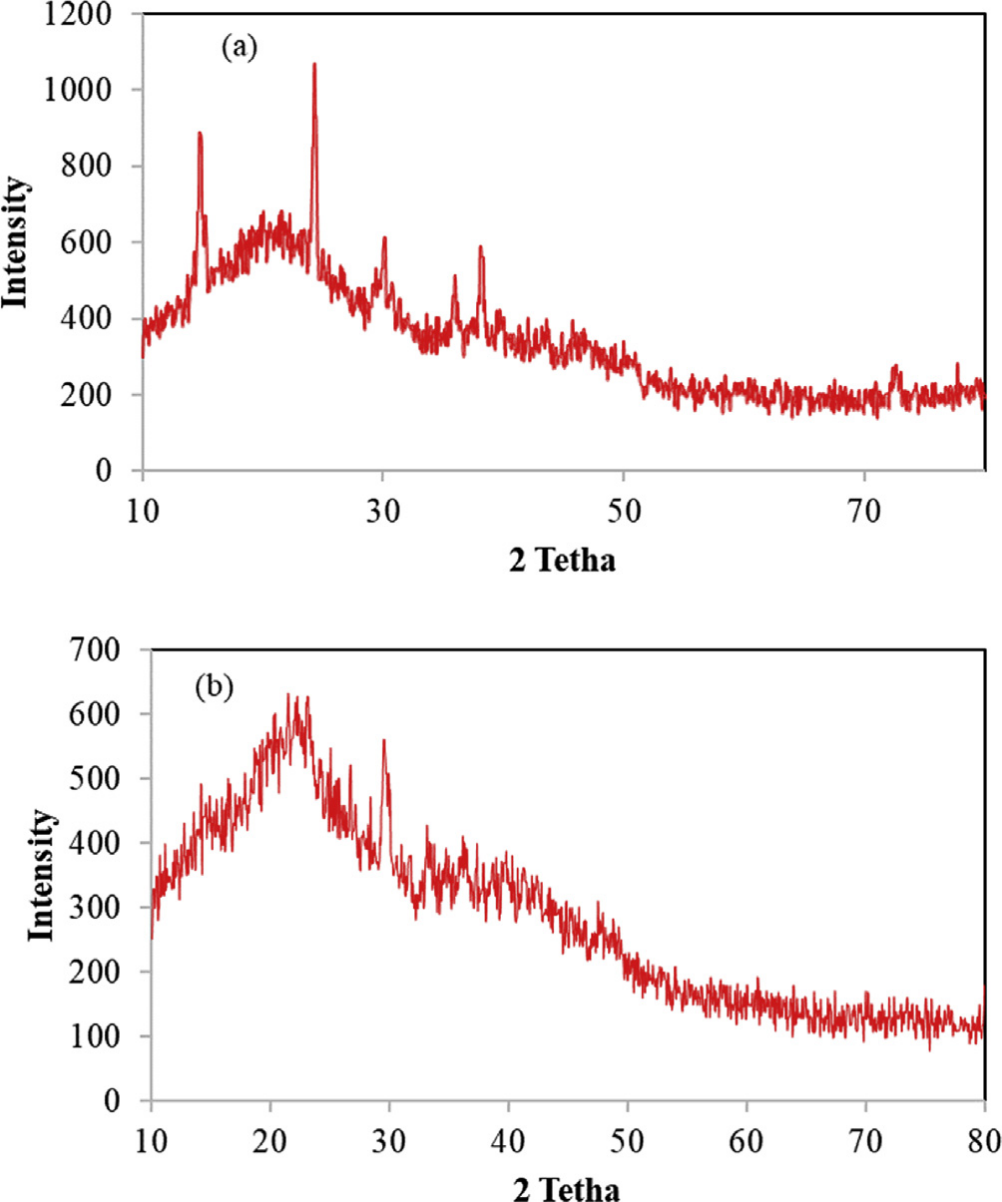
XRD analysis for **(a)** ZSAC and **(b)** BSO.

**Fig. 3:**
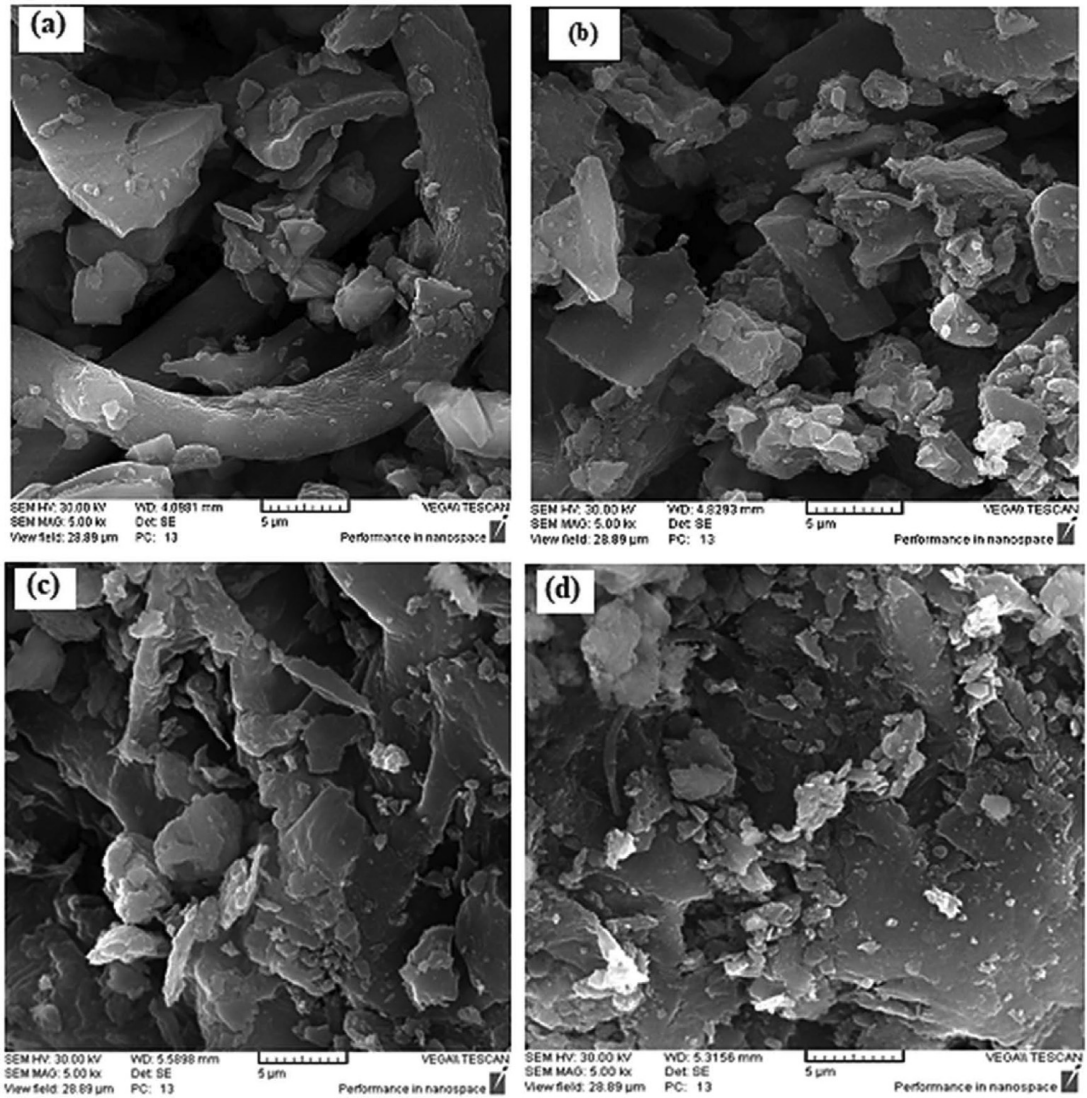
SEM images of (a) ZSAC before adsorption, (b) ZSAC after adsorption, (c) BSO before adsorption, and (d) BSO after adsorption.

**Fig. 4:**
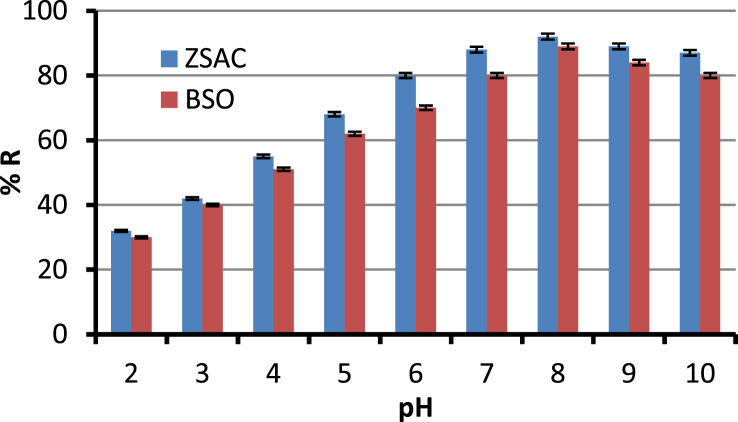
Effect of pH on the ammonium ion adsorption (contact time: 60 min, ammonium ion concentration: 100 mg/L, temperature: 25 °C, bio-adsorbent dose: 2 g/L, and mixture rate: 200 rpm).

**Fig. 5:**
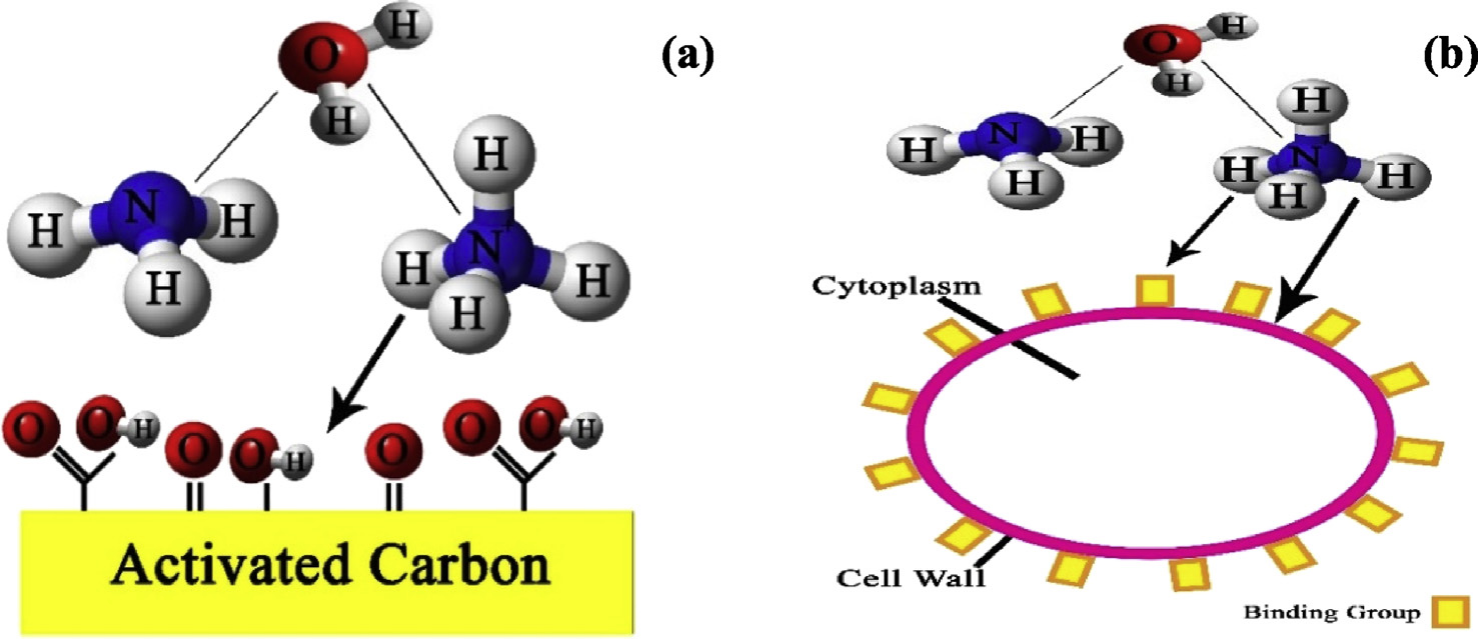
Schematic for the adsorption mechanism of ammonium ion onto **(a)** ZSAC and **(b)** BSO.

**Fig. 6:**
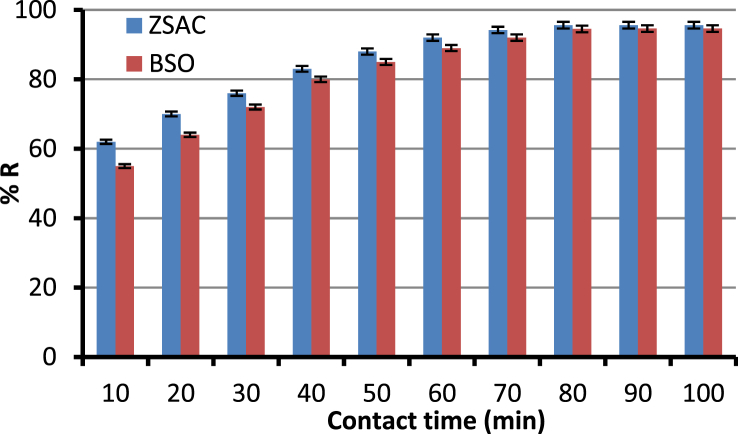
Effect of contact time on the ammonium ion adsorption (pH: 8, temperature: 25 °C, bio-adsorbent dose: 2 g/L, ammonium ion concentration: 100 mg/L, mixture rate: 200 rpm).

**Fig. 7:**
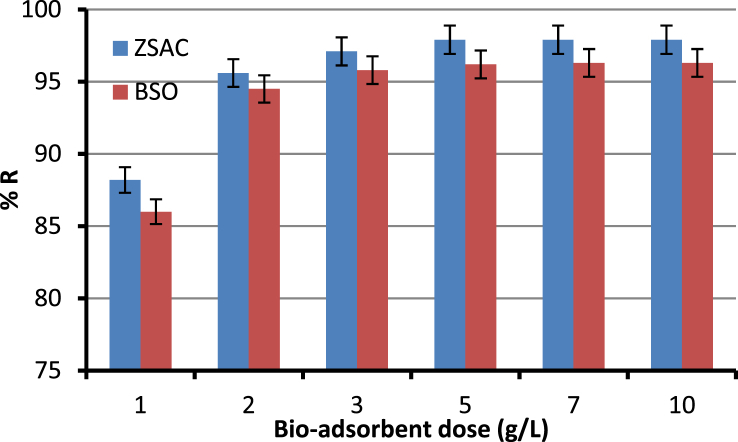
Effect of bio-adsorbent dose on the ammonium ion adsorption (pH: 8, contact time: 80 min, temperature: 25 °C, ammonium ion concentration: 100 mg/L, mixture rate: 200 rpm).

**Fig. 8:**
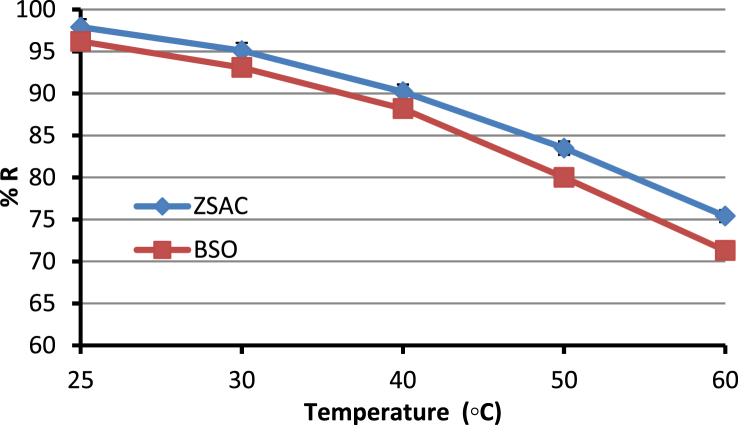
Effect of temperatures on the ammonium ion adsorption (pH: 8, contact time: 80 min, bio-adsorbent dose: 5 g/L, ammonium ion concentration: 100 mg/L, mixture rate: 200 rpm m).

**Fig. 9:**
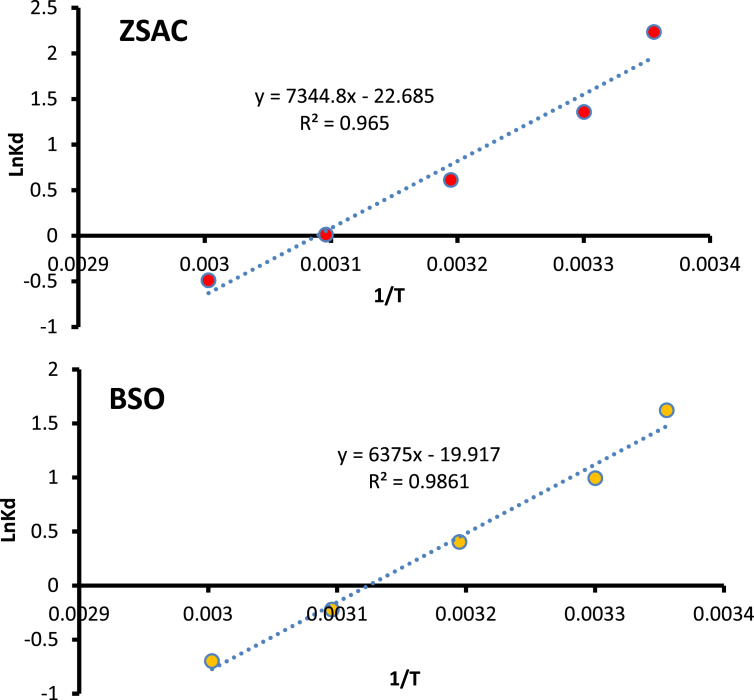
LnK_d_ against 1/T for calculation thermodynamic parameters.

The current dataset contains 9 figures and 6 tables. BET data for bio-adsorbents are given in Table 1. The FT-IR spectra of ZSAC and BSO at wavenumbers range of 400–4000 cm^−1^ are shown in Fig. 1. The results of the EDS elemental test for both bio-adsorbents are given in Table 2. The XRD spectrum of the bio-adsorbents is shown in Fig. 2. Fig. 3a and b shows SEM images of ZSAC and BSO before and after ammonium ion adsorption process. Fig. 4 shows the effect of pH on the ammonium ions adsorption by ZSAC and BSO bio-adsorbents. Fig. 5 is a schematic which depicts the adsorption mechanism of the ammonium ions onto the bio-adsorbents. Fig. 6 shows the ammonium ion removal over different contact times. Fig. 7 shows the effect of bio-adsorbent dosage on the removal percentage of ammonium ion from aqueous medium. The effect of different temperatures on the ammonium ion adsorption using ZSAC and BSO has been shown in Fig. 8. The results of Langmuir and Freundlich isotherms are given in Table 3. The maximum adsorption capacity of ammonium ion using ZSAC and BSO bio-adsorbents in other studies is shown in Table 4. LnKd against 1/T was used for calculating thermodynamic parameters (Fig. 9). The results of the thermodynamic calculations are shown in Table 5. A petrochemical wastewater was treated using ZSAC and BSO and the results are listed in Table 6.

**Table 1:** Surface characteristics of ZSAC and BSO bio-adsorbents using BET analysis.

Characteristic	Unit	ZSAC	BSO
aBET surface area	m^2^/g	112.50	45.80
Total pore volume (p/p_0_ = 0.99)	cm^3^/g	0.905	0.075
Mean pore diameter	nm	15.30	18.20

a The BET surface area of these bio-adsorbents is higher than activated carbon prepared from worn tires (32.39 m^2^/g) [1] and bamboo (2.80 m^2^/g) [2] and less than commercial activated carbon (717.22 m^2^/g) [3] and activated carbon prepared from coconut shell (370.72 m^2^/g) [3].

**Table 2:** The EDS test for determination of the elemental percentage in the adsorbents.

Elements	ZSAC		BSO	
	Weight percent (%)	Atomic percent (%)	Weight percent (%)	Weight percent (%)
C	57.99	67.39	34.50	34.50
O	33.71	29.40	51.20	51.20
Mg	0.60	0.34	1.45	1.45
Al	0.24	0.13	1.28	1.28
Si	0.46	0.23	4.64	4.64
Cl	1.02	0.40	–	–
K	3.35	1.19	0.89	0.89
Ca	2.63	0.92	3.77	3.77
Fe	–	–	1.45	1.45
S	–	–	0.82	0.82
	100.00	100.00	100.00	100.00

**Table 3:** Constants and parameters of Langmuir and Freundlich isotherms for ammonium ion adsorption using ZSAC and BSO.

Isotherm	Parameters	ZSAC	BSO
Langmuir	q_max_ (mg/g)	25.77	7.46
	b (L/mg)	0.09	0.49
	R^2^	0.991	0.987
Freundlich	K_f_	7.23	4.58
	n	2.33	1.72
	R^2^	0.998	0.991

**Table 4:** Maximum adsorption capacity of ammonium ion using different bio-adsorbents.

Bio-adsorbent	q_max_ (mg/g)	Reference
Activated carbon	17.19	[4]
*Posidonia oceanica* fibers	1.73	[5]
Light expanded clay aggregate (LECA)	0.255	[6]
Multi-walled carbon nanotubes	9.31	[7]
Pin sawdust and wheat straw biochars	5.38	[8]
Volcanic tuff	19	[9]
Organic acid modified activated carbon and activated carbon	19.34 and 4.50	[10]
ZSAC	25.77	Present work
BSO	7.46	Present work

**Table 5:** Thermodynamic study of ammonium ion adsorption using ZSAC and BSO.

Bio-adsorbent	Temperature (K)	ΔG^o^ (kJ/mol)	ΔH^o^ (kJ/mol)	ΔS^o^ (J/mol.K)
ZSAC	298	−5.53	−61.07	−0.188
	303	−3.42		
	313	−1.59		
	323	−0.032		
	333	1.36		
BSO	298	−12.54	−53.01	−0.166
	303	−6.79		
	313	−3.89		
	323	−2.15		
	333	−1.37

**Table 6:** Treatment of wastewater sampled from Pardis Petrochemical Company (Asaluyeh, Iran) using ZSAC and BSO (pH: as per original, temperature: 25 °C, bio-adsorbent dose: 5 g/L, mixture rate: 200 rpm).

Wastewater quality	Sample 1: Raw sewage laver			Sample 2: laver TK-5601		
	Raw concentration	Treated by		Raw concentration	Treated by	
		ZSAC	BSO		ZSAC	BSO
a NH_4_^+^ (mg/L)	300	33	39	45	4	4.5
BOD_5_ (mg/L)	284	273	276	185	173.4	176
COD (mg/L)	565	554	555	364	355	353
pH	8.9	8.5	8.2	8.8	8.4	8.5
Electrical conductivity (μS/cm)	1600	1510	1515	350	300	305

a Note:According to Iranian EPA, the ammonia content of the treated wastewater did not meet the standard value (2.5 mg/L) to be discharged into water bodies. Thus, the ammonia pollutant of the wastewater should be further treated.

## 2 Experimental design, materials, and methods

### 2.1 Materials

Ammonium chloride, hydrochloric acid, and sodium hydroxide were purchased from Merck Company (Germany). The double distilled water has been used to prepare solutions. In order to prepare 1 L of ammonia stock solution by using ammonium chloride, 2.96 g of ammonium chloride was added to 1000 mL of double distilled water.

### 2.2 Bio-adsorbents preparation method

*Ziziphus spina-christi* leaves were initially washed with distilled water to remove dust and other pollutants. Then the leaves were placed in an oven for 1 h at a temperature of 80 °C to be dried. After that, dried leaves were heated about 1 h on the gentle flame to be ash. Next, the ashes were transferred into the furnace at 700 °C for 3 h to be carbonized. The carbonized material was powdered by the mill, graded by sieve No. 25 and used as ZSAC bio-adsorbent in the ammonia adsorption.

After gathering the *S. oligocystum* brown alga from the Persian Gulf, they were washed several times with distilled water to remove waste and dirty. Then, they were placed inside the oven at 105 °C for 24 h to be dried. Next, the dried (biochar) algae powdered by the mill and graded by sieve No. 25 and used as BSO bio-adsorbent in the ammonia adsorption.

### 2.3 Adsorption experiments

In this dataset, the removal of ammonia ion from aqueous solution using two bio-adsorbents has been investigated. To do this, the effect of various parameters such as pH (2, 3, 4, 5, 6, 7, 8, 9, 10), bio-adsorbent dose (1, 2, 3, 5, 7, 10 g/L), contact time (10, 20, 30, 40, 50, 60, 70, 80 min), and temperature (25, 30, 40, 50, 60 °C) at the conditions of mixing rate of 200 rpm and the initial ammonia concentration of 100 mg/l on the adsorption efficiency was examined. Finally, the reaction solution was filtered and an atomic adsorption device was used to measure the ammonia ion in the solution. The percentage of removed ammonia (%R) and adsorption capacity ($q_{e,}$mg/g) were determined by the following formulas:

(1) $$\text{\%R}=\frac{\left(C_{i}-{\text{C}}_{o}\right)}{{\text{C}}_{i}}\;\times 100$$

(2) $$q_{e=}\left(C_{i}-C_{0}\right)\frac{v}{w}$$

where $C_{i}$ and $C_{0}$ (mg/L) are the initial and equilibrium concentration of ammonia ions, respectively. V (L) is solution volume and w (g) indicates the applied bio-adsorbent mass.

All experiments were done three times and the mean and standard deviation (as error bars) of the analysis were stated.

### 2.4 Real wastewater sample

In order to provide real wastewater, two samples of ammonia containing wastewater were collected from different part of the Pardis Petrochemical Company, Asaluyeh (Iran). Experiments were carried out using ZSAC and BSO under optimal situations and initial pH of the samples remained unchanged. The remaining ammonium ion, COD, BOD_5_, EC, and pH were measured.

### 2.5 Isotherms modeling

Langmuir and Freundlich isotherms were used to study the equilibrium behavior of ammonia adsorption using ZSAC and BSO. The linear form of Langmuir and Freundlich model was used [11], [12]:

(3) $$\text{Langmuir}\;:\;\frac{C_{e}}{q_{e}}=\frac{1}{q_{max}b}+\frac{C_{e}}{q_{max}}$$

(4) $$\text{Freundlich}\;:\;\text{log}q_{e}=\log K_{F}+\frac{1}{n}\log C_{e}$$

where $C_{e}$ is ammonia ion equilibrium concentration (mg/L), and $q_{e}$ is the equilibrium adsorbed ammonia ion per gram of bio-adsorbent (mg/g). As well as,$\;\;q_{max}$ and b are constants of Langmuir model and show the maximum adsorption capacity (mg/g) and adsorption energy (L/g), respectively. Moreover, n and $K_{F}$ are constants of the Freundlich model, which indicate the relationship between adsorption capacity and adsorption intensity. The *n* value indicates adsorb deviation from the linear state. In fact, *n* is a constant that corresponds to the surface heterogeneity. If n = 1, the process is linear; if n > 1, the process is physical and ideal, and n < 1 indicate the process is chemical.

### 2.6 The thermodynamic study

The thermodynamic parameters that include Gibbs free energy (ΔG°), enthalpy (ΔH°), and entropy (ΔS°) can be used to predict adsorption nature. To determine the thermodynamic parameters, the following equations are used [13], [14].

(5) $${\text{K}}_{\text{d}}=\frac{{\text{q}}_{\text{e}}}{{\text{c}}_{\text{e}}}$$

(6) $$\Delta {\text{G}}^{{}^{\circ}}=-{\text{RTLnK}}_{\text{d}}\;$$

(7) $${\text{LnK}}_{\text{d}}=\frac{-\Delta {\text{G}}^{{}^{\circ}}}{\text{RT}}=-\frac{\Delta {\text{H}}^{{}^{\circ}}}{\text{RT}}+\frac{\Delta {\text{S}}^{{}^{\circ}}}{\text{R}}$$
