# Supplementary material for: Dataset of the aqueous solution and petrochemical wastewater treatment containing ammonia using low cost and efficient bio-adsorbents
Source: Data Brief. 2019 Aug 22;26:104308. doi: 10.1016/j.dib.2019.104308 (PMC6727019; doi:10.1016/j.dib.2019.104308)
Supplement: Multimedia component 1 [file mmc1.docx]

**Source data for**

**Dataset of the aqueous solution and petrochemical wastewater treatment containing ammonia using low cost and efficient bio-adsorbents**

Golan Yeganeh ^1^, Bahman Ramavandi ^2,3,^*, Hossein Esmaeili ^4,^*, Sajad Tamjidi ^1^

^1^ Young Researchers and Elite Club, Bushehr Branch, Islamic Azad University, Bushehr, Iran

^2^ Department of Environmental Health Engineering, Faculty of Health and Nutrition, Bushehr University of Medical Sciences, Bushehr, Iran

^3^ Systems Environmental Health and Energy Research Center, The Persian Gulf Biomedical Sciences Research Institute, Bushehr University of Medical Sciences, Bushehr, Iran

^4^ Department of Chemical Engineering, Bushehr Branch, Islamic Azad University, Bushehr, Iran

*** Corresponding authors:** B. Ramavandi (Email: [ramavandi_b@yahoo.com](mailto:ramavandi_b@yahoo.com), [b.ramavandi@bpums.ac.ir](mailto:b.ramavandi@bpums.ac.ir)) and H. Esmaeili (Email: [esmaeili.hossein@gmail.com](mailto:esmaeili.hossein@gmail.com); [esmaeili.hossein@iaubushehr.ac.ir](mailto:esmaeili.hossein@iaubushehr.ac.ir))

Source for Table 1:

| [Summary] |  |  |  |  |  |  |  |  |
| --- | --- | --- | --- | --- | --- | --- | --- | --- |
| File Name | | 1.DAT | | | | | | |
| Date of measurement | | 9/5/2017 | | | | | | |
| Time of measurement | | 6:59:27 | | | | | | |
|  |  |  |  |  |  |  |  |  |
| Sample weight | | 0.0757 | [g] |  | Saturated vapor pressure | | 85.758 | [kPa] |
| Standard volume | | 9.779 | [cm^3^] |  | Adsorption cross section area | | 0.162 | [nm^2^] |
| Dead volume | | 15.946 | [cm^3^] |  | File name of walladsorption | |  |  |
| Equilibrium time | | 0 | [sec] |  | Wall adsorption correction value 1 | |  |  |
| Adsorptive | | N2 |  |  | Wall adsorption correction value 2 | |  |  |
| Apparatus temperature | | 0 | [C] |  |  | |  |  |
| Adsorption temperature | | 77.000 | [K] |  |  | |  |  |
|  |  |  |  |  |  |  |  |  |
| BET plot | | | | | |  |  |  |
| *V_m_* |  |  | 0.9862 | [cm^3^(STP) g^-1^] |  |  |  |  |
| a_s,BET_ |  |  | 112.4641 | [m^2^ g^-1^] |  |  |  |  |
| *C* |  |  | 109.2 |  |  |  |  |  |
| Total pore volume(*p*/*p*_0_=0.990) | |  | 0.9052 | [cm^3^ g^-1^] |  |  |  |  |
| Mean pore diameter | |  | 15.342 | [nm] |  |  |  |  |
| Langmuir plot | | | | | |  |  |  |
| Vm |  |  | 0.9956 | [cm^3^(STP) g^-1^] |  |  |  |  |
| a_s,Lang_ |  |  | 118.5692 | [m^2^ g^-1^] |  |  |  |  |
| B |  |  | 0.4919 |  |  |  |  |  |
| t plot | | | | | |  |  |  |
| Plot data |  | Adsorption branch | |  |  |  |  |  |
| a_1_ |  | 99.21 | | [m^2^ g^-1^] |  |  |  |  |
| V_1_ |  | 0 | | [cm^3^ g^-1^] |  |  |  |  |
| BJH plot | | | | | |  |  |  |
| Plot data |  | Adsorption branch | |  |  |  |  |  |
| V_p_ |  | 0.5362 | | [cm^3^ g^-1^] |  |  |  |  |
| *r_p,peak_*(*Area*) |  | 17.98 | | [nm] |  |  |  |  |
| a_p_ |  | 63.35 | | [m^2^ g^-1^] |  |  |  |  |

| [Summary] |  |  |  |  |  |  |  |  |
| --- | --- | --- | --- | --- | --- | --- | --- | --- |
| File Name | | 2.DAT | | | | | | |
| Date of measurement | | 9/7/2017 | | | | | | |
| Time of measurement | | 7:30:17 | | | | | | |
|  |  |  |  |  |  |  |  |  |
| Sample weight | | 0.0635 | [g] |  | Saturated vapor pressure | | 85.634 | [kPa] |
| Standard volume | | 9.779 | [cm^3^] |  | Adsorption cross section area | | 0.162 | [nm^2^] |
| Dead volume | | 15.869 | [cm^3^] |  | File name of walladsorption | |  |  |
| Equilibrium time | | 0 | [sec] |  | Wall adsorption correction value 1 | |  |  |
| Adsorptive | | N2 |  |  | Wall adsorption correction value 2 | |  |  |
| Apparatus temperature | | 0 | [C] |  |  | |  |  |
| Adsorption temperature | | 77.000 | [K] |  |  | |  |  |
|  |  |  |  |  |  |  |  |  |
| BET plot | | | | | |  |  |  |
| *V_m_* |  |  | 0.4103 | [cm^3^(STP) g^-1^] |  |  |  |  |
| a_s,BET_ |  |  | 45.8236 | [m^2^ g^-1^] |  |  |  |  |
| *C* |  |  | 7.6195 |  |  |  |  |  |
| Total pore volume(*p*/*p*_0_=0.990) | |  | 0.0752361 | [cm^3^ g^-1^] |  |  |  |  |
| Mean pore diameter | |  | 18.254 | [nm] |  |  |  |  |
| Langmuir plot | | | | | |  |  |  |
| Vm |  |  | 0.3814 | [cm^3^(STP) g^-1^] |  |  |  |  |
| a_s,Lang_ |  |  | 62.6601 | [m^2^ g^-1^] |  |  |  |  |
| B |  |  | 0.1468 |  |  |  |  |  |
| t plot | | | | | |  |  |  |
| Plot data |  | Adsorption branch | |  |  |  |  |  |
| a_1_ |  | 23.5623 | | [m^2^ g^-1^] |  |  |  |  |
| V_1_ |  | 0 | | [cm^3^ g^-1^] |  |  |  |  |
| BJH plot | | | | | |  |  |  |
| Plot data |  | Adsorption branch | |  |  |  |  |  |
| V_p_ |  | 0.070222 | | [cm^3^ g^-1^] |  |  |  |  |
| *r_p,peak_*(*Area*) |  | 1.29 | | [nm] |  |  |  |  |
| a_p_ |  | 68.2365 | | [m^2^ g^-1^] |  |  |  |  |

Source for Fig. 1.

| 3.99E+02 | 9.72E+01 |
| --- | --- |
| 4.01E+02 | 9.79E+01 |
| 4.03E+02 | 9.88E+01 |
| 4.05E+02 | 9.94E+01 |
| 4.07E+02 | 9.94E+01 |
| 4.09E+02 | 9.96E+01 |
| 4.11E+02 | 9.98E+01 |
| 4.13E+02 | 9.98E+01 |
| 4.15E+02 | 9.96E+01 |
| 4.17E+02 | 9.94E+01 |
| 4.18E+02 | 9.91E+01 |
| 4.20E+02 | 9.87E+01 |
| 4.22E+02 | 9.83E+01 |
| 4.24E+02 | 9.79E+01 |
| 4.26E+02 | 9.75E+01 |
| 4.28E+02 | 9.72E+01 |
| 4.30E+02 | 9.68E+01 |
| 4.32E+02 | 9.64E+01 |
| 4.34E+02 | 9.61E+01 |
| 4.36E+02 | 9.58E+01 |
| 4.38E+02 | 9.56E+01 |
| 4.40E+02 | 9.53E+01 |
| 4.42E+02 | 9.51E+01 |
| 4.44E+02 | 9.48E+01 |
| 4.45E+02 | 9.46E+01 |
| 4.47E+02 | 9.43E+01 |
| 4.49E+02 | 9.40E+01 |
| 4.51E+02 | 9.37E+01 |
| 4.53E+02 | 9.33E+01 |
| 4.55E+02 | 9.30E+01 |
| 4.57E+02 | 9.26E+01 |
| 4.59E+02 | 9.23E+01 |
| 4.61E+02 | 9.20E+01 |
| 4.63E+02 | 9.16E+01 |
| 4.65E+02 | 9.13E+01 |
| 4.67E+02 | 9.10E+01 |
| 4.69E+02 | 9.07E+01 |
| 4.71E+02 | 9.04E+01 |
| 4.72E+02 | 9.02E+01 |
| 4.74E+02 | 9.01E+01 |
| 4.76E+02 | 9.01E+01 |
| 4.78E+02 | 9.00E+01 |
| 4.80E+02 | 9.00E+01 |
| 4.82E+02 | 9.00E+01 |
| 4.84E+02 | 9.00E+01 |
| 4.86E+02 | 9.00E+01 |
| 4.88E+02 | 9.00E+01 |
| 4.90E+02 | 9.01E+01 |
| 4.92E+02 | 9.01E+01 |
| 4.94E+02 | 9.01E+01 |
| 4.96E+02 | 8.99E+01 |
| 4.98E+02 | 8.97E+01 |
| 4.99E+02 | 8.93E+01 |
| 5.01E+02 | 8.88E+01 |
| 5.03E+02 | 8.81E+01 |
| 5.05E+02 | 8.75E+01 |
| 5.07E+02 | 8.68E+01 |
| 5.09E+02 | 8.62E+01 |
| 5.11E+02 | 8.58E+01 |
| 5.13E+02 | 8.54E+01 |
| 5.15E+02 | 8.53E+01 |
| 5.17E+02 | 8.53E+01 |
| 5.19E+02 | 8.55E+01 |
| 5.21E+02 | 8.59E+01 |
| 5.23E+02 | 8.64E+01 |
| 5.25E+02 | 8.70E+01 |
| 5.26E+02 | 8.77E+01 |
| 5.28E+02 | 8.84E+01 |
| 5.30E+02 | 8.90E+01 |
| 5.32E+02 | 8.94E+01 |
| 5.34E+02 | 8.97E+01 |
| 5.36E+02 | 8.99E+01 |
| 5.38E+02 | 8.99E+01 |
| 5.40E+02 | 8.99E+01 |
| 5.42E+02 | 8.98E+01 |
| 5.44E+02 | 8.96E+01 |
| 5.46E+02 | 8.94E+01 |
| 5.48E+02 | 8.92E+01 |
| 5.50E+02 | 8.89E+01 |
| 5.52E+02 | 8.86E+01 |
| 5.53E+02 | 8.84E+01 |
| 5.55E+02 | 8.81E+01 |
| 5.57E+02 | 8.79E+01 |
| 5.59E+02 | 8.77E+01 |
| 5.61E+02 | 8.75E+01 |
| 5.63E+02 | 8.73E+01 |
| 5.65E+02 | 8.72E+01 |
| 5.67E+02 | 8.71E+01 |
| 5.69E+02 | 8.69E+01 |
| 5.71E+02 | 8.68E+01 |
| 5.73E+02 | 8.67E+01 |
| 5.75E+02 | 8.66E+01 |
| 5.77E+02 | 8.65E+01 |
| 5.79E+02 | 8.64E+01 |
| 5.80E+02 | 8.63E+01 |
| 5.82E+02 | 8.62E+01 |
| 5.84E+02 | 8.60E+01 |
| 5.86E+02 | 8.59E+01 |
| 5.88E+02 | 8.57E+01 |
| 5.90E+02 | 8.56E+01 |
| 5.92E+02 | 8.55E+01 |
| 5.94E+02 | 8.54E+01 |
| 5.96E+02 | 8.53E+01 |
| 5.98E+02 | 8.53E+01 |
| 6.00E+02 | 8.52E+01 |
| 6.02E+02 | 8.51E+01 |
| 6.04E+02 | 8.51E+01 |
| 6.06E+02 | 8.51E+01 |
| 6.07E+02 | 8.51E+01 |
| 6.09E+02 | 8.50E+01 |
| 6.11E+02 | 8.50E+01 |
| 6.13E+02 | 8.51E+01 |
| 6.15E+02 | 8.51E+01 |
| 6.17E+02 | 8.51E+01 |
| 6.19E+02 | 8.51E+01 |
| 6.21E+02 | 8.52E+01 |
| 6.23E+02 | 8.52E+01 |
| 6.25E+02 | 8.53E+01 |
| 6.27E+02 | 8.54E+01 |
| 6.29E+02 | 8.54E+01 |
| 6.31E+02 | 8.55E+01 |
| 6.33E+02 | 8.55E+01 |
| 6.34E+02 | 8.55E+01 |
| 6.36E+02 | 8.56E+01 |
| 6.38E+02 | 8.56E+01 |
| 6.40E+02 | 8.55E+01 |
| 6.42E+02 | 8.55E+01 |
| 6.44E+02 | 8.54E+01 |
| 6.46E+02 | 8.54E+01 |
| 6.48E+02 | 8.52E+01 |
| 6.50E+02 | 8.49E+01 |
| 6.52E+02 | 8.48E+01 |
| 6.54E+02 | 8.48E+01 |
| 6.56E+02 | 8.48E+01 |
| 6.58E+02 | 8.47E+01 |
| 6.60E+02 | 8.47E+01 |
| 6.61E+02 | 8.47E+01 |
| 6.63E+02 | 8.47E+01 |
| 6.65E+02 | 8.48E+01 |
| 6.67E+02 | 8.50E+01 |
| 6.69E+02 | 8.52E+01 |
| 6.71E+02 | 8.54E+01 |
| 6.73E+02 | 8.58E+01 |
| 6.75E+02 | 8.61E+01 |
| 6.77E+02 | 8.65E+01 |
| 6.79E+02 | 8.69E+01 |
| 6.81E+02 | 8.72E+01 |
| 6.83E+02 | 8.74E+01 |
| 6.85E+02 | 8.76E+01 |
| 6.87E+02 | 8.80E+01 |
| 6.88E+02 | 8.84E+01 |
| 6.90E+02 | 8.86E+01 |
| 6.92E+02 | 8.88E+01 |
| 6.94E+02 | 8.90E+01 |
| 6.96E+02 | 8.91E+01 |
| 6.98E+02 | 8.93E+01 |
| 7.00E+02 | 8.94E+01 |
| 7.02E+02 | 8.96E+01 |
| 7.04E+02 | 8.97E+01 |
| 7.06E+02 | 8.99E+01 |
| 7.08E+02 | 9.01E+01 |
| 7.10E+02 | 9.03E+01 |
| 7.12E+02 | 9.04E+01 |
| 7.14E+02 | 9.06E+01 |
| 7.15E+02 | 9.07E+01 |
| 7.17E+02 | 9.08E+01 |
| 7.19E+02 | 9.09E+01 |
| 7.21E+02 | 9.09E+01 |
| 7.23E+02 | 9.10E+01 |
| 7.25E+02 | 9.10E+01 |
| 7.27E+02 | 9.11E+01 |
| 7.29E+02 | 9.11E+01 |
| 7.31E+02 | 9.10E+01 |
| 7.33E+02 | 9.10E+01 |
| 7.35E+02 | 9.09E+01 |
| 7.37E+02 | 9.08E+01 |
| 7.39E+02 | 9.07E+01 |
| 7.41E+02 | 9.05E+01 |
| 7.42E+02 | 9.03E+01 |
| 7.44E+02 | 8.99E+01 |
| 7.46E+02 | 8.95E+01 |
| 7.48E+02 | 8.91E+01 |
| 7.50E+02 | 8.86E+01 |
| 7.52E+02 | 8.82E+01 |
| 7.54E+02 | 8.78E+01 |
| 7.56E+02 | 8.75E+01 |
| 7.58E+02 | 8.73E+01 |
| 7.60E+02 | 8.70E+01 |
| 7.62E+02 | 8.68E+01 |
| 7.64E+02 | 8.63E+01 |
| 7.66E+02 | 8.55E+01 |
| 7.68E+02 | 8.43E+01 |
| 7.69E+02 | 8.29E+01 |
| 7.71E+02 | 8.16E+01 |
| 7.73E+02 | 8.04E+01 |
| 7.75E+02 | 7.94E+01 |
| 7.77E+02 | 7.88E+01 |
| 7.79E+02 | 7.85E+01 |
| 7.81E+02 | 7.85E+01 |
| 7.83E+02 | 7.89E+01 |
| 7.85E+02 | 7.97E+01 |
| 7.87E+02 | 8.09E+01 |
| 7.89E+02 | 8.23E+01 |
| 7.91E+02 | 8.40E+01 |
| 7.93E+02 | 8.57E+01 |
| 7.95E+02 | 8.74E+01 |
| 7.96E+02 | 8.88E+01 |
| 7.98E+02 | 8.98E+01 |
| 8.00E+02 | 9.03E+01 |
| 8.02E+02 | 9.06E+01 |
| 8.04E+02 | 9.08E+01 |
| 8.06E+02 | 9.09E+01 |
| 8.08E+02 | 9.11E+01 |
| 8.10E+02 | 9.13E+01 |
| 8.12E+02 | 9.15E+01 |
| 8.14E+02 | 9.16E+01 |
| 8.16E+02 | 9.18E+01 |
| 8.18E+02 | 9.20E+01 |
| 8.20E+02 | 9.22E+01 |
| 8.22E+02 | 9.23E+01 |
| 8.23E+02 | 9.24E+01 |
| 8.25E+02 | 9.26E+01 |
| 8.27E+02 | 9.27E+01 |
| 8.29E+02 | 9.28E+01 |
| 8.31E+02 | 9.29E+01 |
| 8.33E+02 | 9.30E+01 |
| 8.35E+02 | 9.31E+01 |
| 8.37E+02 | 9.32E+01 |
| 8.39E+02 | 9.33E+01 |
| 8.41E+02 | 9.33E+01 |
| 8.43E+02 | 9.34E+01 |
| 8.45E+02 | 9.34E+01 |
| 8.47E+02 | 9.34E+01 |
| 8.49E+02 | 9.33E+01 |
| 8.50E+02 | 9.33E+01 |
| 8.52E+02 | 9.32E+01 |
| 8.54E+02 | 9.30E+01 |
| 8.56E+02 | 9.28E+01 |
| 8.58E+02 | 9.26E+01 |
| 8.60E+02 | 9.23E+01 |
| 8.62E+02 | 9.20E+01 |
| 8.64E+02 | 9.17E+01 |
| 8.66E+02 | 9.13E+01 |
| 8.68E+02 | 9.10E+01 |
| 8.70E+02 | 9.07E+01 |
| 8.72E+02 | 9.04E+01 |
| 8.74E+02 | 9.02E+01 |
| 8.76E+02 | 8.99E+01 |
| 8.77E+02 | 8.97E+01 |
| 8.79E+02 | 8.95E+01 |
| 8.81E+02 | 8.94E+01 |
| 8.83E+02 | 8.93E+01 |
| 8.85E+02 | 8.92E+01 |
| 8.87E+02 | 8.92E+01 |
| 8.89E+02 | 8.92E+01 |
| 8.91E+02 | 8.92E+01 |
| 8.93E+02 | 8.92E+01 |
| 8.95E+02 | 8.92E+01 |
| 8.97E+02 | 8.91E+01 |
| 8.99E+02 | 8.91E+01 |
| 9.01E+02 | 8.91E+01 |
| 9.03E+02 | 8.90E+01 |
| 9.04E+02 | 8.89E+01 |
| 9.06E+02 | 8.88E+01 |
| 9.08E+02 | 8.87E+01 |
| 9.10E+02 | 8.85E+01 |
| 9.12E+02 | 8.84E+01 |
| 9.14E+02 | 8.82E+01 |
| 9.16E+02 | 8.79E+01 |
| 9.18E+02 | 8.77E+01 |
| 9.20E+02 | 8.74E+01 |
| 9.22E+02 | 8.71E+01 |
| 9.24E+02 | 8.67E+01 |
| 9.26E+02 | 8.64E+01 |
| 9.28E+02 | 8.60E+01 |
| 9.30E+02 | 8.56E+01 |
| 9.31E+02 | 8.52E+01 |
| 9.33E+02 | 8.48E+01 |
| 9.35E+02 | 8.44E+01 |
| 9.37E+02 | 8.39E+01 |
| 9.39E+02 | 8.34E+01 |
| 9.41E+02 | 8.30E+01 |
| 9.43E+02 | 8.25E+01 |
| 9.45E+02 | 8.20E+01 |
| 9.47E+02 | 8.15E+01 |
| 9.49E+02 | 8.10E+01 |
| 9.51E+02 | 8.04E+01 |
| 9.53E+02 | 7.99E+01 |
| 9.55E+02 | 7.94E+01 |
| 9.57E+02 | 7.88E+01 |
| 9.58E+02 | 7.83E+01 |
| 9.60E+02 | 7.77E+01 |
| 9.62E+02 | 7.71E+01 |
| 9.64E+02 | 7.66E+01 |
| 9.66E+02 | 7.60E+01 |
| 9.68E+02 | 7.53E+01 |
| 9.70E+02 | 7.47E+01 |
| 9.72E+02 | 7.40E+01 |
| 9.74E+02 | 7.33E+01 |
| 9.76E+02 | 7.25E+01 |
| 9.78E+02 | 7.17E+01 |
| 9.80E+02 | 7.09E+01 |
| 9.82E+02 | 7.01E+01 |
| 9.84E+02 | 6.93E+01 |
| 9.85E+02 | 6.85E+01 |
| 9.87E+02 | 6.77E+01 |
| 9.89E+02 | 6.70E+01 |
| 9.91E+02 | 6.62E+01 |
| 9.93E+02 | 6.55E+01 |
| 9.95E+02 | 6.47E+01 |
| 9.97E+02 | 6.40E+01 |
| 9.99E+02 | 6.33E+01 |
| 1.00E+03 | 6.25E+01 |
| 1.00E+03 | 6.18E+01 |
| 1.00E+03 | 6.10E+01 |
| 1.01E+03 | 6.02E+01 |
| 1.01E+03 | 5.94E+01 |
| 1.01E+03 | 5.86E+01 |
| 1.01E+03 | 5.78E+01 |
| 1.01E+03 | 5.71E+01 |
| 1.02E+03 | 5.63E+01 |
| 1.02E+03 | 5.55E+01 |
| 1.02E+03 | 5.48E+01 |
| 1.02E+03 | 5.41E+01 |
| 1.02E+03 | 5.34E+01 |
| 1.03E+03 | 5.28E+01 |
| 1.03E+03 | 5.23E+01 |
| 1.03E+03 | 5.18E+01 |
| 1.03E+03 | 5.14E+01 |
| 1.03E+03 | 5.10E+01 |
| 1.04E+03 | 5.06E+01 |
| 1.04E+03 | 5.03E+01 |
| 1.04E+03 | 5.00E+01 |
| 1.04E+03 | 4.97E+01 |
| 1.04E+03 | 4.94E+01 |
| 1.05E+03 | 4.91E+01 |
| 1.05E+03 | 4.88E+01 |
| 1.05E+03 | 4.85E+01 |
| 1.05E+03 | 4.83E+01 |
| 1.05E+03 | 4.80E+01 |
| 1.05E+03 | 4.77E+01 |
| 1.06E+03 | 4.75E+01 |
| 1.06E+03 | 4.74E+01 |
| 1.06E+03 | 4.73E+01 |
| 1.06E+03 | 4.72E+01 |
| 1.06E+03 | 4.72E+01 |
| 1.07E+03 | 4.71E+01 |
| 1.07E+03 | 4.72E+01 |
| 1.07E+03 | 4.72E+01 |
| 1.07E+03 | 4.72E+01 |
| 1.07E+03 | 4.72E+01 |
| 1.08E+03 | 4.72E+01 |
| 1.08E+03 | 4.72E+01 |
| 1.08E+03 | 4.71E+01 |
| 1.08E+03 | 4.70E+01 |
| 1.08E+03 | 4.68E+01 |
| 1.09E+03 | 4.67E+01 |
| 1.09E+03 | 4.64E+01 |
| 1.09E+03 | 4.62E+01 |
| 1.09E+03 | 4.59E+01 |
| 1.09E+03 | 4.57E+01 |
| 1.10E+03 | 4.54E+01 |
| 1.10E+03 | 4.52E+01 |
| 1.10E+03 | 4.50E+01 |
| 1.10E+03 | 4.50E+01 |
| 1.10E+03 | 4.51E+01 |
| 1.11E+03 | 4.53E+01 |
| 1.11E+03 | 4.56E+01 |
| 1.11E+03 | 4.60E+01 |
| 1.11E+03 | 4.65E+01 |
| 1.11E+03 | 4.72E+01 |
| 1.11E+03 | 4.78E+01 |
| 1.12E+03 | 4.85E+01 |
| 1.12E+03 | 4.92E+01 |
| 1.12E+03 | 4.99E+01 |
| 1.12E+03 | 5.06E+01 |
| 1.12E+03 | 5.12E+01 |
| 1.13E+03 | 5.18E+01 |
| 1.13E+03 | 5.23E+01 |
| 1.13E+03 | 5.27E+01 |
| 1.13E+03 | 5.31E+01 |
| 1.13E+03 | 5.34E+01 |
| 1.14E+03 | 5.36E+01 |
| 1.14E+03 | 5.38E+01 |
| 1.14E+03 | 5.39E+01 |
| 1.14E+03 | 5.39E+01 |
| 1.14E+03 | 5.38E+01 |
| 1.15E+03 | 5.37E+01 |
| 1.15E+03 | 5.36E+01 |
| 1.15E+03 | 5.34E+01 |
| 1.15E+03 | 5.31E+01 |
| 1.15E+03 | 5.29E+01 |
| 1.16E+03 | 5.27E+01 |
| 1.16E+03 | 5.26E+01 |
| 1.16E+03 | 5.25E+01 |
| 1.16E+03 | 5.25E+01 |
| 1.16E+03 | 5.25E+01 |
| 1.16E+03 | 5.27E+01 |
| 1.17E+03 | 5.29E+01 |
| 1.17E+03 | 5.32E+01 |
| 1.17E+03 | 5.35E+01 |
| 1.17E+03 | 5.39E+01 |
| 1.17E+03 | 5.43E+01 |
| 1.18E+03 | 5.46E+01 |
| 1.18E+03 | 5.50E+01 |
| 1.18E+03 | 5.53E+01 |
| 1.18E+03 | 5.56E+01 |
| 1.18E+03 | 5.58E+01 |
| 1.19E+03 | 5.60E+01 |
| 1.19E+03 | 5.61E+01 |
| 1.19E+03 | 5.61E+01 |
| 1.19E+03 | 5.61E+01 |
| 1.19E+03 | 5.61E+01 |
| 1.20E+03 | 5.60E+01 |
| 1.20E+03 | 5.60E+01 |
| 1.20E+03 | 5.60E+01 |
| 1.20E+03 | 5.59E+01 |
| 1.20E+03 | 5.59E+01 |
| 1.21E+03 | 5.59E+01 |
| 1.21E+03 | 5.59E+01 |
| 1.21E+03 | 5.59E+01 |
| 1.21E+03 | 5.59E+01 |
| 1.21E+03 | 5.59E+01 |
| 1.21E+03 | 5.58E+01 |
| 1.22E+03 | 5.58E+01 |
| 1.22E+03 | 5.57E+01 |
| 1.22E+03 | 5.56E+01 |
| 1.22E+03 | 5.55E+01 |
| 1.22E+03 | 5.53E+01 |
| 1.23E+03 | 5.51E+01 |
| 1.23E+03 | 5.50E+01 |
| 1.23E+03 | 5.48E+01 |
| 1.23E+03 | 5.47E+01 |
| 1.23E+03 | 5.46E+01 |
| 1.24E+03 | 5.45E+01 |
| 1.24E+03 | 5.44E+01 |
| 1.24E+03 | 5.43E+01 |
| 1.24E+03 | 5.42E+01 |
| 1.24E+03 | 5.42E+01 |
| 1.25E+03 | 5.42E+01 |
| 1.25E+03 | 5.42E+01 |
| 1.25E+03 | 5.42E+01 |
| 1.25E+03 | 5.42E+01 |
| 1.25E+03 | 5.42E+01 |
| 1.26E+03 | 5.42E+01 |
| 1.26E+03 | 5.41E+01 |
| 1.26E+03 | 5.41E+01 |
| 1.26E+03 | 5.41E+01 |
| 1.26E+03 | 5.41E+01 |
| 1.27E+03 | 5.41E+01 |
| 1.27E+03 | 5.41E+01 |
| 1.27E+03 | 5.41E+01 |
| 1.27E+03 | 5.41E+01 |
| 1.27E+03 | 5.41E+01 |
| 1.27E+03 | 5.41E+01 |
| 1.28E+03 | 5.40E+01 |
| 1.28E+03 | 5.40E+01 |
| 1.28E+03 | 5.39E+01 |
| 1.28E+03 | 5.37E+01 |
| 1.28E+03 | 5.36E+01 |
| 1.29E+03 | 5.33E+01 |
| 1.29E+03 | 5.30E+01 |
| 1.29E+03 | 5.26E+01 |
| 1.29E+03 | 5.22E+01 |
| 1.29E+03 | 5.15E+01 |
| 1.30E+03 | 5.07E+01 |
| 1.30E+03 | 4.96E+01 |
| 1.30E+03 | 4.82E+01 |
| 1.30E+03 | 4.64E+01 |
| 1.30E+03 | 4.44E+01 |
| 1.31E+03 | 4.23E+01 |
| 1.31E+03 | 4.03E+01 |
| 1.31E+03 | 3.84E+01 |
| 1.31E+03 | 3.68E+01 |
| 1.31E+03 | 3.56E+01 |
| 1.32E+03 | 3.48E+01 |
| 1.32E+03 | 3.45E+01 |
| 1.32E+03 | 3.46E+01 |
| 1.32E+03 | 3.52E+01 |
| 1.32E+03 | 3.63E+01 |
| 1.32E+03 | 3.77E+01 |
| 1.33E+03 | 3.94E+01 |
| 1.33E+03 | 4.12E+01 |
| 1.33E+03 | 4.29E+01 |
| 1.33E+03 | 4.45E+01 |
| 1.33E+03 | 4.58E+01 |
| 1.34E+03 | 4.69E+01 |
| 1.34E+03 | 4.78E+01 |
| 1.34E+03 | 4.84E+01 |
| 1.34E+03 | 4.90E+01 |
| 1.34E+03 | 4.93E+01 |
| 1.35E+03 | 4.95E+01 |
| 1.35E+03 | 4.96E+01 |
| 1.35E+03 | 4.95E+01 |
| 1.35E+03 | 4.92E+01 |
| 1.35E+03 | 4.88E+01 |
| 1.36E+03 | 4.84E+01 |
| 1.36E+03 | 4.79E+01 |
| 1.36E+03 | 4.74E+01 |
| 1.36E+03 | 4.69E+01 |
| 1.36E+03 | 4.63E+01 |
| 1.37E+03 | 4.57E+01 |
| 1.37E+03 | 4.51E+01 |
| 1.37E+03 | 4.45E+01 |
| 1.37E+03 | 4.40E+01 |
| 1.37E+03 | 4.36E+01 |
| 1.38E+03 | 4.32E+01 |
| 1.38E+03 | 4.29E+01 |
| 1.38E+03 | 4.28E+01 |
| 1.38E+03 | 4.28E+01 |
| 1.38E+03 | 4.29E+01 |
| 1.38E+03 | 4.29E+01 |
| 1.39E+03 | 4.31E+01 |
| 1.39E+03 | 4.32E+01 |
| 1.39E+03 | 4.34E+01 |
| 1.39E+03 | 4.36E+01 |
| 1.39E+03 | 4.38E+01 |
| 1.40E+03 | 4.38E+01 |
| 1.40E+03 | 4.38E+01 |
| 1.40E+03 | 4.37E+01 |
| 1.40E+03 | 4.37E+01 |
| 1.40E+03 | 4.36E+01 |
| 1.41E+03 | 4.34E+01 |
| 1.41E+03 | 4.32E+01 |
| 1.41E+03 | 4.30E+01 |
| 1.41E+03 | 4.28E+01 |
| 1.41E+03 | 4.27E+01 |
| 1.42E+03 | 4.26E+01 |
| 1.42E+03 | 4.25E+01 |
| 1.42E+03 | 4.24E+01 |
| 1.42E+03 | 4.24E+01 |
| 1.42E+03 | 4.23E+01 |
| 1.43E+03 | 4.23E+01 |
| 1.43E+03 | 4.22E+01 |
| 1.43E+03 | 4.22E+01 |
| 1.43E+03 | 4.22E+01 |
| 1.43E+03 | 4.22E+01 |
| 1.43E+03 | 4.22E+01 |
| 1.44E+03 | 4.23E+01 |
| 1.44E+03 | 4.23E+01 |
| 1.44E+03 | 4.24E+01 |
| 1.44E+03 | 4.26E+01 |
| 1.44E+03 | 4.27E+01 |
| 1.45E+03 | 4.28E+01 |
| 1.45E+03 | 4.29E+01 |
| 1.45E+03 | 4.31E+01 |
| 1.45E+03 | 4.33E+01 |
| 1.45E+03 | 4.36E+01 |
| 1.46E+03 | 4.40E+01 |
| 1.46E+03 | 4.45E+01 |
| 1.46E+03 | 4.51E+01 |
| 1.46E+03 | 4.59E+01 |
| 1.46E+03 | 4.68E+01 |
| 1.47E+03 | 4.77E+01 |
| 1.47E+03 | 4.87E+01 |
| 1.47E+03 | 4.97E+01 |
| 1.47E+03 | 5.07E+01 |
| 1.47E+03 | 5.17E+01 |
| 1.48E+03 | 5.27E+01 |
| 1.48E+03 | 5.34E+01 |
| 1.48E+03 | 5.40E+01 |
| 1.48E+03 | 5.45E+01 |
| 1.48E+03 | 5.48E+01 |
| 1.48E+03 | 5.49E+01 |
| 1.49E+03 | 5.48E+01 |
| 1.49E+03 | 5.47E+01 |
| 1.49E+03 | 5.47E+01 |
| 1.49E+03 | 5.46E+01 |
| 1.49E+03 | 5.44E+01 |
| 1.50E+03 | 5.41E+01 |
| 1.50E+03 | 5.37E+01 |
| 1.50E+03 | 5.32E+01 |
| 1.50E+03 | 5.27E+01 |
| 1.50E+03 | 5.23E+01 |
| 1.51E+03 | 5.18E+01 |
| 1.51E+03 | 5.14E+01 |
| 1.51E+03 | 5.09E+01 |
| 1.51E+03 | 5.05E+01 |
| 1.51E+03 | 5.00E+01 |
| 1.52E+03 | 4.95E+01 |
| 1.52E+03 | 4.91E+01 |
| 1.52E+03 | 4.85E+01 |
| 1.52E+03 | 4.79E+01 |
| 1.52E+03 | 4.76E+01 |
| 1.53E+03 | 4.73E+01 |
| 1.53E+03 | 4.67E+01 |
| 1.53E+03 | 4.60E+01 |
| 1.53E+03 | 4.52E+01 |
| 1.53E+03 | 4.43E+01 |
| 1.54E+03 | 4.33E+01 |
| 1.54E+03 | 4.23E+01 |
| 1.54E+03 | 4.12E+01 |
| 1.54E+03 | 4.01E+01 |
| 1.54E+03 | 3.93E+01 |
| 1.54E+03 | 3.83E+01 |
| 1.55E+03 | 3.72E+01 |
| 1.55E+03 | 3.61E+01 |
| 1.55E+03 | 3.50E+01 |
| 1.55E+03 | 3.40E+01 |
| 1.55E+03 | 3.30E+01 |
| 1.56E+03 | 3.21E+01 |
| 1.56E+03 | 3.13E+01 |
| 1.56E+03 | 3.06E+01 |
| 1.56E+03 | 2.98E+01 |
| 1.56E+03 | 2.90E+01 |
| 1.57E+03 | 2.82E+01 |
| 1.57E+03 | 2.74E+01 |
| 1.57E+03 | 2.66E+01 |
| 1.57E+03 | 2.59E+01 |
| 1.57E+03 | 2.52E+01 |
| 1.58E+03 | 2.47E+01 |
| 1.58E+03 | 2.43E+01 |
| 1.58E+03 | 2.36E+01 |
| 1.58E+03 | 2.29E+01 |
| 1.58E+03 | 2.22E+01 |
| 1.59E+03 | 2.15E+01 |
| 1.59E+03 | 2.08E+01 |
| 1.59E+03 | 2.00E+01 |
| 1.59E+03 | 1.91E+01 |
| 1.59E+03 | 1.83E+01 |
| 1.59E+03 | 1.75E+01 |
| 1.60E+03 | 1.65E+01 |
| 1.60E+03 | 1.56E+01 |
| 1.60E+03 | 1.48E+01 |
| 1.60E+03 | 1.39E+01 |
| 1.60E+03 | 1.31E+01 |
| 1.61E+03 | 1.24E+01 |
| 1.61E+03 | 1.18E+01 |
| 1.61E+03 | 1.13E+01 |
| 1.61E+03 | 1.10E+01 |
| 1.61E+03 | 1.08E+01 |
| 1.62E+03 | 1.07E+01 |
| 1.62E+03 | 1.08E+01 |
| 1.62E+03 | 1.11E+01 |
| 1.62E+03 | 1.15E+01 |
| 1.62E+03 | 1.20E+01 |
| 1.63E+03 | 1.26E+01 |
| 1.63E+03 | 1.32E+01 |
| 1.63E+03 | 1.40E+01 |
| 1.63E+03 | 1.48E+01 |
| 1.63E+03 | 1.57E+01 |
| 1.64E+03 | 1.66E+01 |
| 1.64E+03 | 1.76E+01 |
| 1.64E+03 | 1.85E+01 |
| 1.64E+03 | 1.95E+01 |
| 1.64E+03 | 2.03E+01 |
| 1.65E+03 | 2.12E+01 |
| 1.65E+03 | 2.21E+01 |
| 1.65E+03 | 2.29E+01 |
| 1.65E+03 | 2.37E+01 |
| 1.65E+03 | 2.47E+01 |
| 1.65E+03 | 2.57E+01 |
| 1.66E+03 | 2.66E+01 |
| 1.66E+03 | 2.77E+01 |
| 1.66E+03 | 2.86E+01 |
| 1.66E+03 | 2.97E+01 |
| 1.66E+03 | 3.07E+01 |
| 1.67E+03 | 3.18E+01 |
| 1.67E+03 | 3.31E+01 |
| 1.67E+03 | 3.44E+01 |
| 1.67E+03 | 3.57E+01 |
| 1.67E+03 | 3.68E+01 |
| 1.68E+03 | 3.78E+01 |
| 1.68E+03 | 3.87E+01 |
| 1.68E+03 | 3.96E+01 |
| 1.68E+03 | 4.03E+01 |
| 1.68E+03 | 4.11E+01 |
| 1.69E+03 | 4.18E+01 |
| 1.69E+03 | 4.23E+01 |
| 1.69E+03 | 4.28E+01 |
| 1.69E+03 | 4.32E+01 |
| 1.69E+03 | 4.36E+01 |
| 1.70E+03 | 4.38E+01 |
| 1.70E+03 | 4.40E+01 |
| 1.70E+03 | 4.43E+01 |
| 1.70E+03 | 4.48E+01 |
| 1.70E+03 | 4.54E+01 |
| 1.70E+03 | 4.57E+01 |
| 1.71E+03 | 4.62E+01 |
| 1.71E+03 | 4.67E+01 |
| 1.71E+03 | 4.71E+01 |
| 1.71E+03 | 4.77E+01 |
| 1.71E+03 | 4.83E+01 |
| 1.72E+03 | 4.90E+01 |
| 1.72E+03 | 5.00E+01 |
| 1.72E+03 | 5.07E+01 |
| 1.72E+03 | 5.15E+01 |
| 1.72E+03 | 5.24E+01 |
| 1.73E+03 | 5.32E+01 |
| 1.73E+03 | 5.40E+01 |
| 1.73E+03 | 5.49E+01 |
| 1.73E+03 | 5.59E+01 |
| 1.73E+03 | 5.70E+01 |
| 1.74E+03 | 5.82E+01 |
| 1.74E+03 | 5.93E+01 |
| 1.74E+03 | 6.04E+01 |
| 1.74E+03 | 6.16E+01 |
| 1.74E+03 | 6.27E+01 |
| 1.75E+03 | 6.40E+01 |
| 1.75E+03 | 6.53E+01 |
| 1.75E+03 | 6.66E+01 |
| 1.75E+03 | 6.79E+01 |
| 1.75E+03 | 6.92E+01 |
| 1.75E+03 | 7.04E+01 |
| 1.76E+03 | 7.17E+01 |
| 1.76E+03 | 7.28E+01 |
| 1.76E+03 | 7.38E+01 |
| 1.76E+03 | 7.48E+01 |
| 1.76E+03 | 7.58E+01 |
| 1.77E+03 | 7.67E+01 |
| 1.77E+03 | 7.77E+01 |
| 1.77E+03 | 7.85E+01 |
| 1.77E+03 | 7.92E+01 |
| 1.77E+03 | 8.00E+01 |
| 1.78E+03 | 8.08E+01 |
| 1.78E+03 | 8.16E+01 |
| 1.78E+03 | 8.24E+01 |
| 1.78E+03 | 8.31E+01 |
| 1.78E+03 | 8.38E+01 |
| 1.79E+03 | 8.46E+01 |
| 1.79E+03 | 8.53E+01 |
| 1.79E+03 | 8.61E+01 |
| 1.79E+03 | 8.67E+01 |
| 1.79E+03 | 8.73E+01 |
| 1.80E+03 | 8.79E+01 |
| 1.80E+03 | 8.85E+01 |
| 1.80E+03 | 8.90E+01 |
| 1.80E+03 | 8.94E+01 |
| 1.80E+03 | 8.99E+01 |
| 1.81E+03 | 9.03E+01 |
| 1.81E+03 | 9.07E+01 |
| 1.81E+03 | 9.11E+01 |
| 1.81E+03 | 9.16E+01 |
| 1.81E+03 | 9.20E+01 |
| 1.81E+03 | 9.24E+01 |
| 1.82E+03 | 9.27E+01 |
| 1.82E+03 | 9.31E+01 |
| 1.82E+03 | 9.34E+01 |
| 1.82E+03 | 9.36E+01 |
| 1.82E+03 | 9.38E+01 |
| 1.83E+03 | 9.41E+01 |
| 1.83E+03 | 9.44E+01 |
| 1.83E+03 | 9.46E+01 |
| 1.83E+03 | 9.48E+01 |
| 1.83E+03 | 9.49E+01 |
| 1.84E+03 | 9.50E+01 |
| 1.84E+03 | 9.51E+01 |
| 1.84E+03 | 9.52E+01 |
| 1.84E+03 | 9.53E+01 |
| 1.84E+03 | 9.53E+01 |
| 1.85E+03 | 9.54E+01 |
| 1.85E+03 | 9.55E+01 |
| 1.85E+03 | 9.55E+01 |
| 1.85E+03 | 9.56E+01 |
| 1.85E+03 | 9.56E+01 |
| 1.86E+03 | 9.57E+01 |
| 1.86E+03 | 9.57E+01 |
| 1.86E+03 | 9.57E+01 |
| 1.86E+03 | 9.58E+01 |
| 1.86E+03 | 9.59E+01 |
| 1.86E+03 | 9.58E+01 |
| 1.87E+03 | 9.58E+01 |
| 1.87E+03 | 9.58E+01 |
| 1.87E+03 | 9.58E+01 |
| 1.87E+03 | 9.57E+01 |
| 1.87E+03 | 9.57E+01 |
| 1.88E+03 | 9.57E+01 |
| 1.88E+03 | 9.57E+01 |
| 1.88E+03 | 9.56E+01 |
| 1.88E+03 | 9.56E+01 |
| 1.88E+03 | 9.56E+01 |
| 1.89E+03 | 9.56E+01 |
| 1.89E+03 | 9.56E+01 |
| 1.89E+03 | 9.56E+01 |
| 1.89E+03 | 9.55E+01 |
| 1.89E+03 | 9.55E+01 |
| 1.90E+03 | 9.54E+01 |
| 1.90E+03 | 9.54E+01 |
| 1.90E+03 | 9.53E+01 |
| 1.90E+03 | 9.53E+01 |
| 1.90E+03 | 9.52E+01 |
| 1.91E+03 | 9.52E+01 |
| 1.91E+03 | 9.52E+01 |
| 1.91E+03 | 9.51E+01 |
| 1.91E+03 | 9.51E+01 |
| 1.91E+03 | 9.51E+01 |
| 1.92E+03 | 9.50E+01 |
| 1.92E+03 | 9.50E+01 |
| 1.92E+03 | 9.50E+01 |
| 1.92E+03 | 9.49E+01 |
| 1.92E+03 | 9.49E+01 |
| 1.92E+03 | 9.49E+01 |
| 1.93E+03 | 9.49E+01 |
| 1.93E+03 | 9.49E+01 |
| 1.93E+03 | 9.48E+01 |
| 1.93E+03 | 9.48E+01 |
| 1.93E+03 | 9.48E+01 |
| 1.94E+03 | 9.48E+01 |
| 1.94E+03 | 9.47E+01 |
| 1.94E+03 | 9.47E+01 |
| 1.94E+03 | 9.47E+01 |
| 1.94E+03 | 9.46E+01 |
| 1.95E+03 | 9.45E+01 |
| 1.95E+03 | 9.45E+01 |
| 1.95E+03 | 9.44E+01 |
| 1.95E+03 | 9.44E+01 |
| 1.95E+03 | 9.44E+01 |
| 1.96E+03 | 9.43E+01 |
| 1.96E+03 | 9.43E+01 |
| 1.96E+03 | 9.42E+01 |
| 1.96E+03 | 9.42E+01 |
| 1.96E+03 | 9.42E+01 |
| 1.97E+03 | 9.41E+01 |
| 1.97E+03 | 9.41E+01 |
| 1.97E+03 | 9.40E+01 |
| 1.97E+03 | 9.40E+01 |
| 1.97E+03 | 9.39E+01 |
| 1.97E+03 | 9.39E+01 |
| 1.98E+03 | 9.38E+01 |
| 1.98E+03 | 9.38E+01 |
| 1.98E+03 | 9.37E+01 |
| 1.98E+03 | 9.37E+01 |
| 1.98E+03 | 9.37E+01 |
| 1.99E+03 | 9.36E+01 |
| 1.99E+03 | 9.36E+01 |
| 1.99E+03 | 9.35E+01 |
| 1.99E+03 | 9.34E+01 |
| 1.99E+03 | 9.34E+01 |
| 2.00E+03 | 9.33E+01 |
| 2.00E+03 | 9.33E+01 |
| 2.00E+03 | 9.32E+01 |
| 2.00E+03 | 9.31E+01 |
| 2.00E+03 | 9.31E+01 |
| 2.01E+03 | 9.30E+01 |
| 2.01E+03 | 9.29E+01 |
| 2.01E+03 | 9.29E+01 |
| 2.01E+03 | 9.28E+01 |
| 2.01E+03 | 9.28E+01 |
| 2.02E+03 | 9.27E+01 |
| 2.02E+03 | 9.26E+01 |
| 2.02E+03 | 9.26E+01 |
| 2.02E+03 | 9.25E+01 |
| 2.02E+03 | 9.24E+01 |
| 2.02E+03 | 9.24E+01 |
| 2.03E+03 | 9.23E+01 |
| 2.03E+03 | 9.23E+01 |
| 2.03E+03 | 9.22E+01 |
| 2.03E+03 | 9.21E+01 |
| 2.03E+03 | 9.21E+01 |
| 2.04E+03 | 9.20E+01 |
| 2.04E+03 | 9.20E+01 |
| 2.04E+03 | 9.19E+01 |
| 2.04E+03 | 9.19E+01 |
| 2.04E+03 | 9.18E+01 |
| 2.05E+03 | 9.18E+01 |
| 2.05E+03 | 9.17E+01 |
| 2.05E+03 | 9.16E+01 |
| 2.05E+03 | 9.16E+01 |
| 2.05E+03 | 9.15E+01 |
| 2.06E+03 | 9.15E+01 |
| 2.06E+03 | 9.14E+01 |
| 2.06E+03 | 9.14E+01 |
| 2.06E+03 | 9.13E+01 |
| 2.06E+03 | 9.13E+01 |
| 2.07E+03 | 9.12E+01 |
| 2.07E+03 | 9.11E+01 |
| 2.07E+03 | 9.11E+01 |
| 2.07E+03 | 9.10E+01 |
| 2.07E+03 | 9.10E+01 |
| 2.08E+03 | 9.09E+01 |
| 2.08E+03 | 9.09E+01 |
| 2.08E+03 | 9.08E+01 |
| 2.08E+03 | 9.08E+01 |
| 2.08E+03 | 9.07E+01 |
| 2.08E+03 | 9.07E+01 |
| 2.09E+03 | 9.06E+01 |
| 2.09E+03 | 9.06E+01 |
| 2.09E+03 | 9.05E+01 |
| 2.09E+03 | 9.05E+01 |
| 2.09E+03 | 9.04E+01 |
| 2.10E+03 | 9.04E+01 |
| 2.10E+03 | 9.03E+01 |
| 2.10E+03 | 9.02E+01 |
| 2.10E+03 | 9.02E+01 |
| 2.10E+03 | 9.01E+01 |
| 2.11E+03 | 9.01E+01 |
| 2.11E+03 | 9.00E+01 |
| 2.11E+03 | 9.00E+01 |
| 2.11E+03 | 8.99E+01 |
| 2.11E+03 | 8.99E+01 |
| 2.12E+03 | 8.98E+01 |
| 2.12E+03 | 8.97E+01 |
| 2.12E+03 | 8.97E+01 |
| 2.12E+03 | 8.96E+01 |
| 2.12E+03 | 8.96E+01 |
| 2.13E+03 | 8.95E+01 |
| 2.13E+03 | 8.95E+01 |
| 2.13E+03 | 8.94E+01 |
| 2.13E+03 | 8.94E+01 |
| 2.13E+03 | 8.93E+01 |
| 2.13E+03 | 8.93E+01 |
| 2.14E+03 | 8.92E+01 |
| 2.14E+03 | 8.92E+01 |
| 2.14E+03 | 8.91E+01 |
| 2.14E+03 | 8.91E+01 |
| 2.14E+03 | 8.90E+01 |
| 2.15E+03 | 8.90E+01 |
| 2.15E+03 | 8.89E+01 |
| 2.15E+03 | 8.89E+01 |
| 2.15E+03 | 8.88E+01 |
| 2.15E+03 | 8.88E+01 |
| 2.16E+03 | 8.87E+01 |
| 2.16E+03 | 8.87E+01 |
| 2.16E+03 | 8.86E+01 |
| 2.16E+03 | 8.86E+01 |
| 2.16E+03 | 8.85E+01 |
| 2.17E+03 | 8.85E+01 |
| 2.17E+03 | 8.84E+01 |
| 2.17E+03 | 8.83E+01 |
| 2.17E+03 | 8.83E+01 |
| 2.17E+03 | 8.82E+01 |
| 2.18E+03 | 8.82E+01 |
| 2.18E+03 | 8.81E+01 |
| 2.18E+03 | 8.81E+01 |
| 2.18E+03 | 8.80E+01 |
| 2.18E+03 | 8.80E+01 |
| 2.19E+03 | 8.79E+01 |
| 2.19E+03 | 8.79E+01 |
| 2.19E+03 | 8.78E+01 |
| 2.19E+03 | 8.77E+01 |
| 2.19E+03 | 8.77E+01 |
| 2.19E+03 | 8.76E+01 |
| 2.20E+03 | 8.76E+01 |
| 2.20E+03 | 8.75E+01 |
| 2.20E+03 | 8.75E+01 |
| 2.20E+03 | 8.74E+01 |
| 2.20E+03 | 8.74E+01 |
| 2.21E+03 | 8.73E+01 |
| 2.21E+03 | 8.73E+01 |
| 2.21E+03 | 8.72E+01 |
| 2.21E+03 | 8.72E+01 |
| 2.21E+03 | 8.72E+01 |
| 2.22E+03 | 8.71E+01 |
| 2.22E+03 | 8.71E+01 |
| 2.22E+03 | 8.71E+01 |
| 2.22E+03 | 8.70E+01 |
| 2.22E+03 | 8.70E+01 |
| 2.23E+03 | 8.70E+01 |
| 2.23E+03 | 8.69E+01 |
| 2.23E+03 | 8.69E+01 |
| 2.23E+03 | 8.69E+01 |
| 2.23E+03 | 8.69E+01 |
| 2.24E+03 | 8.68E+01 |
| 2.24E+03 | 8.68E+01 |
| 2.24E+03 | 8.68E+01 |
| 2.24E+03 | 8.68E+01 |
| 2.24E+03 | 8.67E+01 |
| 2.24E+03 | 8.67E+01 |
| 2.25E+03 | 8.67E+01 |
| 2.25E+03 | 8.67E+01 |
| 2.25E+03 | 8.66E+01 |
| 2.25E+03 | 8.66E+01 |
| 2.25E+03 | 8.66E+01 |
| 2.26E+03 | 8.65E+01 |
| 2.26E+03 | 8.65E+01 |
| 2.26E+03 | 8.65E+01 |
| 2.26E+03 | 8.64E+01 |
| 2.26E+03 | 8.64E+01 |
| 2.27E+03 | 8.63E+01 |
| 2.27E+03 | 8.63E+01 |
| 2.27E+03 | 8.62E+01 |
| 2.27E+03 | 8.61E+01 |
| 2.27E+03 | 8.60E+01 |
| 2.28E+03 | 8.60E+01 |
| 2.28E+03 | 8.59E+01 |
| 2.28E+03 | 8.59E+01 |
| 2.28E+03 | 8.58E+01 |
| 2.28E+03 | 8.58E+01 |
| 2.29E+03 | 8.58E+01 |
| 2.29E+03 | 8.58E+01 |
| 2.29E+03 | 8.58E+01 |
| 2.29E+03 | 8.58E+01 |
| 2.29E+03 | 8.59E+01 |
| 2.29E+03 | 8.60E+01 |
| 2.30E+03 | 8.61E+01 |
| 2.30E+03 | 8.63E+01 |
| 2.30E+03 | 8.64E+01 |
| 2.30E+03 | 8.66E+01 |
| 2.30E+03 | 8.68E+01 |
| 2.31E+03 | 8.70E+01 |
| 2.31E+03 | 8.73E+01 |
| 2.31E+03 | 8.76E+01 |
| 2.31E+03 | 8.80E+01 |
| 2.31E+03 | 8.83E+01 |
| 2.32E+03 | 8.87E+01 |
| 2.32E+03 | 8.90E+01 |
| 2.32E+03 | 8.93E+01 |
| 2.32E+03 | 8.96E+01 |
| 2.32E+03 | 8.98E+01 |
| 2.33E+03 | 9.00E+01 |
| 2.33E+03 | 9.03E+01 |
| 2.33E+03 | 9.05E+01 |
| 2.33E+03 | 9.06E+01 |
| 2.33E+03 | 9.04E+01 |
| 2.34E+03 | 9.02E+01 |
| 2.34E+03 | 9.00E+01 |
| 2.34E+03 | 8.99E+01 |
| 2.34E+03 | 8.99E+01 |
| 2.34E+03 | 9.00E+01 |
| 2.35E+03 | 9.02E+01 |
| 2.35E+03 | 9.06E+01 |
| 2.35E+03 | 9.10E+01 |
| 2.35E+03 | 9.14E+01 |
| 2.35E+03 | 9.18E+01 |
| 2.35E+03 | 9.21E+01 |
| 2.36E+03 | 9.24E+01 |
| 2.36E+03 | 9.25E+01 |
| 2.36E+03 | 9.26E+01 |
| 2.36E+03 | 9.24E+01 |
| 2.36E+03 | 9.20E+01 |
| 2.37E+03 | 9.12E+01 |
| 2.37E+03 | 9.03E+01 |
| 2.37E+03 | 8.92E+01 |
| 2.37E+03 | 8.82E+01 |
| 2.37E+03 | 8.73E+01 |
| 2.38E+03 | 8.64E+01 |
| 2.38E+03 | 8.56E+01 |
| 2.38E+03 | 8.49E+01 |
| 2.38E+03 | 8.44E+01 |
| 2.38E+03 | 8.39E+01 |
| 2.39E+03 | 8.36E+01 |
| 2.39E+03 | 8.33E+01 |
| 2.39E+03 | 8.31E+01 |
| 2.39E+03 | 8.30E+01 |
| 2.39E+03 | 8.29E+01 |
| 2.40E+03 | 8.28E+01 |
| 2.40E+03 | 8.28E+01 |
| 2.40E+03 | 8.27E+01 |
| 2.40E+03 | 8.27E+01 |
| 2.40E+03 | 8.26E+01 |
| 2.40E+03 | 8.25E+01 |
| 2.41E+03 | 8.25E+01 |
| 2.41E+03 | 8.24E+01 |
| 2.41E+03 | 8.23E+01 |
| 2.41E+03 | 8.23E+01 |
| 2.41E+03 | 8.22E+01 |
| 2.42E+03 | 8.22E+01 |
| 2.42E+03 | 8.21E+01 |
| 2.42E+03 | 8.21E+01 |
| 2.42E+03 | 8.20E+01 |
| 2.42E+03 | 8.19E+01 |
| 2.43E+03 | 8.19E+01 |
| 2.43E+03 | 8.18E+01 |
| 2.43E+03 | 8.18E+01 |
| 2.43E+03 | 8.17E+01 |
| 2.43E+03 | 8.17E+01 |
| 2.44E+03 | 8.16E+01 |
| 2.44E+03 | 8.15E+01 |
| 2.44E+03 | 8.15E+01 |
| 2.44E+03 | 8.14E+01 |
| 2.44E+03 | 8.14E+01 |
| 2.45E+03 | 8.13E+01 |
| 2.45E+03 | 8.13E+01 |
| 2.45E+03 | 8.12E+01 |
| 2.45E+03 | 8.11E+01 |
| 2.45E+03 | 8.11E+01 |
| 2.46E+03 | 8.10E+01 |
| 2.46E+03 | 8.10E+01 |
| 2.46E+03 | 8.09E+01 |
| 2.46E+03 | 8.09E+01 |
| 2.46E+03 | 8.08E+01 |
| 2.46E+03 | 8.07E+01 |
| 2.47E+03 | 8.07E+01 |
| 2.47E+03 | 8.06E+01 |
| 2.47E+03 | 8.06E+01 |
| 2.47E+03 | 8.05E+01 |
| 2.47E+03 | 8.04E+01 |
| 2.48E+03 | 8.04E+01 |
| 2.48E+03 | 8.03E+01 |
| 2.48E+03 | 8.03E+01 |
| 2.48E+03 | 8.02E+01 |
| 2.48E+03 | 8.01E+01 |
| 2.49E+03 | 8.01E+01 |
| 2.49E+03 | 8.00E+01 |
| 2.49E+03 | 7.99E+01 |
| 2.49E+03 | 7.98E+01 |
| 2.49E+03 | 7.98E+01 |
| 2.50E+03 | 7.97E+01 |
| 2.50E+03 | 7.96E+01 |
| 2.50E+03 | 7.96E+01 |
| 2.50E+03 | 7.95E+01 |
| 2.50E+03 | 7.94E+01 |
| 2.51E+03 | 7.94E+01 |
| 2.51E+03 | 7.93E+01 |
| 2.51E+03 | 7.93E+01 |
| 2.51E+03 | 7.92E+01 |
| 2.51E+03 | 7.92E+01 |
| 2.51E+03 | 7.91E+01 |
| 2.52E+03 | 7.90E+01 |
| 2.52E+03 | 7.90E+01 |
| 2.52E+03 | 7.89E+01 |
| 2.52E+03 | 7.89E+01 |
| 2.52E+03 | 7.88E+01 |
| 2.53E+03 | 7.88E+01 |
| 2.53E+03 | 7.87E+01 |
| 2.53E+03 | 7.87E+01 |
| 2.53E+03 | 7.86E+01 |
| 2.53E+03 | 7.86E+01 |
| 2.54E+03 | 7.85E+01 |
| 2.54E+03 | 7.85E+01 |
| 2.54E+03 | 7.84E+01 |
| 2.54E+03 | 7.84E+01 |
| 2.54E+03 | 7.83E+01 |
| 2.55E+03 | 7.83E+01 |
| 2.55E+03 | 7.82E+01 |
| 2.55E+03 | 7.82E+01 |
| 2.55E+03 | 7.81E+01 |
| 2.55E+03 | 7.81E+01 |
| 2.56E+03 | 7.80E+01 |
| 2.56E+03 | 7.80E+01 |
| 2.56E+03 | 7.79E+01 |
| 2.56E+03 | 7.79E+01 |
| 2.56E+03 | 7.78E+01 |
| 2.56E+03 | 7.78E+01 |
| 2.57E+03 | 7.77E+01 |
| 2.57E+03 | 7.76E+01 |
| 2.57E+03 | 7.76E+01 |
| 2.57E+03 | 7.75E+01 |
| 2.57E+03 | 7.75E+01 |
| 2.58E+03 | 7.74E+01 |
| 2.58E+03 | 7.74E+01 |
| 2.58E+03 | 7.73E+01 |
| 2.58E+03 | 7.73E+01 |
| 2.58E+03 | 7.72E+01 |
| 2.59E+03 | 7.71E+01 |
| 2.59E+03 | 7.71E+01 |
| 2.59E+03 | 7.70E+01 |
| 2.59E+03 | 7.70E+01 |
| 2.59E+03 | 7.69E+01 |
| 2.60E+03 | 7.69E+01 |
| 2.60E+03 | 7.68E+01 |
| 2.60E+03 | 7.68E+01 |
| 2.60E+03 | 7.67E+01 |
| 2.60E+03 | 7.67E+01 |
| 2.61E+03 | 7.66E+01 |
| 2.61E+03 | 7.66E+01 |
| 2.61E+03 | 7.65E+01 |
| 2.61E+03 | 7.65E+01 |
| 2.61E+03 | 7.64E+01 |
| 2.62E+03 | 7.64E+01 |
| 2.62E+03 | 7.63E+01 |
| 2.62E+03 | 7.63E+01 |
| 2.62E+03 | 7.62E+01 |
| 2.62E+03 | 7.62E+01 |
| 2.62E+03 | 7.61E+01 |
| 2.63E+03 | 7.61E+01 |
| 2.63E+03 | 7.60E+01 |
| 2.63E+03 | 7.60E+01 |
| 2.63E+03 | 7.59E+01 |
| 2.63E+03 | 7.58E+01 |
| 2.64E+03 | 7.58E+01 |
| 2.64E+03 | 7.57E+01 |
| 2.64E+03 | 7.57E+01 |
| 2.64E+03 | 7.56E+01 |
| 2.64E+03 | 7.55E+01 |
| 2.65E+03 | 7.55E+01 |
| 2.65E+03 | 7.54E+01 |
| 2.65E+03 | 7.53E+01 |
| 2.65E+03 | 7.53E+01 |
| 2.65E+03 | 7.52E+01 |
| 2.66E+03 | 7.52E+01 |
| 2.66E+03 | 7.51E+01 |
| 2.66E+03 | 7.50E+01 |
| 2.66E+03 | 7.50E+01 |
| 2.66E+03 | 7.49E+01 |
| 2.67E+03 | 7.48E+01 |
| 2.67E+03 | 7.48E+01 |
| 2.67E+03 | 7.47E+01 |
| 2.67E+03 | 7.46E+01 |
| 2.67E+03 | 7.46E+01 |
| 2.67E+03 | 7.45E+01 |
| 2.68E+03 | 7.44E+01 |
| 2.68E+03 | 7.44E+01 |
| 2.68E+03 | 7.43E+01 |
| 2.68E+03 | 7.42E+01 |
| 2.68E+03 | 7.42E+01 |
| 2.69E+03 | 7.41E+01 |
| 2.69E+03 | 7.41E+01 |
| 2.69E+03 | 7.40E+01 |
| 2.69E+03 | 7.40E+01 |
| 2.69E+03 | 7.39E+01 |
| 2.70E+03 | 7.38E+01 |
| 2.70E+03 | 7.38E+01 |
| 2.70E+03 | 7.37E+01 |
| 2.70E+03 | 7.36E+01 |
| 2.70E+03 | 7.36E+01 |
| 2.71E+03 | 7.35E+01 |
| 2.71E+03 | 7.34E+01 |
| 2.71E+03 | 7.33E+01 |
| 2.71E+03 | 7.33E+01 |
| 2.71E+03 | 7.32E+01 |
| 2.72E+03 | 7.31E+01 |
| 2.72E+03 | 7.30E+01 |
| 2.72E+03 | 7.29E+01 |
| 2.72E+03 | 7.29E+01 |
| 2.72E+03 | 7.28E+01 |
| 2.73E+03 | 7.27E+01 |
| 2.73E+03 | 7.27E+01 |
| 2.73E+03 | 7.26E+01 |
| 2.73E+03 | 7.25E+01 |
| 2.73E+03 | 7.25E+01 |
| 2.73E+03 | 7.24E+01 |
| 2.74E+03 | 7.24E+01 |
| 2.74E+03 | 7.23E+01 |
| 2.74E+03 | 7.23E+01 |
| 2.74E+03 | 7.22E+01 |
| 2.74E+03 | 7.22E+01 |
| 2.75E+03 | 7.21E+01 |
| 2.75E+03 | 7.20E+01 |
| 2.75E+03 | 7.20E+01 |
| 2.75E+03 | 7.19E+01 |
| 2.75E+03 | 7.18E+01 |
| 2.76E+03 | 7.17E+01 |
| 2.76E+03 | 7.17E+01 |
| 2.76E+03 | 7.16E+01 |
| 2.76E+03 | 7.15E+01 |
| 2.76E+03 | 7.14E+01 |
| 2.77E+03 | 7.13E+01 |
| 2.77E+03 | 7.12E+01 |
| 2.77E+03 | 7.11E+01 |
| 2.77E+03 | 7.10E+01 |
| 2.77E+03 | 7.09E+01 |
| 2.78E+03 | 7.08E+01 |
| 2.78E+03 | 7.07E+01 |
| 2.78E+03 | 7.06E+01 |
| 2.78E+03 | 7.05E+01 |
| 2.78E+03 | 7.04E+01 |
| 2.78E+03 | 7.03E+01 |
| 2.79E+03 | 7.01E+01 |
| 2.79E+03 | 7.00E+01 |
| 2.79E+03 | 6.99E+01 |
| 2.79E+03 | 6.97E+01 |
| 2.79E+03 | 6.96E+01 |
| 2.80E+03 | 6.94E+01 |
| 2.80E+03 | 6.92E+01 |
| 2.80E+03 | 6.91E+01 |
| 2.80E+03 | 6.89E+01 |
| 2.80E+03 | 6.87E+01 |
| 2.81E+03 | 6.85E+01 |
| 2.81E+03 | 6.83E+01 |
| 2.81E+03 | 6.81E+01 |
| 2.81E+03 | 6.79E+01 |
| 2.81E+03 | 6.77E+01 |
| 2.82E+03 | 6.75E+01 |
| 2.82E+03 | 6.73E+01 |
| 2.82E+03 | 6.70E+01 |
| 2.82E+03 | 6.67E+01 |
| 2.82E+03 | 6.64E+01 |
| 2.83E+03 | 6.60E+01 |
| 2.83E+03 | 6.56E+01 |
| 2.83E+03 | 6.51E+01 |
| 2.83E+03 | 6.46E+01 |
| 2.83E+03 | 6.39E+01 |
| 2.83E+03 | 6.31E+01 |
| 2.84E+03 | 6.21E+01 |
| 2.84E+03 | 6.09E+01 |
| 2.84E+03 | 5.97E+01 |
| 2.84E+03 | 5.84E+01 |
| 2.84E+03 | 5.72E+01 |
| 2.85E+03 | 5.60E+01 |
| 2.85E+03 | 5.50E+01 |
| 2.85E+03 | 5.43E+01 |
| 2.85E+03 | 5.37E+01 |
| 2.85E+03 | 5.34E+01 |
| 2.86E+03 | 5.32E+01 |
| 2.86E+03 | 5.33E+01 |
| 2.86E+03 | 5.35E+01 |
| 2.86E+03 | 5.38E+01 |
| 2.86E+03 | 5.43E+01 |
| 2.87E+03 | 5.48E+01 |
| 2.87E+03 | 5.53E+01 |
| 2.87E+03 | 5.57E+01 |
| 2.87E+03 | 5.60E+01 |
| 2.87E+03 | 5.62E+01 |
| 2.88E+03 | 5.63E+01 |
| 2.88E+03 | 5.63E+01 |
| 2.88E+03 | 5.62E+01 |
| 2.88E+03 | 5.61E+01 |
| 2.88E+03 | 5.60E+01 |
| 2.89E+03 | 5.57E+01 |
| 2.89E+03 | 5.54E+01 |
| 2.89E+03 | 5.49E+01 |
| 2.89E+03 | 5.44E+01 |
| 2.89E+03 | 5.38E+01 |
| 2.89E+03 | 5.32E+01 |
| 2.90E+03 | 5.26E+01 |
| 2.90E+03 | 5.19E+01 |
| 2.90E+03 | 5.12E+01 |
| 2.90E+03 | 5.04E+01 |
| 2.90E+03 | 4.95E+01 |
| 2.91E+03 | 4.85E+01 |
| 2.91E+03 | 4.74E+01 |
| 2.91E+03 | 4.62E+01 |
| 2.91E+03 | 4.49E+01 |
| 2.91E+03 | 4.37E+01 |
| 2.92E+03 | 4.25E+01 |
| 2.92E+03 | 4.15E+01 |
| 2.92E+03 | 4.07E+01 |
| 2.92E+03 | 4.01E+01 |
| 2.92E+03 | 3.99E+01 |
| 2.93E+03 | 3.99E+01 |
| 2.93E+03 | 4.04E+01 |
| 2.93E+03 | 4.11E+01 |
| 2.93E+03 | 4.20E+01 |
| 2.93E+03 | 4.32E+01 |
| 2.94E+03 | 4.44E+01 |
| 2.94E+03 | 4.56E+01 |
| 2.94E+03 | 4.67E+01 |
| 2.94E+03 | 4.77E+01 |
| 2.94E+03 | 4.85E+01 |
| 2.94E+03 | 4.91E+01 |
| 2.95E+03 | 4.95E+01 |
| 2.95E+03 | 4.99E+01 |
| 2.95E+03 | 5.01E+01 |
| 2.95E+03 | 5.03E+01 |
| 2.95E+03 | 5.06E+01 |
| 2.96E+03 | 5.09E+01 |
| 2.96E+03 | 5.13E+01 |
| 2.96E+03 | 5.19E+01 |
| 2.96E+03 | 5.25E+01 |
| 2.96E+03 | 5.33E+01 |
| 2.97E+03 | 5.41E+01 |
| 2.97E+03 | 5.50E+01 |
| 2.97E+03 | 5.60E+01 |
| 2.97E+03 | 5.68E+01 |
| 2.97E+03 | 5.77E+01 |
| 2.98E+03 | 5.85E+01 |
| 2.98E+03 | 5.92E+01 |
| 2.98E+03 | 5.98E+01 |
| 2.98E+03 | 6.03E+01 |
| 2.98E+03 | 6.07E+01 |
| 2.99E+03 | 6.11E+01 |
| 2.99E+03 | 6.14E+01 |
| 2.99E+03 | 6.16E+01 |
| 2.99E+03 | 6.18E+01 |
| 2.99E+03 | 6.20E+01 |
| 3.00E+03 | 6.21E+01 |
| 3.00E+03 | 6.22E+01 |
| 3.00E+03 | 6.22E+01 |
| 3.00E+03 | 6.23E+01 |
| 3.00E+03 | 6.23E+01 |
| 3.00E+03 | 6.23E+01 |
| 3.01E+03 | 6.22E+01 |
| 3.01E+03 | 6.22E+01 |
| 3.01E+03 | 6.21E+01 |
| 3.01E+03 | 6.21E+01 |
| 3.01E+03 | 6.20E+01 |
| 3.02E+03 | 6.19E+01 |
| 3.02E+03 | 6.18E+01 |
| 3.02E+03 | 6.17E+01 |
| 3.02E+03 | 6.16E+01 |
| 3.02E+03 | 6.15E+01 |
| 3.03E+03 | 6.14E+01 |
| 3.03E+03 | 6.12E+01 |
| 3.03E+03 | 6.11E+01 |
| 3.03E+03 | 6.10E+01 |
| 3.03E+03 | 6.09E+01 |
| 3.04E+03 | 6.07E+01 |
| 3.04E+03 | 6.06E+01 |
| 3.04E+03 | 6.05E+01 |
| 3.04E+03 | 6.04E+01 |
| 3.04E+03 | 6.02E+01 |
| 3.05E+03 | 6.01E+01 |
| 3.05E+03 | 6.00E+01 |
| 3.05E+03 | 5.98E+01 |
| 3.05E+03 | 5.97E+01 |
| 3.05E+03 | 5.96E+01 |
| 3.05E+03 | 5.95E+01 |
| 3.06E+03 | 5.94E+01 |
| 3.06E+03 | 5.93E+01 |
| 3.06E+03 | 5.93E+01 |
| 3.06E+03 | 5.92E+01 |
| 3.06E+03 | 5.91E+01 |
| 3.07E+03 | 5.91E+01 |
| 3.07E+03 | 5.91E+01 |
| 3.07E+03 | 5.91E+01 |
| 3.07E+03 | 5.91E+01 |
| 3.07E+03 | 5.91E+01 |
| 3.08E+03 | 5.91E+01 |
| 3.08E+03 | 5.91E+01 |
| 3.08E+03 | 5.91E+01 |
| 3.08E+03 | 5.91E+01 |
| 3.08E+03 | 5.91E+01 |
| 3.09E+03 | 5.91E+01 |
| 3.09E+03 | 5.91E+01 |
| 3.09E+03 | 5.91E+01 |
| 3.09E+03 | 5.91E+01 |
| 3.09E+03 | 5.91E+01 |
| 3.10E+03 | 5.91E+01 |
| 3.10E+03 | 5.90E+01 |
| 3.10E+03 | 5.90E+01 |
| 3.10E+03 | 5.90E+01 |
| 3.10E+03 | 5.89E+01 |
| 3.10E+03 | 5.89E+01 |
| 3.11E+03 | 5.88E+01 |
| 3.11E+03 | 5.88E+01 |
| 3.11E+03 | 5.87E+01 |
| 3.11E+03 | 5.86E+01 |
| 3.11E+03 | 5.86E+01 |
| 3.12E+03 | 5.85E+01 |
| 3.12E+03 | 5.85E+01 |
| 3.12E+03 | 5.84E+01 |
| 3.12E+03 | 5.83E+01 |
| 3.12E+03 | 5.82E+01 |
| 3.13E+03 | 5.82E+01 |
| 3.13E+03 | 5.81E+01 |
| 3.13E+03 | 5.80E+01 |
| 3.13E+03 | 5.79E+01 |
| 3.13E+03 | 5.78E+01 |
| 3.14E+03 | 5.78E+01 |
| 3.14E+03 | 5.77E+01 |
| 3.14E+03 | 5.76E+01 |
| 3.14E+03 | 5.75E+01 |
| 3.14E+03 | 5.74E+01 |
| 3.15E+03 | 5.73E+01 |
| 3.15E+03 | 5.72E+01 |
| 3.15E+03 | 5.71E+01 |
| 3.15E+03 | 5.70E+01 |
| 3.15E+03 | 5.69E+01 |
| 3.16E+03 | 5.68E+01 |
| 3.16E+03 | 5.67E+01 |
| 3.16E+03 | 5.66E+01 |
| 3.16E+03 | 5.65E+01 |
| 3.16E+03 | 5.64E+01 |
| 3.16E+03 | 5.63E+01 |
| 3.17E+03 | 5.62E+01 |
| 3.17E+03 | 5.61E+01 |
| 3.17E+03 | 5.59E+01 |
| 3.17E+03 | 5.58E+01 |
| 3.17E+03 | 5.57E+01 |
| 3.18E+03 | 5.55E+01 |
| 3.18E+03 | 5.54E+01 |
| 3.18E+03 | 5.52E+01 |
| 3.18E+03 | 5.51E+01 |
| 3.18E+03 | 5.49E+01 |
| 3.19E+03 | 5.48E+01 |
| 3.19E+03 | 5.46E+01 |
| 3.19E+03 | 5.45E+01 |
| 3.19E+03 | 5.43E+01 |
| 3.19E+03 | 5.42E+01 |
| 3.20E+03 | 5.40E+01 |
| 3.20E+03 | 5.38E+01 |
| 3.20E+03 | 5.37E+01 |
| 3.20E+03 | 5.35E+01 |
| 3.20E+03 | 5.33E+01 |
| 3.21E+03 | 5.32E+01 |
| 3.21E+03 | 5.30E+01 |
| 3.21E+03 | 5.28E+01 |
| 3.21E+03 | 5.26E+01 |
| 3.21E+03 | 5.24E+01 |
| 3.21E+03 | 5.23E+01 |
| 3.22E+03 | 5.21E+01 |
| 3.22E+03 | 5.19E+01 |
| 3.22E+03 | 5.17E+01 |
| 3.22E+03 | 5.16E+01 |
| 3.22E+03 | 5.14E+01 |
| 3.23E+03 | 5.12E+01 |
| 3.23E+03 | 5.10E+01 |
| 3.23E+03 | 5.09E+01 |
| 3.23E+03 | 5.07E+01 |
| 3.23E+03 | 5.05E+01 |
| 3.24E+03 | 5.04E+01 |
| 3.24E+03 | 5.02E+01 |
| 3.24E+03 | 5.00E+01 |
| 3.24E+03 | 4.99E+01 |
| 3.24E+03 | 4.97E+01 |
| 3.25E+03 | 4.96E+01 |
| 3.25E+03 | 4.94E+01 |
| 3.25E+03 | 4.93E+01 |
| 3.25E+03 | 4.92E+01 |
| 3.25E+03 | 4.90E+01 |
| 3.26E+03 | 4.89E+01 |
| 3.26E+03 | 4.88E+01 |
| 3.26E+03 | 4.87E+01 |
| 3.26E+03 | 4.85E+01 |
| 3.26E+03 | 4.84E+01 |
| 3.27E+03 | 4.83E+01 |
| 3.27E+03 | 4.82E+01 |
| 3.27E+03 | 4.81E+01 |
| 3.27E+03 | 4.80E+01 |
| 3.27E+03 | 4.79E+01 |
| 3.27E+03 | 4.78E+01 |
| 3.28E+03 | 4.77E+01 |
| 3.28E+03 | 4.77E+01 |
| 3.28E+03 | 4.76E+01 |
| 3.28E+03 | 4.75E+01 |
| 3.28E+03 | 4.74E+01 |
| 3.29E+03 | 4.73E+01 |
| 3.29E+03 | 4.72E+01 |
| 3.29E+03 | 4.71E+01 |
| 3.29E+03 | 4.70E+01 |
| 3.29E+03 | 4.68E+01 |
| 3.30E+03 | 4.67E+01 |
| 3.30E+03 | 4.65E+01 |
| 3.30E+03 | 4.64E+01 |
| 3.30E+03 | 4.62E+01 |
| 3.30E+03 | 4.60E+01 |
| 3.31E+03 | 4.59E+01 |
| 3.31E+03 | 4.57E+01 |
| 3.31E+03 | 4.55E+01 |
| 3.31E+03 | 4.53E+01 |
| 3.31E+03 | 4.51E+01 |
| 3.32E+03 | 4.49E+01 |
| 3.32E+03 | 4.46E+01 |
| 3.32E+03 | 4.44E+01 |
| 3.32E+03 | 4.42E+01 |
| 3.32E+03 | 4.40E+01 |
| 3.32E+03 | 4.38E+01 |
| 3.33E+03 | 4.36E+01 |
| 3.33E+03 | 4.34E+01 |
| 3.33E+03 | 4.32E+01 |
| 3.33E+03 | 4.30E+01 |
| 3.33E+03 | 4.28E+01 |
| 3.34E+03 | 4.27E+01 |
| 3.34E+03 | 4.26E+01 |
| 3.34E+03 | 4.25E+01 |
| 3.34E+03 | 4.24E+01 |
| 3.34E+03 | 4.23E+01 |
| 3.35E+03 | 4.22E+01 |
| 3.35E+03 | 4.22E+01 |
| 3.35E+03 | 4.21E+01 |
| 3.35E+03 | 4.21E+01 |
| 3.35E+03 | 4.21E+01 |
| 3.36E+03 | 4.21E+01 |
| 3.36E+03 | 4.20E+01 |
| 3.36E+03 | 4.20E+01 |
| 3.36E+03 | 4.20E+01 |
| 3.36E+03 | 4.20E+01 |
| 3.37E+03 | 4.20E+01 |
| 3.37E+03 | 4.20E+01 |
| 3.37E+03 | 4.19E+01 |
| 3.37E+03 | 4.19E+01 |
| 3.37E+03 | 4.19E+01 |
| 3.37E+03 | 4.18E+01 |
| 3.38E+03 | 4.18E+01 |
| 3.38E+03 | 4.18E+01 |
| 3.38E+03 | 4.17E+01 |
| 3.38E+03 | 4.17E+01 |
| 3.38E+03 | 4.17E+01 |
| 3.39E+03 | 4.16E+01 |
| 3.39E+03 | 4.16E+01 |
| 3.39E+03 | 4.15E+01 |
| 3.39E+03 | 4.15E+01 |
| 3.39E+03 | 4.14E+01 |
| 3.40E+03 | 4.13E+01 |
| 3.40E+03 | 4.13E+01 |
| 3.40E+03 | 4.12E+01 |
| 3.40E+03 | 4.11E+01 |
| 3.40E+03 | 4.10E+01 |
| 3.41E+03 | 4.09E+01 |
| 3.41E+03 | 4.08E+01 |
| 3.41E+03 | 4.07E+01 |
| 3.41E+03 | 4.06E+01 |
| 3.41E+03 | 4.05E+01 |
| 3.42E+03 | 4.05E+01 |
| 3.42E+03 | 4.04E+01 |
| 3.42E+03 | 4.04E+01 |
| 3.42E+03 | 4.04E+01 |
| 3.42E+03 | 4.04E+01 |
| 3.43E+03 | 4.04E+01 |
| 3.43E+03 | 4.05E+01 |
| 3.43E+03 | 4.06E+01 |
| 3.43E+03 | 4.07E+01 |
| 3.43E+03 | 4.08E+01 |
| 3.43E+03 | 4.10E+01 |
| 3.44E+03 | 4.12E+01 |
| 3.44E+03 | 4.13E+01 |
| 3.44E+03 | 4.15E+01 |
| 3.44E+03 | 4.17E+01 |
| 3.44E+03 | 4.20E+01 |
| 3.45E+03 | 4.22E+01 |
| 3.45E+03 | 4.25E+01 |
| 3.45E+03 | 4.27E+01 |
| 3.45E+03 | 4.30E+01 |
| 3.45E+03 | 4.32E+01 |
| 3.46E+03 | 4.35E+01 |
| 3.46E+03 | 4.37E+01 |
| 3.46E+03 | 4.40E+01 |
| 3.46E+03 | 4.42E+01 |
| 3.46E+03 | 4.45E+01 |
| 3.47E+03 | 4.47E+01 |
| 3.47E+03 | 4.49E+01 |
| 3.47E+03 | 4.52E+01 |
| 3.47E+03 | 4.54E+01 |
| 3.47E+03 | 4.56E+01 |
| 3.48E+03 | 4.59E+01 |
| 3.48E+03 | 4.61E+01 |
| 3.48E+03 | 4.63E+01 |
| 3.48E+03 | 4.66E+01 |
| 3.48E+03 | 4.68E+01 |
| 3.48E+03 | 4.71E+01 |
| 3.49E+03 | 4.74E+01 |
| 3.49E+03 | 4.77E+01 |
| 3.49E+03 | 4.81E+01 |
| 3.49E+03 | 4.84E+01 |
| 3.49E+03 | 4.88E+01 |
| 3.50E+03 | 4.91E+01 |
| 3.50E+03 | 4.95E+01 |
| 3.50E+03 | 4.99E+01 |
| 3.50E+03 | 5.03E+01 |
| 3.50E+03 | 5.07E+01 |
| 3.51E+03 | 5.12E+01 |
| 3.51E+03 | 5.16E+01 |
| 3.51E+03 | 5.21E+01 |
| 3.51E+03 | 5.26E+01 |
| 3.51E+03 | 5.30E+01 |
| 3.52E+03 | 5.35E+01 |
| 3.52E+03 | 5.40E+01 |
| 3.52E+03 | 5.45E+01 |
| 3.52E+03 | 5.49E+01 |
| 3.52E+03 | 5.54E+01 |
| 3.53E+03 | 5.58E+01 |
| 3.53E+03 | 5.63E+01 |
| 3.53E+03 | 5.68E+01 |
| 3.53E+03 | 5.73E+01 |
| 3.53E+03 | 5.77E+01 |
| 3.54E+03 | 5.82E+01 |
| 3.54E+03 | 5.87E+01 |
| 3.54E+03 | 5.91E+01 |
| 3.54E+03 | 5.96E+01 |
| 3.54E+03 | 6.01E+01 |
| 3.54E+03 | 6.05E+01 |
| 3.55E+03 | 6.10E+01 |
| 3.55E+03 | 6.14E+01 |
| 3.55E+03 | 6.20E+01 |
| 3.55E+03 | 6.26E+01 |
| 3.55E+03 | 6.30E+01 |
| 3.56E+03 | 6.34E+01 |
| 3.56E+03 | 6.38E+01 |
| 3.56E+03 | 6.43E+01 |
| 3.56E+03 | 6.47E+01 |
| 3.56E+03 | 6.52E+01 |
| 3.57E+03 | 6.56E+01 |
| 3.57E+03 | 6.60E+01 |
| 3.57E+03 | 6.65E+01 |
| 3.57E+03 | 6.71E+01 |
| 3.57E+03 | 6.76E+01 |
| 3.58E+03 | 6.80E+01 |
| 3.58E+03 | 6.84E+01 |
| 3.58E+03 | 6.89E+01 |
| 3.58E+03 | 6.94E+01 |
| 3.58E+03 | 7.00E+01 |
| 3.59E+03 | 7.06E+01 |
| 3.59E+03 | 7.10E+01 |
| 3.59E+03 | 7.13E+01 |
| 3.59E+03 | 7.19E+01 |
| 3.59E+03 | 7.23E+01 |
| 3.59E+03 | 7.28E+01 |
| 3.60E+03 | 7.33E+01 |
| 3.60E+03 | 7.37E+01 |
| 3.60E+03 | 7.41E+01 |
| 3.60E+03 | 7.47E+01 |
| 3.60E+03 | 7.53E+01 |
| 3.61E+03 | 7.57E+01 |
| 3.61E+03 | 7.60E+01 |
| 3.61E+03 | 7.63E+01 |
| 3.61E+03 | 7.70E+01 |
| 3.61E+03 | 7.75E+01 |
| 3.62E+03 | 7.79E+01 |
| 3.62E+03 | 7.83E+01 |
| 3.62E+03 | 7.86E+01 |
| 3.62E+03 | 7.90E+01 |
| 3.62E+03 | 7.94E+01 |
| 3.63E+03 | 7.98E+01 |
| 3.63E+03 | 8.01E+01 |
| 3.63E+03 | 8.04E+01 |
| 3.63E+03 | 8.11E+01 |
| 3.63E+03 | 8.18E+01 |
| 3.64E+03 | 8.23E+01 |
| 3.64E+03 | 8.27E+01 |
| 3.64E+03 | 8.31E+01 |
| 3.64E+03 | 8.33E+01 |
| 3.64E+03 | 8.36E+01 |
| 3.64E+03 | 8.40E+01 |
| 3.65E+03 | 8.45E+01 |
| 3.65E+03 | 8.47E+01 |
| 3.65E+03 | 8.49E+01 |
| 3.65E+03 | 8.53E+01 |
| 3.65E+03 | 8.56E+01 |
| 3.66E+03 | 8.59E+01 |
| 3.66E+03 | 8.65E+01 |
| 3.66E+03 | 8.70E+01 |
| 3.66E+03 | 8.74E+01 |
| 3.66E+03 | 8.79E+01 |
| 3.67E+03 | 8.84E+01 |
| 3.67E+03 | 8.86E+01 |
| 3.67E+03 | 8.87E+01 |
| 3.67E+03 | 8.90E+01 |
| 3.67E+03 | 8.94E+01 |
| 3.68E+03 | 8.96E+01 |
| 3.68E+03 | 8.96E+01 |
| 3.68E+03 | 8.97E+01 |
| 3.68E+03 | 8.96E+01 |
| 3.68E+03 | 8.97E+01 |
| 3.69E+03 | 9.00E+01 |
| 3.69E+03 | 9.02E+01 |
| 3.69E+03 | 9.03E+01 |
| 3.69E+03 | 9.05E+01 |
| 3.69E+03 | 9.10E+01 |
| 3.70E+03 | 9.13E+01 |
| 3.70E+03 | 9.14E+01 |
| 3.70E+03 | 9.14E+01 |
| 3.70E+03 | 9.12E+01 |
| 3.70E+03 | 9.11E+01 |
| 3.70E+03 | 9.12E+01 |
| 3.71E+03 | 9.15E+01 |
| 3.71E+03 | 9.16E+01 |
| 3.71E+03 | 9.15E+01 |
| 3.71E+03 | 9.13E+01 |
| 3.71E+03 | 9.13E+01 |
| 3.72E+03 | 9.13E+01 |
| 3.72E+03 | 9.16E+01 |
| 3.72E+03 | 9.18E+01 |
| 3.72E+03 | 9.18E+01 |
| 3.72E+03 | 9.18E+01 |
| 3.73E+03 | 9.18E+01 |
| 3.73E+03 | 9.23E+01 |
| 3.73E+03 | 9.24E+01 |
| 3.73E+03 | 9.24E+01 |
| 3.73E+03 | 9.24E+01 |
| 3.74E+03 | 9.24E+01 |
| 3.74E+03 | 9.23E+01 |
| 3.74E+03 | 9.22E+01 |
| 3.74E+03 | 9.20E+01 |
| 3.74E+03 | 9.19E+01 |
| 3.75E+03 | 9.16E+01 |
| 3.75E+03 | 9.13E+01 |
| 3.75E+03 | 9.13E+01 |
| 3.75E+03 | 9.14E+01 |
| 3.75E+03 | 9.15E+01 |
| 3.75E+03 | 9.15E+01 |
| 3.76E+03 | 9.14E+01 |
| 3.76E+03 | 9.12E+01 |
| 3.76E+03 | 9.13E+01 |
| 3.76E+03 | 9.16E+01 |
| 3.76E+03 | 9.15E+01 |
| 3.77E+03 | 9.16E+01 |
| 3.77E+03 | 9.18E+01 |
| 3.77E+03 | 9.17E+01 |
| 3.77E+03 | 9.16E+01 |
| 3.77E+03 | 9.15E+01 |
| 3.78E+03 | 9.14E+01 |
| 3.78E+03 | 9.13E+01 |
| 3.78E+03 | 9.12E+01 |
| 3.78E+03 | 9.12E+01 |
| 3.78E+03 | 9.14E+01 |
| 3.79E+03 | 9.16E+01 |
| 3.79E+03 | 9.16E+01 |
| 3.79E+03 | 9.18E+01 |
| 3.79E+03 | 9.18E+01 |
| 3.79E+03 | 9.17E+01 |
| 3.80E+03 | 9.16E+01 |
| 3.80E+03 | 9.16E+01 |
| 3.80E+03 | 9.16E+01 |
| 3.80E+03 | 9.15E+01 |
| 3.80E+03 | 9.16E+01 |
| 3.81E+03 | 9.18E+01 |
| 3.81E+03 | 9.17E+01 |
| 3.81E+03 | 9.17E+01 |
| 3.81E+03 | 9.16E+01 |
| 3.81E+03 | 9.15E+01 |
| 3.81E+03 | 9.16E+01 |
| 3.82E+03 | 9.15E+01 |
| 3.82E+03 | 9.17E+01 |
| 3.82E+03 | 9.18E+01 |
| 3.82E+03 | 9.19E+01 |
| 3.82E+03 | 9.22E+01 |
| 3.83E+03 | 9.22E+01 |
| 3.83E+03 | 9.20E+01 |
| 3.83E+03 | 9.18E+01 |
| 3.83E+03 | 9.16E+01 |
| 3.83E+03 | 9.15E+01 |
| 3.84E+03 | 9.18E+01 |
| 3.84E+03 | 9.24E+01 |
| 3.84E+03 | 9.25E+01 |
| 3.84E+03 | 9.23E+01 |
| 3.84E+03 | 9.22E+01 |
| 3.85E+03 | 9.20E+01 |
| 3.85E+03 | 9.19E+01 |
| 3.85E+03 | 9.18E+01 |
| 3.85E+03 | 9.17E+01 |
| 3.85E+03 | 9.19E+01 |
| 3.86E+03 | 9.21E+01 |
| 3.86E+03 | 9.23E+01 |
| 3.86E+03 | 9.23E+01 |
| 3.86E+03 | 9.21E+01 |
| 3.86E+03 | 9.19E+01 |
| 3.86E+03 | 9.17E+01 |
| 3.87E+03 | 9.16E+01 |
| 3.87E+03 | 9.20E+01 |
| 3.87E+03 | 9.24E+01 |
| 3.87E+03 | 9.24E+01 |
| 3.87E+03 | 9.23E+01 |
| 3.88E+03 | 9.22E+01 |
| 3.88E+03 | 9.21E+01 |
| 3.88E+03 | 9.20E+01 |
| 3.88E+03 | 9.20E+01 |
| 3.88E+03 | 9.20E+01 |
| 3.89E+03 | 9.23E+01 |
| 3.89E+03 | 9.26E+01 |
| 3.89E+03 | 9.25E+01 |
| 3.89E+03 | 9.25E+01 |
| 3.89E+03 | 9.23E+01 |
| 3.90E+03 | 9.21E+01 |
| 3.90E+03 | 9.21E+01 |
| 3.90E+03 | 9.21E+01 |
| 3.90E+03 | 9.21E+01 |
| 3.90E+03 | 9.23E+01 |
| 3.91E+03 | 9.22E+01 |
| 3.91E+03 | 9.22E+01 |
| 3.91E+03 | 9.23E+01 |
| 3.91E+03 | 9.22E+01 |
| 3.91E+03 | 9.21E+01 |
| 3.91E+03 | 9.22E+01 |
| 3.92E+03 | 9.24E+01 |
| 3.92E+03 | 9.25E+01 |
| 3.92E+03 | 9.27E+01 |
| 3.92E+03 | 9.27E+01 |
| 3.92E+03 | 9.26E+01 |
| 3.93E+03 | 9.25E+01 |
| 3.93E+03 | 9.24E+01 |
| 3.93E+03 | 9.24E+01 |
| 3.93E+03 | 9.25E+01 |
| 3.93E+03 | 9.25E+01 |
| 3.94E+03 | 9.26E+01 |
| 3.94E+03 | 9.26E+01 |
| 3.94E+03 | 9.26E+01 |
| 3.94E+03 | 9.26E+01 |
| 3.94E+03 | 9.26E+01 |
| 3.95E+03 | 9.26E+01 |
| 3.95E+03 | 9.26E+01 |
| 3.95E+03 | 9.27E+01 |
| 3.95E+03 | 9.26E+01 |
| 3.95E+03 | 9.26E+01 |
| 3.96E+03 | 9.26E+01 |
| 3.96E+03 | 9.26E+01 |
| 3.96E+03 | 9.26E+01 |
| 3.96E+03 | 9.27E+01 |
| 3.96E+03 | 9.27E+01 |
| 3.97E+03 | 9.27E+01 |
| 3.97E+03 | 9.28E+01 |
| 3.97E+03 | 9.28E+01 |
| 3.97E+03 | 9.28E+01 |
| 3.97E+03 | 9.28E+01 |
| 3.97E+03 | 9.28E+01 |
| 3.98E+03 | 9.28E+01 |
| 3.98E+03 | 9.29E+01 |
| 3.98E+03 | 9.29E+01 |
| 3.98E+03 | 9.29E+01 |
| 3.98E+03 | 9.29E+01 |
| 3.99E+03 | 9.30E+01 |
| 3.99E+03 | 9.30E+01 |
| 3.99E+03 | 9.30E+01 |
| 3.99E+03 | 9.30E+01 |
| 3.99E+03 | 9.30E+01 |
| 4.00E+03 | 9.30E+01 |
| 4.00E+03 | 9.31E+01 |
| 4.00E+03 | 9.31E+01 |
|  |  |

| 399.2123 | 55.69093 |
| --- | --- |
| 401.1409 | 58.61115 |
| 403.0695 | 60.9384 |
| 404.998 | 60.93197 |
| 406.9266 | 60.81819 |
| 408.8552 | 60.9269 |
| 410.7837 | 60.93993 |
| 412.7123 | 60.86101 |
| 414.6408 | 60.74662 |
| 416.5694 | 60.6117 |
| 418.498 | 60.19772 |
| 420.4265 | 59.86655 |
| 422.3551 | 59.65335 |
| 424.2837 | 59.45135 |
| 426.2122 | 59.19275 |
| 428.1408 | 58.87728 |
| 430.0693 | 58.65445 |
| 431.9979 | 58.51928 |
| 433.9265 | 58.44729 |
| 435.855 | 58.4216 |
| 437.7836 | 58.41891 |
| 439.7121 | 58.43934 |
| 441.6407 | 58.47368 |
| 443.5693 | 58.51537 |
| 445.4978 | 58.55285 |
| 447.4264 | 58.56973 |
| 449.3549 | 58.53193 |
| 451.2835 | 58.43233 |
| 453.2121 | 58.25541 |
| 455.1406 | 58.02645 |
| 457.0692 | 57.77032 |
| 458.9978 | 57.50299 |
| 460.9263 | 57.25464 |
| 462.8549 | 57.05133 |
| 464.7834 | 56.91494 |
| 466.712 | 56.85968 |
| 468.6406 | 56.88195 |
| 470.5691 | 57.00282 |
| 472.4977 | 57.2222 |
| 474.4263 | 57.5457 |
| 476.3548 | 57.97552 |
| 478.2834 | 58.51316 |
| 480.2119 | 59.14426 |
| 482.1405 | 59.85115 |
| 484.0691 | 60.61384 |
| 485.9976 | 61.42931 |
| 487.9262 | 62.27827 |
| 489.8548 | 63.13962 |
| 491.7833 | 63.98651 |
| 493.7119 | 64.82065 |
| 495.6404 | 65.64009 |
| 497.569 | 66.43417 |
| 499.4976 | 67.19015 |
| 501.4261 | 67.89875 |
| 503.3547 | 68.55498 |
| 505.2832 | 69.16894 |
| 507.2118 | 69.73394 |
| 509.1404 | 70.26398 |
| 511.0689 | 70.76148 |
| 512.9975 | 71.22053 |
| 514.9261 | 71.63504 |
| 516.8546 | 72.00742 |
| 518.7832 | 72.33273 |
| 520.7117 | 72.61543 |
| 522.6403 | 72.84715 |
| 524.5688 | 73.01743 |
| 526.4974 | 73.11706 |
| 528.426 | 73.1586 |
| 530.3546 | 73.14632 |
| 532.2831 | 73.0784 |
| 534.2117 | 72.94857 |
| 536.1403 | 72.76406 |
| 538.0688 | 72.53175 |
| 539.9974 | 72.26018 |
| 541.9259 | 71.94869 |
| 543.8545 | 71.6152 |
| 545.783 | 71.26528 |
| 547.7116 | 70.90443 |
| 549.6402 | 70.54387 |
| 551.5687 | 70.2 |
| 553.4973 | 69.87699 |
| 555.4258 | 69.58066 |
| 557.3544 | 69.31129 |
| 559.283 | 69.07808 |
| 561.2115 | 68.88806 |
| 563.1401 | 68.75135 |
| 565.0687 | 68.66686 |
| 566.9973 | 68.63142 |
| 568.9258 | 68.63795 |
| 570.8544 | 68.68321 |
| 572.7829 | 68.76048 |
| 574.7115 | 68.867 |
| 576.64 | 68.99319 |
| 578.5686 | 69.14417 |
| 580.4972 | 69.31719 |
| 582.4257 | 69.50776 |
| 584.3543 | 69.71275 |
| 586.2828 | 69.92859 |
| 588.2114 | 70.14748 |
| 590.14 | 70.36671 |
| 592.0685 | 70.5805 |
| 593.9971 | 70.794 |
| 595.9257 | 71.00732 |
| 597.8542 | 71.21886 |
| 599.7828 | 71.42265 |
| 601.7114 | 71.62041 |
| 603.6399 | 71.81179 |
| 605.5685 | 71.9963 |
| 607.497 | 72.17507 |
| 609.4256 | 72.35426 |
| 611.3541 | 72.5363 |
| 613.2827 | 72.73002 |
| 615.2113 | 72.935 |
| 617.1398 | 73.15133 |
| 619.0684 | 73.37977 |
| 620.9969 | 73.61924 |
| 622.9255 | 73.86902 |
| 624.8541 | 74.13006 |
| 626.7827 | 74.39761 |
| 628.7112 | 74.67075 |
| 630.6398 | 74.94939 |
| 632.5684 | 75.22263 |
| 634.4969 | 75.46427 |
| 636.4255 | 75.70277 |
| 638.354 | 75.94089 |
| 640.2826 | 76.1743 |
| 642.2111 | 76.39736 |
| 644.1397 | 76.604 |
| 646.0682 | 76.78736 |
| 647.9968 | 76.94746 |
| 649.9254 | 77.08221 |
| 651.8539 | 77.19451 |
| 653.7825 | 77.28493 |
| 655.7111 | 77.35895 |
| 657.6396 | 77.42344 |
| 659.5682 | 77.4878 |
| 661.4968 | 77.55865 |
| 663.4254 | 77.6422 |
| 665.3539 | 77.74387 |
| 667.2825 | 77.85397 |
| 669.211 | 77.98172 |
| 671.1396 | 78.19652 |
| 673.0681 | 78.46897 |
| 674.9967 | 78.78323 |
| 676.9252 | 79.12608 |
| 678.8538 | 79.48682 |
| 680.7824 | 79.85669 |
| 682.7109 | 80.23277 |
| 684.6395 | 80.60542 |
| 686.5681 | 80.96635 |
| 688.4966 | 81.30486 |
| 690.4252 | 81.61993 |
| 692.3538 | 81.9095 |
| 694.2823 | 82.1731 |
| 696.2109 | 82.40825 |
| 698.1395 | 82.61864 |
| 700.068 | 82.80772 |
| 701.9966 | 82.981 |
| 703.9251 | 83.15537 |
| 705.8537 | 83.35227 |
| 707.7822 | 83.5472 |
| 709.7108 | 83.75076 |
| 711.6393 | 83.96988 |
| 713.5679 | 84.21484 |
| 715.4965 | 84.48916 |
| 717.425 | 84.79356 |
| 719.3536 | 85.12207 |
| 721.2822 | 85.47266 |
| 723.2108 | 85.83858 |
| 725.1393 | 86.21571 |
| 727.0679 | 86.59657 |
| 728.9965 | 86.97469 |
| 730.925 | 87.3409 |
| 732.8536 | 87.69097 |
| 734.7821 | 88.01712 |
| 736.7107 | 88.31856 |
| 738.6392 | 88.59422 |
| 740.5678 | 88.85126 |
| 742.4963 | 89.09296 |
| 744.4249 | 89.32344 |
| 746.3535 | 89.54421 |
| 748.282 | 89.76077 |
| 750.2106 | 89.97287 |
| 752.1392 | 90.17923 |
| 754.0677 | 90.37399 |
| 755.9963 | 90.55417 |
| 757.9249 | 90.71252 |
| 759.8534 | 90.84595 |
| 761.782 | 90.95009 |
| 763.7106 | 91.02516 |
| 765.6391 | 91.06955 |
| 767.5677 | 91.08755 |
| 769.4962 | 91.08501 |
| 771.4248 | 91.07506 |
| 773.3533 | 91.06428 |
| 775.2819 | 91.06071 |
| 777.2104 | 91.06988 |
| 779.139 | 91.10121 |
| 781.0676 | 91.15922 |
| 782.9962 | 91.24866 |
| 784.9247 | 91.36639 |
| 786.8533 | 91.50372 |
| 788.7819 | 91.64571 |
| 790.7104 | 91.78351 |
| 792.639 | 91.90833 |
| 794.5676 | 92.01285 |
| 796.4961 | 92.08743 |
| 798.4247 | 92.13109 |
| 800.3532 | 92.13948 |
| 802.2818 | 92.11406 |
| 804.2103 | 92.05843 |
| 806.1389 | 91.98137 |
| 808.0674 | 91.88924 |
| 809.996 | 91.79621 |
| 811.9246 | 91.718 |
| 813.8531 | 91.67471 |
| 815.7817 | 91.68041 |
| 817.7103 | 91.74767 |
| 819.6389 | 91.88461 |
| 821.5674 | 92.09975 |
| 823.496 | 92.39574 |
| 825.4245 | 92.77596 |
| 827.3531 | 93.2336 |
| 829.2817 | 93.75598 |
| 831.2102 | 94.32607 |
| 833.1388 | 94.9283 |
| 835.0673 | 95.53844 |
| 836.9959 | 96.13935 |
| 838.9244 | 96.71966 |
| 840.853 | 97.27512 |
| 842.7816 | 97.79948 |
| 844.7101 | 98.28831 |
| 846.6387 | 98.73027 |
| 848.5673 | 99.10982 |
| 850.4958 | 99.40216 |
| 852.4244 | 99.58652 |
| 854.353 | 99.64954 |
| 856.2815 | 99.58552 |
| 858.2101 | 99.38841 |
| 860.1387 | 99.06145 |
| 862.0672 | 98.61214 |
| 863.9958 | 98.05769 |
| 865.9243 | 97.42126 |
| 867.8529 | 96.73667 |
| 869.7814 | 96.04333 |
| 871.71 | 95.39495 |
| 873.6385 | 94.84772 |
| 875.5671 | 94.44881 |
| 877.4957 | 94.22134 |
| 879.4243 | 94.16858 |
| 881.3528 | 94.26929 |
| 883.2814 | 94.48629 |
| 885.21 | 94.77288 |
| 887.1385 | 95.08932 |
| 889.0671 | 95.40027 |
| 890.9956 | 95.67671 |
| 892.9242 | 95.89328 |
| 894.8528 | 96.0358 |
| 896.7813 | 96.09647 |
| 898.7099 | 96.07465 |
| 900.6384 | 95.97336 |
| 902.567 | 95.8074 |
| 904.4955 | 95.5951 |
| 906.4241 | 95.35711 |
| 908.3527 | 95.10549 |
| 910.2813 | 94.84354 |
| 912.2098 | 94.56236 |
| 914.1384 | 94.24955 |
| 916.067 | 93.88834 |
| 917.9955 | 93.46608 |
| 919.9241 | 92.97414 |
| 921.8526 | 92.41346 |
| 923.7812 | 91.78918 |
| 925.7097 | 91.11317 |
| 927.6383 | 90.39654 |
| 929.5669 | 89.65301 |
| 931.4954 | 88.89597 |
| 933.424 | 88.13752 |
| 935.3525 | 87.38455 |
| 937.2811 | 86.64489 |
| 939.2097 | 85.9204 |
| 941.1382 | 85.2107 |
| 943.0668 | 84.50995 |
| 944.9954 | 83.81313 |
| 946.924 | 83.11343 |
| 948.8525 | 82.40583 |
| 950.7811 | 81.68253 |
| 952.7096 | 80.93846 |
| 954.6382 | 80.16807 |
| 956.5667 | 79.3682 |
| 958.4953 | 78.53423 |
| 960.4239 | 77.66481 |
| 962.3524 | 76.7563 |
| 964.281 | 75.80716 |
| 966.2095 | 74.81423 |
| 968.1381 | 73.77586 |
| 970.0667 | 72.69023 |
| 971.9952 | 71.55955 |
| 973.9238 | 70.38782 |
| 975.8524 | 69.18484 |
| 977.7809 | 67.96007 |
| 979.7095 | 66.72713 |
| 981.6381 | 65.50001 |
| 983.5666 | 64.29474 |
| 985.4952 | 63.12339 |
| 987.4237 | 61.99891 |
| 989.3523 | 60.92956 |
| 991.2808 | 59.92054 |
| 993.2094 | 58.97067 |
| 995.138 | 58.07716 |
| 997.0665 | 57.23077 |
| 998.9951 | 56.42181 |
| 1000.924 | 55.63797 |
| 1002.852 | 54.86964 |
| 1004.781 | 54.10562 |
| 1006.709 | 53.33872 |
| 1008.638 | 52.56291 |
| 1010.566 | 51.77694 |
| 1012.495 | 50.98093 |
| 1014.424 | 50.18056 |
| 1016.352 | 49.38348 |
| 1018.281 | 48.60326 |
| 1020.209 | 47.85332 |
| 1022.138 | 47.15025 |
| 1024.066 | 46.50561 |
| 1025.995 | 45.92985 |
| 1027.924 | 45.42798 |
| 1029.852 | 45.00397 |
| 1031.781 | 44.65636 |
| 1033.709 | 44.38283 |
| 1035.638 | 44.17599 |
| 1037.566 | 44.02702 |
| 1039.495 | 43.92163 |
| 1041.423 | 43.84725 |
| 1043.352 | 43.78826 |
| 1045.281 | 43.7341 |
| 1047.209 | 43.67616 |
| 1049.138 | 43.61033 |
| 1051.066 | 43.53304 |
| 1052.995 | 43.44754 |
| 1054.923 | 43.35699 |
| 1056.852 | 43.26536 |
| 1058.781 | 43.17249 |
| 1060.709 | 43.08036 |
| 1062.638 | 42.98755 |
| 1064.566 | 42.89298 |
| 1066.495 | 42.7926 |
| 1068.423 | 42.68453 |
| 1070.352 | 42.56598 |
| 1072.28 | 42.4368 |
| 1074.209 | 42.29473 |
| 1076.138 | 42.14302 |
| 1078.066 | 41.98598 |
| 1079.995 | 41.83274 |
| 1081.923 | 41.69106 |
| 1083.852 | 41.5713 |
| 1085.78 | 41.48137 |
| 1087.709 | 41.43412 |
| 1089.637 | 41.43982 |
| 1091.566 | 41.50727 |
| 1093.495 | 41.64075 |
| 1095.423 | 41.84527 |
| 1097.352 | 42.12131 |
| 1099.28 | 42.46753 |
| 1101.209 | 42.87879 |
| 1103.137 | 43.35007 |
| 1105.066 | 43.87357 |
| 1106.995 | 44.44252 |
| 1108.923 | 45.0476 |
| 1110.852 | 45.68361 |
| 1112.78 | 46.34317 |
| 1114.709 | 47.02008 |
| 1116.637 | 47.70712 |
| 1118.566 | 48.40048 |
| 1120.495 | 49.09529 |
| 1122.423 | 49.79258 |
| 1124.352 | 50.49125 |
| 1126.28 | 51.18955 |
| 1128.209 | 51.88346 |
| 1130.137 | 52.57197 |
| 1132.066 | 53.24902 |
| 1133.994 | 53.90592 |
| 1135.923 | 54.53091 |
| 1137.852 | 55.11429 |
| 1139.78 | 55.64583 |
| 1141.709 | 56.11915 |
| 1143.637 | 56.52864 |
| 1145.566 | 56.87351 |
| 1147.494 | 57.15431 |
| 1149.423 | 57.37888 |
| 1151.351 | 57.55694 |
| 1153.28 | 57.70319 |
| 1155.209 | 57.83438 |
| 1157.137 | 57.9716 |
| 1159.066 | 58.13317 |
| 1160.994 | 58.33917 |
| 1162.923 | 58.60722 |
| 1164.851 | 58.95316 |
| 1166.78 | 59.38294 |
| 1168.708 | 59.8979 |
| 1170.637 | 60.49268 |
| 1172.566 | 61.15863 |
| 1174.494 | 61.87959 |
| 1176.423 | 62.63723 |
| 1178.351 | 63.40887 |
| 1180.28 | 64.1729 |
| 1182.208 | 64.90722 |
| 1184.137 | 65.59439 |
| 1186.066 | 66.2164 |
| 1187.994 | 66.76157 |
| 1189.923 | 67.22233 |
| 1191.851 | 67.59759 |
| 1193.78 | 67.88994 |
| 1195.708 | 68.10983 |
| 1197.637 | 68.26835 |
| 1199.566 | 68.3801 |
| 1201.494 | 68.4577 |
| 1203.423 | 68.5159 |
| 1205.351 | 68.56577 |
| 1207.28 | 68.61726 |
| 1209.208 | 68.67537 |
| 1211.137 | 68.74426 |
| 1213.065 | 68.82401 |
| 1214.994 | 68.91548 |
| 1216.923 | 69.01588 |
| 1218.851 | 69.12248 |
| 1220.78 | 69.22912 |
| 1222.708 | 69.3327 |
| 1224.637 | 69.4284 |
| 1226.565 | 69.51413 |
| 1228.494 | 69.58875 |
| 1230.422 | 69.65331 |
| 1232.351 | 69.70641 |
| 1234.28 | 69.74951 |
| 1236.208 | 69.78223 |
| 1238.137 | 69.80656 |
| 1240.065 | 69.82166 |
| 1241.994 | 69.82813 |
| 1243.922 | 69.82521 |
| 1245.851 | 69.81418 |
| 1247.78 | 69.79581 |
| 1249.708 | 69.77472 |
| 1251.637 | 69.75256 |
| 1253.565 | 69.7313 |
| 1255.494 | 69.71207 |
| 1257.422 | 69.69962 |
| 1259.351 | 69.69653 |
| 1261.28 | 69.708 |
| 1263.208 | 69.73722 |
| 1265.137 | 69.78648 |
| 1267.065 | 69.85508 |
| 1268.994 | 69.94382 |
| 1270.922 | 70.05064 |
| 1272.851 | 70.17487 |
| 1274.779 | 70.31219 |
| 1276.708 | 70.45956 |
| 1278.637 | 70.61024 |
| 1280.565 | 70.76219 |
| 1282.494 | 70.91296 |
| 1284.422 | 71.05789 |
| 1286.351 | 71.18803 |
| 1288.279 | 71.29649 |
| 1290.208 | 71.37388 |
| 1292.136 | 71.41261 |
| 1294.065 | 71.40321 |
| 1295.994 | 71.34128 |
| 1297.922 | 71.22422 |
| 1299.851 | 71.05562 |
| 1301.779 | 70.83871 |
| 1303.708 | 70.58025 |
| 1305.636 | 70.28586 |
| 1307.565 | 69.9761 |
| 1309.494 | 69.66982 |
| 1311.422 | 69.38322 |
| 1313.351 | 69.1277 |
| 1315.279 | 68.91721 |
| 1317.208 | 68.75822 |
| 1319.136 | 68.65179 |
| 1321.065 | 68.5958 |
| 1322.994 | 68.58602 |
| 1324.922 | 68.60754 |
| 1326.851 | 68.6432 |
| 1328.779 | 68.67031 |
| 1330.708 | 68.69102 |
| 1332.636 | 68.70433 |
| 1334.565 | 68.70138 |
| 1336.493 | 68.67657 |
| 1338.422 | 68.6218 |
| 1340.351 | 68.52579 |
| 1342.279 | 68.41643 |
| 1344.208 | 68.29659 |
| 1346.136 | 68.15948 |
| 1348.065 | 67.9957 |
| 1349.993 | 67.80231 |
| 1351.922 | 67.57098 |
| 1353.85 | 67.29156 |
| 1355.779 | 66.96636 |
| 1357.708 | 66.59532 |
| 1359.636 | 66.15492 |
| 1361.565 | 65.6342 |
| 1363.493 | 65.04923 |
| 1365.422 | 64.4351 |
| 1367.35 | 63.81583 |
| 1369.279 | 63.19333 |
| 1371.208 | 62.57274 |
| 1373.136 | 61.95972 |
| 1375.065 | 61.36457 |
| 1376.993 | 60.82589 |
| 1378.922 | 60.33356 |
| 1380.85 | 59.87545 |
| 1382.779 | 59.43346 |
| 1384.708 | 58.97243 |
| 1386.636 | 58.48594 |
| 1388.565 | 57.97578 |
| 1390.493 | 57.43149 |
| 1392.422 | 56.8358 |
| 1394.35 | 56.14743 |
| 1396.279 | 55.37281 |
| 1398.207 | 54.54725 |
| 1400.136 | 53.69098 |
| 1402.065 | 52.80272 |
| 1403.993 | 51.8828 |
| 1405.922 | 50.96592 |
| 1407.85 | 50.08733 |
| 1409.779 | 49.26744 |
| 1411.707 | 48.52672 |
| 1413.636 | 47.85384 |
| 1415.564 | 47.25645 |
| 1417.493 | 46.72911 |
| 1419.422 | 46.27221 |
| 1421.35 | 45.93264 |
| 1423.279 | 45.67242 |
| 1425.207 | 45.49283 |
| 1427.136 | 45.42315 |
| 1429.064 | 45.43066 |
| 1430.993 | 45.50378 |
| 1432.922 | 45.6527 |
| 1434.85 | 45.85867 |
| 1436.779 | 46.07048 |
| 1438.707 | 46.29438 |
| 1440.636 | 46.55275 |
| 1442.564 | 46.8138 |
| 1444.493 | 47.06969 |
| 1446.422 | 47.3084 |
| 1448.35 | 47.51964 |
| 1450.279 | 47.73349 |
| 1452.207 | 47.9406 |
| 1454.136 | 48.14525 |
| 1456.064 | 48.34155 |
| 1457.993 | 48.54559 |
| 1459.921 | 48.89218 |
| 1461.85 | 49.38272 |
| 1463.779 | 49.96799 |
| 1465.707 | 50.65447 |
| 1467.636 | 51.47749 |
| 1469.564 | 52.41192 |
| 1471.493 | 53.35873 |
| 1473.421 | 54.26684 |
| 1475.35 | 55.19209 |
| 1477.278 | 56.10344 |
| 1479.207 | 57.00934 |
| 1481.136 | 57.86049 |
| 1483.064 | 58.60073 |
| 1484.993 | 59.22318 |
| 1486.921 | 59.64808 |
| 1488.85 | 59.89049 |
| 1490.778 | 59.99245 |
| 1492.707 | 60.02661 |
| 1494.635 | 60.02844 |
| 1496.564 | 59.94515 |
| 1498.493 | 59.79088 |
| 1500.421 | 59.60664 |
| 1502.35 | 59.43843 |
| 1504.278 | 59.21975 |
| 1506.207 | 58.84141 |
| 1508.135 | 58.40422 |
| 1510.064 | 58.09016 |
| 1511.993 | 57.88108 |
| 1513.921 | 57.78595 |
| 1515.85 | 57.73189 |
| 1517.778 | 57.69376 |
| 1519.707 | 57.67656 |
| 1521.635 | 57.6381 |
| 1523.564 | 57.6296 |
| 1525.493 | 57.5925 |
| 1527.421 | 57.58371 |
| 1529.35 | 57.61842 |
| 1531.278 | 57.67136 |
| 1533.207 | 57.72503 |
| 1535.135 | 57.75478 |
| 1537.064 | 57.766 |
| 1538.992 | 57.68541 |
| 1540.921 | 57.50412 |
| 1542.85 | 57.37444 |
| 1544.778 | 57.35685 |
| 1546.707 | 57.41178 |
| 1548.635 | 57.51648 |
| 1550.564 | 57.60549 |
| 1552.492 | 57.6836 |
| 1554.421 | 57.70494 |
| 1556.349 | 57.64615 |
| 1558.278 | 57.48333 |
| 1560.207 | 57.20147 |
| 1562.135 | 56.98443 |
| 1564.064 | 56.75947 |
| 1565.992 | 56.46302 |
| 1567.921 | 56.08086 |
| 1569.849 | 55.58394 |
| 1571.778 | 55.02009 |
| 1573.707 | 54.38068 |
| 1575.635 | 53.66414 |
| 1577.564 | 52.94443 |
| 1579.492 | 52.27168 |
| 1581.421 | 51.5969 |
| 1583.349 | 50.88606 |
| 1585.278 | 50.16026 |
| 1587.207 | 49.42713 |
| 1589.135 | 48.64443 |
| 1591.064 | 47.84627 |
| 1592.992 | 47.04159 |
| 1594.921 | 46.27403 |
| 1596.849 | 45.60011 |
| 1598.778 | 44.93377 |
| 1600.706 | 44.2369 |
| 1602.635 | 43.50056 |
| 1604.564 | 42.75748 |
| 1606.492 | 42.04705 |
| 1608.421 | 41.35764 |
| 1610.349 | 40.68301 |
| 1612.278 | 40.02638 |
| 1614.206 | 39.40058 |
| 1616.135 | 38.78802 |
| 1618.063 | 38.18441 |
| 1619.992 | 37.64159 |
| 1621.921 | 37.19072 |
| 1623.849 | 36.83072 |
| 1625.778 | 36.61831 |
| 1627.706 | 36.52604 |
| 1629.635 | 36.53369 |
| 1631.563 | 36.65984 |
| 1633.492 | 36.84802 |
| 1635.421 | 37.01289 |
| 1637.349 | 37.21472 |
| 1639.278 | 37.52443 |
| 1641.206 | 37.86775 |
| 1643.135 | 38.25356 |
| 1645.063 | 38.64556 |
| 1646.992 | 39.0028 |
| 1648.92 | 39.3521 |
| 1650.849 | 39.67863 |
| 1652.778 | 39.97351 |
| 1654.706 | 40.33991 |
| 1656.635 | 40.87283 |
| 1658.563 | 41.53849 |
| 1660.492 | 42.2967 |
| 1662.42 | 43.06355 |
| 1664.349 | 43.86109 |
| 1666.277 | 44.65021 |
| 1668.206 | 45.42023 |
| 1670.135 | 46.17118 |
| 1672.063 | 46.96524 |
| 1673.992 | 47.80285 |
| 1675.92 | 48.61412 |
| 1677.849 | 49.46397 |
| 1679.777 | 50.28362 |
| 1681.706 | 51.03692 |
| 1683.635 | 51.68518 |
| 1685.563 | 52.28069 |
| 1687.492 | 52.96546 |
| 1689.42 | 53.75296 |
| 1691.349 | 54.63531 |
| 1693.277 | 55.5331 |
| 1695.206 | 56.3505 |
| 1697.135 | 57.09734 |
| 1699.063 | 57.80315 |
| 1700.992 | 58.40149 |
| 1702.92 | 59.06743 |
| 1704.849 | 59.74022 |
| 1706.777 | 60.41703 |
| 1708.706 | 61.12817 |
| 1710.634 | 61.83781 |
| 1712.563 | 62.54212 |
| 1714.492 | 63.15956 |
| 1716.42 | 63.67099 |
| 1718.349 | 64.11559 |
| 1720.277 | 64.65139 |
| 1722.206 | 65.27717 |
| 1724.134 | 65.89613 |
| 1726.063 | 66.51363 |
| 1727.991 | 67.07646 |
| 1729.92 | 67.56685 |
| 1731.849 | 68.04756 |
| 1733.777 | 68.49364 |
| 1735.706 | 68.96799 |
| 1737.634 | 69.58333 |
| 1739.563 | 70.21773 |
| 1741.491 | 70.94524 |
| 1743.42 | 71.78964 |
| 1745.349 | 72.69859 |
| 1747.277 | 73.65253 |
| 1749.206 | 74.63416 |
| 1751.134 | 75.64906 |
| 1753.063 | 76.75228 |
| 1754.991 | 77.95279 |
| 1756.92 | 79.11942 |
| 1758.849 | 80.22263 |
| 1760.777 | 81.25792 |
| 1762.706 | 82.1932 |
| 1764.634 | 83.06889 |
| 1766.563 | 83.85411 |
| 1768.491 | 84.51894 |
| 1770.42 | 85.1264 |
| 1772.348 | 85.65711 |
| 1774.277 | 86.14085 |
| 1776.206 | 86.63528 |
| 1778.134 | 87.10817 |
| 1780.063 | 87.54735 |
| 1781.991 | 87.95947 |
| 1783.92 | 88.36234 |
| 1785.848 | 88.75928 |
| 1787.777 | 89.17374 |
| 1789.705 | 89.55496 |
| 1791.634 | 89.84956 |
| 1793.563 | 90.12081 |
| 1795.491 | 90.39201 |
| 1797.42 | 90.66302 |
| 1799.348 | 90.95335 |
| 1801.277 | 91.22848 |
| 1803.205 | 91.51289 |
| 1805.134 | 91.8294 |
| 1807.063 | 92.17311 |
| 1808.991 | 92.54128 |
| 1810.92 | 92.89626 |
| 1812.848 | 93.24723 |
| 1814.777 | 93.60732 |
| 1816.705 | 93.97131 |
| 1818.634 | 94.33386 |
| 1820.563 | 94.67426 |
| 1822.491 | 94.98176 |
| 1824.42 | 95.2197 |
| 1826.348 | 95.40195 |
| 1828.277 | 95.57519 |
| 1830.205 | 95.71859 |
| 1832.134 | 95.85763 |
| 1834.062 | 95.98812 |
| 1835.991 | 96.10088 |
| 1837.92 | 96.227 |
| 1839.848 | 96.36092 |
| 1841.777 | 96.49152 |
| 1843.705 | 96.58701 |
| 1845.634 | 96.6799 |
| 1847.562 | 96.82344 |
| 1849.491 | 96.99663 |
| 1851.419 | 97.19051 |
| 1853.348 | 97.39015 |
| 1855.277 | 97.5684 |
| 1857.205 | 97.72427 |
| 1859.134 | 97.86097 |
| 1861.062 | 97.98416 |
| 1862.991 | 98.09647 |
| 1864.919 | 98.18353 |
| 1866.848 | 98.2551 |
| 1868.776 | 98.28678 |
| 1870.705 | 98.3109 |
| 1872.634 | 98.35838 |
| 1874.562 | 98.40634 |
| 1876.491 | 98.44949 |
| 1878.419 | 98.48883 |
| 1880.348 | 98.5526 |
| 1882.276 | 98.64326 |
| 1884.205 | 98.72642 |
| 1886.134 | 98.81536 |
| 1888.062 | 98.89136 |
| 1889.991 | 98.94583 |
| 1891.919 | 99.01588 |
| 1893.848 | 99.09759 |
| 1895.776 | 99.175 |
| 1897.705 | 99.25324 |
| 1899.634 | 99.32388 |
| 1901.562 | 99.38558 |
| 1903.491 | 99.44501 |
| 1905.419 | 99.52033 |
| 1907.348 | 99.59187 |
| 1909.276 | 99.63689 |
| 1911.205 | 99.67327 |
| 1913.133 | 99.70658 |
| 1915.062 | 99.73597 |
| 1916.991 | 99.74931 |
| 1918.919 | 99.74116 |
| 1920.848 | 99.7392 |
| 1922.776 | 99.72705 |
| 1924.705 | 99.72191 |
| 1926.633 | 99.74477 |
| 1928.562 | 99.77293 |
| 1930.49 | 99.80371 |
| 1932.419 | 99.83636 |
| 1934.348 | 99.86555 |
| 1936.276 | 99.89426 |
| 1938.205 | 99.9157 |
| 1940.133 | 99.92589 |
| 1942.062 | 99.91062 |
| 1943.99 | 99.88223 |
| 1945.919 | 99.86188 |
| 1947.848 | 99.84235 |
| 1949.776 | 99.82129 |
| 1951.705 | 99.7936 |
| 1953.633 | 99.76307 |
| 1955.562 | 99.73976 |
| 1957.49 | 99.71124 |
| 1959.419 | 99.68948 |
| 1961.348 | 99.67583 |
| 1963.276 | 99.66177 |
| 1965.205 | 99.64925 |
| 1967.133 | 99.63234 |
| 1969.062 | 99.62002 |
| 1970.99 | 99.61517 |
| 1972.919 | 99.60838 |
| 1974.847 | 99.59844 |
| 1976.776 | 99.58349 |
| 1978.705 | 99.57275 |
| 1980.633 | 99.56674 |
| 1982.562 | 99.55405 |
| 1984.49 | 99.53485 |
| 1986.419 | 99.50684 |
| 1988.347 | 99.4677 |
| 1990.276 | 99.42066 |
| 1992.204 | 99.36279 |
| 1994.133 | 99.30523 |
| 1996.062 | 99.25225 |
| 1997.99 | 99.1983 |
| 1999.919 | 99.14536 |
| 2001.847 | 99.09277 |
| 2003.776 | 99.04509 |
| 2005.704 | 98.99863 |
| 2007.633 | 98.94775 |
| 2009.562 | 98.89466 |
| 2011.49 | 98.83961 |
| 2013.419 | 98.78112 |
| 2015.347 | 98.71658 |
| 2017.276 | 98.64232 |
| 2019.204 | 98.56532 |
| 2021.133 | 98.48846 |
| 2023.062 | 98.41043 |
| 2024.99 | 98.33365 |
| 2026.919 | 98.25847 |
| 2028.847 | 98.19025 |
| 2030.776 | 98.12489 |
| 2032.704 | 98.06094 |
| 2034.633 | 98.00167 |
| 2036.561 | 97.9445 |
| 2038.49 | 97.8877 |
| 2040.419 | 97.82852 |
| 2042.347 | 97.76682 |
| 2044.276 | 97.70599 |
| 2046.204 | 97.64476 |
| 2048.133 | 97.58136 |
| 2050.061 | 97.51579 |
| 2051.99 | 97.44914 |
| 2053.918 | 97.38595 |
| 2055.847 | 97.32504 |
| 2057.776 | 97.26522 |
| 2059.704 | 97.207 |
| 2061.633 | 97.14998 |
| 2063.561 | 97.09425 |
| 2065.49 | 97.03958 |
| 2067.418 | 96.99047 |
| 2069.347 | 96.94619 |
| 2071.276 | 96.90361 |
| 2073.204 | 96.86072 |
| 2075.133 | 96.81638 |
| 2077.061 | 96.77216 |
| 2078.99 | 96.72916 |
| 2080.918 | 96.68481 |
| 2082.847 | 96.63824 |
| 2084.775 | 96.58929 |
| 2086.704 | 96.53793 |
| 2088.633 | 96.48454 |
| 2090.561 | 96.42909 |
| 2092.49 | 96.37287 |
| 2094.418 | 96.31597 |
| 2096.347 | 96.25819 |
| 2098.275 | 96.20017 |
| 2100.204 | 96.14211 |
| 2102.133 | 96.08549 |
| 2104.061 | 96.02838 |
| 2105.99 | 95.97037 |
| 2107.918 | 95.91116 |
| 2109.847 | 95.85104 |
| 2111.775 | 95.79059 |
| 2113.704 | 95.72937 |
| 2115.633 | 95.66655 |
| 2117.561 | 95.60368 |
| 2119.49 | 95.54199 |
| 2121.418 | 95.48279 |
| 2123.347 | 95.42624 |
| 2125.275 | 95.37317 |
| 2127.204 | 95.32469 |
| 2129.132 | 95.28079 |
| 2131.061 | 95.24126 |
| 2132.99 | 95.20605 |
| 2134.918 | 95.17455 |
| 2136.847 | 95.14619 |
| 2138.775 | 95.12087 |
| 2140.704 | 95.09864 |
| 2142.632 | 95.07832 |
| 2144.561 | 95.05894 |
| 2146.49 | 95.03955 |
| 2148.418 | 95.01991 |
| 2150.347 | 95.00098 |
| 2152.275 | 94.98397 |
| 2154.204 | 94.96857 |
| 2156.132 | 94.95447 |
| 2158.061 | 94.94139 |
| 2159.989 | 94.93077 |
| 2161.918 | 94.92341 |
| 2163.846 | 94.9191 |
| 2165.775 | 94.91685 |
| 2167.704 | 94.91665 |
| 2169.632 | 94.91799 |
| 2171.561 | 94.91995 |
| 2173.489 | 94.92269 |
| 2175.418 | 94.92641 |
| 2177.346 | 94.9305 |
| 2179.275 | 94.93494 |
| 2181.204 | 94.93945 |
| 2183.132 | 94.94463 |
| 2185.061 | 94.95142 |
| 2186.989 | 94.95961 |
| 2188.918 | 94.9678 |
| 2190.846 | 94.97571 |
| 2192.775 | 94.9842 |
| 2194.704 | 94.99422 |
| 2196.632 | 95.00471 |
| 2198.561 | 95.01459 |
| 2200.489 | 95.02336 |
| 2202.418 | 95.03116 |
| 2204.346 | 95.0369 |
| 2206.275 | 95.04028 |
| 2208.203 | 95.04204 |
| 2210.132 | 95.04317 |
| 2212.061 | 95.04382 |
| 2213.989 | 95.0444 |
| 2215.918 | 95.04456 |
| 2217.846 | 95.04505 |
| 2219.775 | 95.04604 |
| 2221.703 | 95.04672 |
| 2223.632 | 95.04613 |
| 2225.561 | 95.04467 |
| 2227.489 | 95.04266 |
| 2229.418 | 95.04028 |
| 2231.346 | 95.03668 |
| 2233.275 | 95.03222 |
| 2235.203 | 95.02756 |
| 2237.132 | 95.02321 |
| 2239.06 | 95.01928 |
| 2240.989 | 95.01661 |
| 2242.917 | 95.01524 |
| 2244.846 | 95.01551 |
| 2246.775 | 95.01733 |
| 2248.703 | 95.021 |
| 2250.632 | 95.02578 |
| 2252.56 | 95.03135 |
| 2254.489 | 95.03613 |
| 2256.417 | 95.03872 |
| 2258.346 | 95.03809 |
| 2260.275 | 95.03399 |
| 2262.203 | 95.02608 |
| 2264.132 | 95.0146 |
| 2266.06 | 94.99981 |
| 2267.989 | 94.98362 |
| 2269.917 | 94.96715 |
| 2271.846 | 94.95052 |
| 2273.774 | 94.93275 |
| 2275.703 | 94.91317 |
| 2277.632 | 94.89069 |
| 2279.56 | 94.86752 |
| 2281.489 | 94.84652 |
| 2283.417 | 94.82778 |
| 2285.346 | 94.8068 |
| 2287.274 | 94.78098 |
| 2289.203 | 94.74951 |
| 2291.132 | 94.71768 |
| 2293.06 | 94.68935 |
| 2294.989 | 94.66369 |
| 2296.917 | 94.63886 |
| 2298.846 | 94.62005 |
| 2300.774 | 94.61263 |
| 2302.703 | 94.61896 |
| 2304.632 | 94.63453 |
| 2306.56 | 94.64827 |
| 2308.489 | 94.65052 |
| 2310.417 | 94.63581 |
| 2312.346 | 94.61456 |
| 2314.274 | 94.60718 |
| 2316.203 | 94.59176 |
| 2318.131 | 94.52301 |
| 2320.06 | 94.36919 |
| 2321.989 | 94.11919 |
| 2323.917 | 93.78475 |
| 2325.846 | 93.39535 |
| 2327.774 | 92.97659 |
| 2329.703 | 92.55137 |
| 2331.631 | 92.15565 |
| 2333.56 | 91.83515 |
| 2335.489 | 91.62195 |
| 2337.417 | 91.53233 |
| 2339.346 | 91.5687 |
| 2341.274 | 91.72741 |
| 2343.203 | 92.00914 |
| 2345.131 | 92.41062 |
| 2347.06 | 92.92353 |
| 2348.989 | 93.49004 |
| 2350.917 | 93.97785 |
| 2352.845 | 94.28957 |
| 2354.774 | 94.38853 |
| 2356.703 | 94.26823 |
| 2358.631 | 93.95444 |
| 2360.56 | 93.48486 |
| 2362.488 | 92.89729 |
| 2364.417 | 92.2397 |
| 2366.345 | 91.5788 |
| 2368.274 | 90.97982 |
| 2370.203 | 90.49307 |
| 2372.131 | 90.15606 |
| 2374.06 | 89.99668 |
| 2375.988 | 90.034 |
| 2377.917 | 90.27144 |
| 2379.845 | 90.68732 |
| 2381.774 | 91.23428 |
| 2383.703 | 91.83283 |
| 2385.631 | 92.40018 |
| 2387.56 | 92.89852 |
| 2389.488 | 93.31008 |
| 2391.417 | 93.62133 |
| 2393.345 | 93.83006 |
| 2395.274 | 93.93556 |
| 2397.202 | 93.94267 |
| 2399.131 | 93.86639 |
| 2401.06 | 93.73006 |
| 2402.988 | 93.55532 |
| 2404.917 | 93.36138 |
| 2406.845 | 93.16414 |
| 2408.774 | 92.97783 |
| 2410.702 | 92.81255 |
| 2412.631 | 92.67127 |
| 2414.56 | 92.55294 |
| 2416.488 | 92.45441 |
| 2418.417 | 92.3706 |
| 2420.345 | 92.29573 |
| 2422.274 | 92.22541 |
| 2424.202 | 92.15665 |
| 2426.131 | 92.08707 |
| 2428.06 | 92.01453 |
| 2429.988 | 91.93726 |
| 2431.917 | 91.85475 |
| 2433.845 | 91.76743 |
| 2435.774 | 91.67664 |
| 2437.702 | 91.58348 |
| 2439.631 | 91.48852 |
| 2441.559 | 91.39198 |
| 2443.488 | 91.29461 |
| 2445.417 | 91.19652 |
| 2447.345 | 91.09712 |
| 2449.274 | 90.99677 |
| 2451.202 | 90.89549 |
| 2453.131 | 90.79295 |
| 2455.059 | 90.68822 |
| 2456.988 | 90.58061 |
| 2458.917 | 90.46951 |
| 2460.845 | 90.35491 |
| 2462.774 | 90.23701 |
| 2464.702 | 90.11655 |
| 2466.631 | 89.99416 |
| 2468.559 | 89.87038 |
| 2470.488 | 89.74592 |
| 2472.417 | 89.6204 |
| 2474.345 | 89.49335 |
| 2476.273 | 89.36447 |
| 2478.202 | 89.23328 |
| 2480.131 | 89.09927 |
| 2482.059 | 88.9619 |
| 2483.988 | 88.82086 |
| 2485.916 | 88.67549 |
| 2487.845 | 88.52595 |
| 2489.773 | 88.37143 |
| 2491.702 | 88.21155 |
| 2493.631 | 88.04635 |
| 2495.559 | 87.87698 |
| 2497.488 | 87.70512 |
| 2499.416 | 87.53278 |
| 2501.345 | 87.36125 |
| 2503.273 | 87.19159 |
| 2505.202 | 87.0247 |
| 2507.131 | 86.8616 |
| 2509.059 | 86.7034 |
| 2510.988 | 86.5506 |
| 2512.916 | 86.40423 |
| 2514.845 | 86.26516 |
| 2516.773 | 86.1331 |
| 2518.702 | 86.00788 |
| 2520.63 | 85.88879 |
| 2522.559 | 85.7755 |
| 2524.488 | 85.66644 |
| 2526.416 | 85.56059 |
| 2528.345 | 85.45728 |
| 2530.273 | 85.35642 |
| 2532.202 | 85.25771 |
| 2534.13 | 85.16024 |
| 2536.059 | 85.06249 |
| 2537.988 | 84.96345 |
| 2539.916 | 84.86275 |
| 2541.845 | 84.76038 |
| 2543.773 | 84.65601 |
| 2545.702 | 84.55003 |
| 2547.63 | 84.44353 |
| 2549.559 | 84.33651 |
| 2551.488 | 84.22829 |
| 2553.416 | 84.11915 |
| 2555.344 | 84.00906 |
| 2557.273 | 83.89922 |
| 2559.202 | 83.79035 |
| 2561.13 | 83.68285 |
| 2563.059 | 83.57658 |
| 2564.987 | 83.4717 |
| 2566.916 | 83.36752 |
| 2568.844 | 83.26269 |
| 2570.773 | 83.15616 |
| 2572.702 | 83.04778 |
| 2574.63 | 82.93769 |
| 2576.559 | 82.82533 |
| 2578.487 | 82.71024 |
| 2580.416 | 82.5927 |
| 2582.344 | 82.47205 |
| 2584.273 | 82.34823 |
| 2586.202 | 82.22107 |
| 2588.13 | 82.09116 |
| 2590.059 | 81.95956 |
| 2591.987 | 81.82835 |
| 2593.916 | 81.69828 |
| 2595.844 | 81.56879 |
| 2597.773 | 81.43906 |
| 2599.701 | 81.30972 |
| 2601.63 | 81.18027 |
| 2603.559 | 81.05013 |
| 2605.487 | 80.91947 |
| 2607.416 | 80.78868 |
| 2609.344 | 80.6578 |
| 2611.273 | 80.52638 |
| 2613.201 | 80.39435 |
| 2615.13 | 80.26221 |
| 2617.059 | 80.12975 |
| 2618.987 | 79.99783 |
| 2620.916 | 79.86567 |
| 2622.844 | 79.73305 |
| 2624.773 | 79.60069 |
| 2626.701 | 79.46861 |
| 2628.63 | 79.33515 |
| 2630.559 | 79.19893 |
| 2632.487 | 79.05936 |
| 2634.416 | 78.91707 |
| 2636.344 | 78.7711 |
| 2638.273 | 78.62149 |
| 2640.201 | 78.46894 |
| 2642.13 | 78.31389 |
| 2644.058 | 78.15691 |
| 2645.987 | 77.99932 |
| 2647.916 | 77.84277 |
| 2649.844 | 77.68852 |
| 2651.773 | 77.53621 |
| 2653.701 | 77.38572 |
| 2655.63 | 77.23631 |
| 2657.558 | 77.08845 |
| 2659.487 | 76.94267 |
| 2661.416 | 76.79784 |
| 2663.344 | 76.65286 |
| 2665.273 | 76.50805 |
| 2667.201 | 76.36316 |
| 2669.13 | 76.21792 |
| 2671.058 | 76.0713 |
| 2672.987 | 75.92396 |
| 2674.916 | 75.77644 |
| 2676.844 | 75.62888 |
| 2678.772 | 75.48193 |
| 2680.701 | 75.33732 |
| 2682.63 | 75.19556 |
| 2684.558 | 75.05579 |
| 2686.487 | 74.91724 |
| 2688.415 | 74.78027 |
| 2690.344 | 74.64482 |
| 2692.272 | 74.51139 |
| 2694.201 | 74.37994 |
| 2696.13 | 74.24914 |
| 2698.058 | 74.11848 |
| 2699.987 | 73.9882 |
| 2701.915 | 73.85735 |
| 2703.844 | 73.72509 |
| 2705.772 | 73.59138 |
| 2707.701 | 73.45771 |
| 2709.63 | 73.32436 |
| 2711.558 | 73.19115 |
| 2713.487 | 73.05887 |
| 2715.415 | 72.92909 |
| 2717.344 | 72.80158 |
| 2719.272 | 72.67698 |
| 2721.201 | 72.55676 |
| 2723.129 | 72.44201 |
| 2725.058 | 72.33291 |
| 2726.987 | 72.23003 |
| 2728.915 | 72.13309 |
| 2730.844 | 72.04066 |
| 2732.772 | 71.95239 |
| 2734.701 | 71.86858 |
| 2736.629 | 71.78801 |
| 2738.558 | 71.7097 |
| 2740.487 | 71.63354 |
| 2742.415 | 71.55962 |
| 2744.344 | 71.4854 |
| 2746.272 | 71.40895 |
| 2748.201 | 71.33041 |
| 2750.129 | 71.24982 |
| 2752.058 | 71.16622 |
| 2753.987 | 71.08073 |
| 2755.915 | 70.99317 |
| 2757.844 | 70.90287 |
| 2759.772 | 70.80902 |
| 2761.701 | 70.71143 |
| 2763.629 | 70.60973 |
| 2765.558 | 70.50338 |
| 2767.486 | 70.39262 |
| 2769.415 | 70.27844 |
| 2771.344 | 70.16045 |
| 2773.272 | 70.03819 |
| 2775.201 | 69.91138 |
| 2777.129 | 69.77965 |
| 2779.058 | 69.64132 |
| 2780.986 | 69.49686 |
| 2782.915 | 69.34765 |
| 2784.844 | 69.19366 |
| 2786.772 | 69.03478 |
| 2788.701 | 68.87206 |
| 2790.629 | 68.70428 |
| 2792.558 | 68.53071 |
| 2794.486 | 68.3507 |
| 2796.415 | 68.16431 |
| 2798.343 | 67.9718 |
| 2800.272 | 67.77307 |
| 2802.2 | 67.56829 |
| 2804.129 | 67.35928 |
| 2806.058 | 67.14543 |
| 2807.986 | 66.92537 |
| 2809.915 | 66.69866 |
| 2811.843 | 66.46516 |
| 2813.772 | 66.22526 |
| 2815.7 | 65.98081 |
| 2817.629 | 65.73174 |
| 2819.558 | 65.47385 |
| 2821.486 | 65.19643 |
| 2823.415 | 64.8806 |
| 2825.343 | 64.4991 |
| 2827.272 | 64.02257 |
| 2829.2 | 63.42327 |
| 2831.129 | 62.67968 |
| 2833.057 | 61.77959 |
| 2834.986 | 60.72229 |
| 2836.915 | 59.52118 |
| 2838.843 | 58.20357 |
| 2840.772 | 56.80418 |
| 2842.7 | 55.36672 |
| 2844.629 | 53.94371 |
| 2846.557 | 52.5924 |
| 2848.486 | 51.37285 |
| 2850.415 | 50.3418 |
| 2852.343 | 49.5428 |
| 2854.272 | 48.9998 |
| 2856.2 | 48.71276 |
| 2858.129 | 48.65936 |
| 2860.057 | 48.8004 |
| 2861.986 | 49.08875 |
| 2863.915 | 49.47463 |
| 2865.843 | 49.9099 |
| 2867.771 | 50.35171 |
| 2869.7 | 50.76446 |
| 2871.629 | 51.12019 |
| 2873.557 | 51.39647 |
| 2875.486 | 51.57344 |
| 2877.414 | 51.63848 |
| 2879.343 | 51.58931 |
| 2881.271 | 51.43328 |
| 2883.2 | 51.18582 |
| 2885.129 | 50.86427 |
| 2887.057 | 50.48189 |
| 2888.986 | 50.04654 |
| 2890.914 | 49.55835 |
| 2892.843 | 49.01028 |
| 2894.771 | 48.39001 |
| 2896.7 | 47.68423 |
| 2898.629 | 46.87902 |
| 2900.557 | 45.96261 |
| 2902.486 | 44.92767 |
| 2904.414 | 43.77327 |
| 2906.343 | 42.50595 |
| 2908.271 | 41.1408 |
| 2910.2 | 39.70309 |
| 2912.128 | 38.23297 |
| 2914.057 | 36.78744 |
| 2915.986 | 35.43495 |
| 2917.914 | 34.24743 |
| 2919.843 | 33.29388 |
| 2921.771 | 32.63341 |
| 2923.7 | 32.30777 |
| 2925.628 | 32.33411 |
| 2927.557 | 32.70381 |
| 2929.486 | 33.38307 |
| 2931.414 | 34.31756 |
| 2933.343 | 35.43713 |
| 2935.271 | 36.66412 |
| 2937.2 | 37.91874 |
| 2939.128 | 39.12677 |
| 2941.057 | 40.22646 |
| 2942.986 | 41.17302 |
| 2944.914 | 41.94226 |
| 2946.843 | 42.53411 |
| 2948.771 | 42.97167 |
| 2950.7 | 43.29347 |
| 2952.628 | 43.54708 |
| 2954.557 | 43.7866 |
| 2956.485 | 44.0633 |
| 2958.414 | 44.41742 |
| 2960.343 | 44.87555 |
| 2962.271 | 45.45054 |
| 2964.2 | 46.13946 |
| 2966.128 | 46.92789 |
| 2968.057 | 47.79201 |
| 2969.985 | 48.70127 |
| 2971.914 | 49.62215 |
| 2973.843 | 50.52166 |
| 2975.771 | 51.3704 |
| 2977.7 | 52.14375 |
| 2979.628 | 52.82415 |
| 2981.557 | 53.40406 |
| 2983.485 | 53.88429 |
| 2985.414 | 54.2702 |
| 2987.343 | 54.56976 |
| 2989.271 | 54.79489 |
| 2991.199 | 54.95623 |
| 2993.128 | 55.06408 |
| 2995.057 | 55.12785 |
| 2996.985 | 55.15438 |
| 2998.914 | 55.15041 |
| 3000.842 | 55.12296 |
| 3002.771 | 55.07618 |
| 3004.699 | 55.01304 |
| 3006.628 | 54.93735 |
| 3008.557 | 54.85059 |
| 3010.485 | 54.75313 |
| 3012.414 | 54.6459 |
| 3014.342 | 54.53017 |
| 3016.271 | 54.40982 |
| 3018.199 | 54.28733 |
| 3020.128 | 54.1616 |
| 3022.057 | 54.02948 |
| 3023.985 | 53.89098 |
| 3025.914 | 53.74554 |
| 3027.842 | 53.59437 |
| 3029.771 | 53.43548 |
| 3031.699 | 53.26785 |
| 3033.628 | 53.09626 |
| 3035.556 | 52.92328 |
| 3037.485 | 52.74899 |
| 3039.414 | 52.57449 |
| 3041.342 | 52.3998 |
| 3043.271 | 52.22287 |
| 3045.199 | 52.04223 |
| 3047.128 | 51.85859 |
| 3049.056 | 51.67296 |
| 3050.985 | 51.48791 |
| 3052.914 | 51.30214 |
| 3054.842 | 51.1134 |
| 3056.771 | 50.92178 |
| 3058.699 | 50.73067 |
| 3060.628 | 50.53904 |
| 3062.556 | 50.34766 |
| 3064.485 | 50.15694 |
| 3066.414 | 49.96926 |
| 3068.342 | 49.7893 |
| 3070.271 | 49.61924 |
| 3072.199 | 49.45906 |
| 3074.128 | 49.30809 |
| 3076.056 | 49.16312 |
| 3077.985 | 49.02265 |
| 3079.913 | 48.88555 |
| 3081.842 | 48.7514 |
| 3083.771 | 48.6196 |
| 3085.699 | 48.48856 |
| 3087.628 | 48.35376 |
| 3089.556 | 48.21455 |
| 3091.485 | 48.07149 |
| 3093.413 | 47.92378 |
| 3095.342 | 47.76971 |
| 3097.271 | 47.61085 |
| 3099.199 | 47.4476 |
| 3101.128 | 47.28083 |
| 3103.056 | 47.11388 |
| 3104.985 | 46.94917 |
| 3106.913 | 46.78484 |
| 3108.842 | 46.61958 |
| 3110.771 | 46.45275 |
| 3112.699 | 46.28444 |
| 3114.627 | 46.11251 |
| 3116.556 | 45.93573 |
| 3118.485 | 45.75371 |
| 3120.413 | 45.56567 |
| 3122.342 | 45.37086 |
| 3124.27 | 45.17266 |
| 3126.199 | 44.97164 |
| 3128.127 | 44.76745 |
| 3130.056 | 44.56233 |
| 3131.985 | 44.35596 |
| 3133.913 | 44.14902 |
| 3135.842 | 43.94546 |
| 3137.77 | 43.74628 |
| 3139.699 | 43.55094 |
| 3141.627 | 43.3576 |
| 3143.556 | 43.16468 |
| 3145.485 | 42.97168 |
| 3147.413 | 42.77736 |
| 3149.342 | 42.57971 |
| 3151.27 | 42.37782 |
| 3153.199 | 42.1722 |
| 3155.127 | 41.96308 |
| 3157.056 | 41.75008 |
| 3158.984 | 41.53365 |
| 3160.913 | 41.31249 |
| 3162.842 | 41.08649 |
| 3164.77 | 40.85741 |
| 3166.699 | 40.62598 |
| 3168.627 | 40.39454 |
| 3170.556 | 40.16502 |
| 3172.484 | 39.93628 |
| 3174.413 | 39.70716 |
| 3176.342 | 39.47663 |
| 3178.27 | 39.24449 |
| 3180.199 | 39.01174 |
| 3182.127 | 38.77988 |
| 3184.056 | 38.54714 |
| 3185.984 | 38.31317 |
| 3187.913 | 38.08059 |
| 3189.842 | 37.84961 |
| 3191.77 | 37.61908 |
| 3193.698 | 37.38792 |
| 3195.627 | 37.15543 |
| 3197.556 | 36.92232 |
| 3199.484 | 36.69122 |
| 3201.413 | 36.46348 |
| 3203.341 | 36.23821 |
| 3205.27 | 36.01526 |
| 3207.198 | 35.79343 |
| 3209.127 | 35.56963 |
| 3211.056 | 35.34329 |
| 3212.984 | 35.11641 |
| 3214.913 | 34.88844 |
| 3216.841 | 34.66129 |
| 3218.77 | 34.43116 |
| 3220.698 | 34.19898 |
| 3222.627 | 33.96877 |
| 3224.556 | 33.7398 |
| 3226.484 | 33.51101 |
| 3228.413 | 33.28219 |
| 3230.341 | 33.05376 |
| 3232.27 | 32.82809 |
| 3234.198 | 32.60694 |
| 3236.127 | 32.38999 |
| 3238.055 | 32.17634 |
| 3239.984 | 31.96669 |
| 3241.913 | 31.76034 |
| 3243.841 | 31.55702 |
| 3245.77 | 31.35815 |
| 3247.698 | 31.16638 |
| 3249.627 | 30.97995 |
| 3251.555 | 30.79789 |
| 3253.484 | 30.61571 |
| 3255.413 | 30.43562 |
| 3257.341 | 30.25936 |
| 3259.27 | 30.08485 |
| 3261.198 | 29.91181 |
| 3263.127 | 29.73948 |
| 3265.055 | 29.56696 |
| 3266.984 | 29.3962 |
| 3268.913 | 29.22686 |
| 3270.841 | 29.05892 |
| 3272.77 | 28.8919 |
| 3274.698 | 28.72609 |
| 3276.627 | 28.56363 |
| 3278.555 | 28.40627 |
| 3280.484 | 28.25515 |
| 3282.412 | 28.11103 |
| 3284.341 | 27.97032 |
| 3286.27 | 27.83031 |
| 3288.198 | 27.68922 |
| 3290.127 | 27.54707 |
| 3292.055 | 27.4015 |
| 3293.984 | 27.25207 |
| 3295.912 | 27.09752 |
| 3297.841 | 26.93441 |
| 3299.77 | 26.7613 |
| 3301.698 | 26.57756 |
| 3303.627 | 26.38173 |
| 3305.555 | 26.17497 |
| 3307.484 | 25.95601 |
| 3309.412 | 25.72543 |
| 3311.341 | 25.48663 |
| 3313.27 | 25.24075 |
| 3315.198 | 24.98887 |
| 3317.126 | 24.73031 |
| 3319.055 | 24.46287 |
| 3320.984 | 24.18639 |
| 3322.912 | 23.90116 |
| 3324.841 | 23.60866 |
| 3326.769 | 23.30931 |
| 3328.698 | 23.00489 |
| 3330.626 | 22.69301 |
| 3332.555 | 22.37278 |
| 3334.484 | 22.04564 |
| 3336.412 | 21.71356 |
| 3338.341 | 21.38042 |
| 3340.269 | 21.04792 |
| 3342.198 | 20.71289 |
| 3344.126 | 20.37536 |
| 3346.055 | 20.03632 |
| 3347.984 | 19.69605 |
| 3349.912 | 19.35133 |
| 3351.841 | 19.00059 |
| 3353.769 | 18.64294 |
| 3355.698 | 18.2783 |
| 3357.626 | 17.90475 |
| 3359.555 | 17.52444 |
| 3361.483 | 17.13788 |
| 3363.412 | 16.74569 |
| 3365.341 | 16.35033 |
| 3367.269 | 15.95723 |
| 3369.198 | 15.57135 |
| 3371.126 | 15.19714 |
| 3373.055 | 14.83634 |
| 3374.983 | 14.489 |
| 3376.912 | 14.15517 |
| 3378.841 | 13.83755 |
| 3380.769 | 13.53775 |
| 3382.698 | 13.25741 |
| 3384.626 | 12.99144 |
| 3386.555 | 12.73788 |
| 3388.483 | 12.4968 |
| 3390.412 | 12.26756 |
| 3392.34 | 12.04614 |
| 3394.269 | 11.83595 |
| 3396.198 | 11.63618 |
| 3398.126 | 11.44511 |
| 3400.055 | 11.2651 |
| 3401.983 | 11.09552 |
| 3403.912 | 10.93597 |
| 3405.84 | 10.78739 |
| 3407.769 | 10.64534 |
| 3409.698 | 10.51475 |
| 3411.626 | 10.39583 |
| 3413.555 | 10.28929 |
| 3415.483 | 10.20289 |
| 3417.412 | 10.13797 |
| 3419.34 | 10.09036 |
| 3421.269 | 10.06098 |
| 3423.198 | 10.05509 |
| 3425.126 | 10.07202 |
| 3427.054 | 10.10688 |
| 3428.983 | 10.16125 |
| 3430.912 | 10.23333 |
| 3432.84 | 10.31958 |
| 3434.769 | 10.42043 |
| 3436.697 | 10.5332 |
| 3438.626 | 10.6569 |
| 3440.554 | 10.78908 |
| 3442.483 | 10.92596 |
| 3444.412 | 11.07589 |
| 3446.34 | 11.23173 |
| 3448.269 | 11.39879 |
| 3450.197 | 11.59439 |
| 3452.126 | 11.81155 |
| 3454.054 | 12.04557 |
| 3455.983 | 12.29661 |
| 3457.912 | 12.56261 |
| 3459.84 | 12.83886 |
| 3461.769 | 13.1195 |
| 3463.697 | 13.40667 |
| 3465.626 | 13.70017 |
| 3467.554 | 13.98963 |
| 3469.483 | 14.27344 |
| 3471.411 | 14.55865 |
| 3473.34 | 14.84477 |
| 3475.269 | 15.12496 |
| 3477.197 | 15.40631 |
| 3479.126 | 15.69263 |
| 3481.054 | 15.97943 |
| 3482.983 | 16.27831 |
| 3484.911 | 16.59849 |
| 3486.84 | 16.92629 |
| 3488.769 | 17.25117 |
| 3490.697 | 17.57552 |
| 3492.626 | 17.89548 |
| 3494.554 | 18.21286 |
| 3496.483 | 18.53364 |
| 3498.411 | 18.86713 |
| 3500.34 | 19.20869 |
| 3502.269 | 19.54433 |
| 3504.197 | 19.88919 |
| 3506.125 | 20.25914 |
| 3508.054 | 20.64041 |
| 3509.983 | 21.01807 |
| 3511.911 | 21.3942 |
| 3513.84 | 21.77328 |
| 3515.768 | 22.16492 |
| 3517.697 | 22.56166 |
| 3519.625 | 22.96035 |
| 3521.554 | 23.35177 |
| 3523.483 | 23.72332 |
| 3525.411 | 24.08898 |
| 3527.34 | 24.45275 |
| 3529.268 | 24.82648 |
| 3531.197 | 25.19859 |
| 3533.125 | 25.54478 |
| 3535.054 | 25.89584 |
| 3536.983 | 26.28051 |
| 3538.911 | 26.71745 |
| 3540.84 | 27.19337 |
| 3542.768 | 27.6794 |
| 3544.697 | 28.15825 |
| 3546.625 | 28.6404 |
| 3548.554 | 29.15404 |
| 3550.482 | 29.69071 |
| 3552.411 | 30.18718 |
| 3554.34 | 30.64791 |
| 3556.268 | 31.11444 |
| 3558.197 | 31.58795 |
| 3560.125 | 32.06546 |
| 3562.054 | 32.53891 |
| 3563.982 | 32.99591 |
| 3565.911 | 33.40451 |
| 3567.84 | 33.811 |
| 3569.768 | 34.33356 |
| 3571.697 | 34.95396 |
| 3573.625 | 35.59731 |
| 3575.554 | 36.24801 |
| 3577.482 | 36.90768 |
| 3579.411 | 37.56786 |
| 3581.34 | 38.25691 |
| 3583.268 | 38.94776 |
| 3585.197 | 39.56754 |
| 3587.125 | 40.1227 |
| 3589.054 | 40.71758 |
| 3590.982 | 41.41816 |
| 3592.911 | 42.13886 |
| 3594.839 | 42.771 |
| 3596.768 | 43.4021 |
| 3598.697 | 44.06381 |
| 3600.625 | 44.77022 |
| 3602.554 | 45.58425 |
| 3604.482 | 46.48385 |
| 3606.411 | 47.35727 |
| 3608.339 | 48.1558 |
| 3610.268 | 48.94578 |
| 3612.197 | 49.69089 |
| 3614.125 | 50.3194 |
| 3616.054 | 50.84153 |
| 3617.982 | 51.30058 |
| 3619.911 | 51.77686 |
| 3621.839 | 52.35757 |
| 3623.768 | 53.0761 |
| 3625.697 | 53.87608 |
| 3627.625 | 54.63947 |
| 3629.553 | 55.37683 |
| 3631.482 | 56.32174 |
| 3633.411 | 57.39392 |
| 3635.339 | 58.4802 |
| 3637.268 | 59.58502 |
| 3639.196 | 60.66265 |
| 3641.125 | 61.59344 |
| 3643.053 | 62.45128 |
| 3644.982 | 63.26551 |
| 3646.911 | 63.9574 |
| 3648.839 | 64.55071 |
| 3650.768 | 65.19115 |
| 3652.696 | 65.99512 |
| 3654.625 | 66.8771 |
| 3656.553 | 67.77585 |
| 3658.482 | 68.76324 |
| 3660.411 | 69.82838 |
| 3662.339 | 70.93143 |
| 3664.268 | 72.09812 |
| 3666.196 | 73.29129 |
| 3668.125 | 74.31926 |
| 3670.053 | 75.15168 |
| 3671.982 | 75.91224 |
| 3673.91 | 76.54337 |
| 3675.839 | 76.92709 |
| 3677.768 | 77.28275 |
| 3679.696 | 77.64539 |
| 3681.625 | 78.06478 |
| 3683.553 | 78.58689 |
| 3685.482 | 79.21068 |
| 3687.41 | 79.82514 |
| 3689.339 | 80.34511 |
| 3691.268 | 80.91787 |
| 3693.196 | 81.67858 |
| 3695.125 | 82.57439 |
| 3697.053 | 83.4567 |
| 3698.982 | 84.24408 |
| 3700.91 | 84.81423 |
| 3702.839 | 85.23469 |
| 3704.768 | 85.64213 |
| 3706.696 | 86.06806 |
| 3708.625 | 86.38369 |
| 3710.553 | 86.50104 |
| 3712.482 | 86.69049 |
| 3714.41 | 86.99818 |
| 3716.339 | 87.38412 |
| 3718.267 | 87.79476 |
| 3720.196 | 88.22907 |
| 3722.125 | 88.64718 |
| 3724.053 | 89.11469 |
| 3725.982 | 89.69164 |
| 3727.91 | 90.37834 |
| 3729.839 | 91.04395 |
| 3731.767 | 91.49741 |
| 3733.696 | 91.74812 |
| 3735.625 | 91.78419 |
| 3737.553 | 91.8014 |
| 3739.482 | 91.84219 |
| 3741.41 | 91.79435 |
| 3743.339 | 91.54309 |
| 3745.267 | 91.07974 |
| 3747.196 | 90.72537 |
| 3749.125 | 90.41183 |
| 3751.053 | 90.13434 |
| 3752.981 | 90.01668 |
| 3754.91 | 90.09643 |
| 3756.839 | 90.29071 |
| 3758.767 | 90.60197 |
| 3760.696 | 91.00114 |
| 3762.624 | 91.45683 |
| 3764.553 | 91.86491 |
| 3766.481 | 92.12293 |
| 3768.41 | 92.31136 |
| 3770.339 | 92.49754 |
| 3772.267 | 92.7435 |
| 3774.196 | 93.01923 |
| 3776.124 | 93.25996 |
| 3778.053 | 93.42468 |
| 3779.981 | 93.5386 |
| 3781.91 | 93.69418 |
| 3783.839 | 93.84129 |
| 3785.767 | 94.00446 |
| 3787.696 | 94.20884 |
| 3789.624 | 94.47969 |
| 3791.553 | 94.73857 |
| 3793.481 | 94.98772 |
| 3795.41 | 95.18844 |
| 3797.338 | 95.29296 |
| 3799.267 | 95.31113 |
| 3801.196 | 95.18208 |
| 3803.124 | 95.0239 |
| 3805.053 | 94.91023 |
| 3806.981 | 94.7913 |
| 3808.91 | 94.78001 |
| 3810.838 | 94.87775 |
| 3812.767 | 95.04224 |
| 3814.696 | 95.21196 |
| 3816.624 | 95.34485 |
| 3818.553 | 95.3916 |
| 3820.481 | 95.3005 |
| 3822.41 | 95.27895 |
| 3824.338 | 95.48013 |
| 3826.267 | 95.74533 |
| 3828.196 | 96.04591 |
| 3830.124 | 96.28896 |
| 3832.052 | 96.44682 |
| 3833.981 | 96.5586 |
| 3835.91 | 96.53128 |
| 3837.838 | 96.48082 |
| 3839.767 | 96.42785 |
| 3841.695 | 96.42376 |
| 3843.624 | 96.56976 |
| 3845.552 | 96.76189 |
| 3847.481 | 96.91684 |
| 3849.41 | 96.94767 |
| 3851.338 | 96.74427 |
| 3853.267 | 96.36242 |
| 3855.195 | 96.08017 |
| 3857.124 | 96.05801 |
| 3859.052 | 96.2174 |
| 3860.981 | 96.40124 |
| 3862.91 | 96.57073 |
| 3864.838 | 96.64523 |
| 3866.767 | 96.68163 |
| 3868.695 | 96.65549 |
| 3870.624 | 96.54562 |
| 3872.552 | 96.565 |
| 3874.481 | 96.69571 |
| 3876.409 | 96.99003 |
| 3878.338 | 97.35699 |
| 3880.267 | 97.64453 |
| 3882.195 | 97.84573 |
| 3884.124 | 97.93143 |
| 3886.052 | 97.87422 |
| 3887.981 | 97.8907 |
| 3889.909 | 98.00316 |
| 3891.838 | 98.09746 |
| 3893.767 | 98.18871 |
| 3895.695 | 98.22213 |
| 3897.624 | 98.1256 |
| 3899.552 | 97.9072 |
| 3901.481 | 97.65495 |
| 3903.409 | 97.38749 |
| 3905.338 | 97.22398 |
| 3907.267 | 97.26675 |
| 3909.195 | 97.39749 |
| 3911.124 | 97.60435 |
| 3913.052 | 97.79708 |
| 3914.981 | 97.93447 |
| 3916.909 | 98.05764 |
| 3918.838 | 98.17999 |
| 3920.766 | 98.30642 |
| 3922.695 | 98.47795 |
| 3924.624 | 98.586 |
| 3926.552 | 98.66929 |
| 3928.481 | 98.74706 |
| 3930.409 | 98.74139 |
| 3932.338 | 98.65879 |
| 3934.266 | 98.5723 |
| 3936.195 | 98.52602 |
| 3938.124 | 98.52025 |
| 3940.052 | 98.57084 |
| 3941.981 | 98.63583 |
| 3943.909 | 98.68243 |
| 3945.838 | 98.73053 |
| 3947.766 | 98.75368 |
| 3949.695 | 98.76503 |
| 3951.623 | 98.78985 |
| 3953.552 | 98.84378 |
| 3955.48 | 98.90839 |
| 3957.409 | 98.97164 |
| 3959.338 | 99.01896 |
| 3961.266 | 99.05823 |
| 3963.195 | 99.08711 |
| 3965.123 | 99.11901 |
| 3967.052 | 99.16698 |
| 3968.98 | 99.22758 |
| 3970.909 | 99.28046 |
| 3972.838 | 99.32516 |
| 3974.766 | 99.36632 |
| 3976.695 | 99.40621 |
| 3978.623 | 99.4603 |
| 3980.552 | 99.51637 |
| 3982.48 | 99.55061 |
| 3984.409 | 99.61195 |
| 3986.337 | 99.68191 |
| 3988.266 | 99.73466 |
| 3990.195 | 99.77032 |
| 3992.123 | 99.80387 |
| 3994.052 | 99.74229 |
| 3995.98 | 99.66424 |
| 3997.909 | 99.73782 |
| 3999.837 | 99.87508 |

Source for Table 2.

| Elt | Line | Int | Error | K | Kr | W% | A% | ZAF | Formula | Ox% | Pk/Bg | Class | LConf | HConf | Cat# |
| --- | --- | --- | --- | --- | --- | --- | --- | --- | --- | --- | --- | --- | --- | --- | --- |
| **C** | Ka | 124.6 | 14.5080 | 0.7124 | 0.3320 | 57.99 | 67.39 | 0.5724 |  | 0.00 | 454.49 | A | 56.10 | 59.89 | 0.00 |
| **O** | Ka | 49.4 | 14.5080 | 0.1407 | 0.0656 | 33.71 | 29.40 | 0.1945 |  | 0.00 | 187.19 | A | 31.96 | 35.45 | 0.00 |
| **Mg** | Ka | 7.8 | 5.0142 | 0.0080 | 0.0037 | 0.60 | 0.34 | 0.6217 |  | 0.00 | 9.30 | A | 0.52 | 0.67 | 0.00 |
| **Al** | Ka | 3.7 | 5.0142 | 0.0037 | 0.0017 | 0.24 | 0.13 | 0.7024 |  | 0.00 | 4.89 | A | 0.20 | 0.29 | 0.00 |
| **Si** | Ka | 7.5 | 5.0142 | 0.0079 | 0.0037 | 0.46 | 0.23 | 0.7943 |  | 0.00 | 6.09 | A | 0.40 | 0.52 | 0.00 |
| **Cl** | Ka | 12.4 | 2.1631 | 0.0184 | 0.0086 | 1.02 | 0.40 | 0.8392 |  | 0.00 | 8.51 | A | 0.92 | 1.13 | 0.00 |
| **K** | Ka | 33.8 | 0.9770 | 0.0610 | 0.0284 | 3.35 | 1.19 | 0.8493 |  | 0.00 | 18.45 | A | 3.14 | 3.55 | 0.00 |
| **Ca** | Ka | 23.9 | 0.9770 | 0.0481 | 0.0224 | 2.63 | 0.92 | 0.8520 |  | 0.00 | 14.50 | A | 2.43 | 2.83 | 0.00 |
|  |  |  |  | 1.0000 | 0.4661 | 100.00 | 100.00 |  |  | 0.00 |  |  |  |  | 0.00 |

| Elt | Line | Int | Error | K | Kr | W% | A% | ZAF | Formula | Ox% | Pk/Bg | Class | LConf | HConf | Cat# |
| --- | --- | --- | --- | --- | --- | --- | --- | --- | --- | --- | --- | --- | --- | --- | --- |
| **C** | Ka | 16.3 | 5.4797 | 0.3279 | 0.1252 | 34.50 | 44.10 | 0.3629 |  | 0.00 | 103.36 | A | 31.39 | 37.61 | 0.00 |
| **O** | Ka | 38.1 | 5.4797 | 0.3813 | 0.1456 | 51.19 | 49.13 | 0.2844 |  | 0.00 | 120.65 | A | 48.17 | 54.22 | 0.00 |
| **Mg** | Ka | 6.3 | 0.9372 | 0.0224 | 0.0086 | 1.45 | 0.92 | 0.5892 |  | 0.00 | 6.81 | A | 1.24 | 1.66 | 0.00 |
| **Al** | Ka | 6.5 | 0.9372 | 0.0226 | 0.0086 | 1.28 | 0.73 | 0.6735 |  | 0.00 | 6.16 | A | 1.10 | 1.47 | 0.00 |
| **Si** | Ka | 25.4 | 0.9372 | 0.0932 | 0.0356 | 4.64 | 2.54 | 0.7670 |  | 0.00 | 17.13 | A | 4.30 | 4.98 | 0.00 |
| **S** | Ka | 3.8 | 0.6289 | 0.0179 | 0.0069 | 0.82 | 0.39 | 0.8315 |  | 0.00 | 4.48 | A | 0.67 | 0.98 | 0.00 |
| **K** | Ka | 3.1 | 0.7889 | 0.0198 | 0.0076 | 0.89 | 0.35 | 0.8526 |  | 0.00 | 4.10 | A | 0.70 | 1.07 | 0.00 |
| **Ca** | Ka | 12.1 | 0.7889 | 0.0855 | 0.0327 | 3.77 | 1.45 | 0.8650 |  | 0.00 | 8.67 | A | 3.38 | 4.17 | 0.00 |
| **Fe** | Ka | 1.7 | 0.5406 | 0.0292 | 0.0112 | 1.45 | 0.40 | 0.7697 |  | 0.00 | 2.94 | B | 1.04 | 1.86 | 0.00 |
|  |  |  |  | 1.0000 | 0.3818 | 100.00 | 100.00 |  |  | 0.00 |  |  |  |  | 0.00 |

Source for Fig. 2.

| 10.1 | 299 |
| --- | --- |
| 10.15 | 352 |
| 10.2 | 363 |
| 10.25 | 399 |
| 10.3 | 399 |
| 10.35 | 352 |
| 10.4 | 360 |
| 10.45 | 371 |
| 10.5 | 368 |
| 10.55 | 380 |
| 10.6 | 330 |
| 10.65 | 378 |
| 10.7 | 380 |
| 10.75 | 401 |
| 10.8 | 371 |
| 10.85 | 391 |
| 10.9 | 390 |
| 10.95 | 368 |
| 11 | 393 |
| 11.05 | 381 |
| 11.1 | 381 |
| 11.15 | 389 |
| 11.2 | 431 |
| 11.25 | 360 |
| 11.3 | 359 |
| 11.35 | 400 |
| 11.4 | 372 |
| 11.45 | 352 |
| 11.5 | 349 |
| 11.55 | 382 |
| 11.6 | 420 |
| 11.65 | 371 |
| 11.7 | 369 |
| 11.75 | 410 |
| 11.8 | 372 |
| 11.85 | 392 |
| 11.9 | 391 |
| 11.95 | 380 |
| 12 | 431 |
| 12.05 | 403 |
| 12.1 | 402 |
| 12.15 | 442 |
| 12.2 | 410 |
| 12.25 | 420 |
| 12.3 | 420 |
| 12.35 | 430 |
| 12.4 | 421 |
| 12.45 | 433 |
| 12.5 | 433 |
| 12.55 | 379 |
| 12.6 | 410 |
| 12.65 | 382 |
| 12.7 | 382 |
| 12.75 | 422 |
| 12.8 | 392 |
| 12.85 | 412 |
| 12.9 | 412 |
| 12.95 | 453 |
| 13 | 458 |
| 13.05 | 429 |
| 13.1 | 430 |
| 13.15 | 428 |
| 13.2 | 448 |
| 13.25 | 443 |
| 13.3 | 439 |
| 13.35 | 383 |
| 13.4 | 379 |
| 13.45 | 409 |
| 13.5 | 409 |
| 13.55 | 401 |
| 13.6 | 450 |
| 13.65 | 430 |
| 13.7 | 432 |
| 13.75 | 452 |
| 13.8 | 513 |
| 13.85 | 391 |
| 13.9 | 390 |
| 13.95 | 438 |
| 14 | 462 |
| 14.05 | 481 |
| 14.1 | 482 |
| 14.15 | 511 |
| 14.2 | 491 |
| 14.25 | 490 |
| 14.3 | 492 |
| 14.35 | 573 |
| 14.4 | 542 |
| 14.45 | 493 |
| 14.5 | 489 |
| 14.55 | 568 |
| 14.6 | 599 |
| 14.65 | 681 |
| 14.7 | 680 |
| 14.75 | 711 |
| 14.8 | 888 |
| 14.85 | 883 |
| 14.9 | 880 |
| 14.95 | 860 |
| 15 | 670 |
| 15.05 | 660 |
| 15.1 | 659 |
| 15.15 | 591 |
| 15.2 | 651 |
| 15.25 | 670 |
| 15.3 | 669 |
| 15.35 | 619 |
| 15.4 | 570 |
| 15.45 | 469 |
| 15.5 | 469 |
| 15.55 | 499 |
| 15.6 | 472 |
| 15.65 | 490 |
| 15.7 | 490 |
| 15.75 | 533 |
| 15.8 | 521 |
| 15.85 | 528 |
| 15.9 | 529 |
| 15.95 | 529 |
| 16 | 489 |
| 16.05 | 522 |
| 16.1 | 522 |
| 16.15 | 532 |
| 16.2 | 529 |
| 16.25 | 531 |
| 16.3 | 531 |
| 16.35 | 522 |
| 16.4 | 531 |
| 16.45 | 531 |
| 16.5 | 528 |
| 16.55 | 582 |
| 16.6 | 510 |
| 16.65 | 491 |
| 16.7 | 492 |
| 16.75 | 530 |
| 16.8 | 560 |
| 16.85 | 511 |
| 16.9 | 512 |
| 16.95 | 509 |
| 17 | 511 |
| 17.05 | 540 |
| 17.1 | 542 |
| 17.15 | 538 |
| 17.2 | 573 |
| 17.25 | 502 |
| 17.3 | 501 |
| 17.35 | 529 |
| 17.4 | 551 |
| 17.45 | 539 |
| 17.5 | 541 |
| 17.55 | 499 |
| 17.6 | 512 |
| 17.65 | 512 |
| 17.7 | 539 |
| 17.75 | 512 |
| 17.8 | 593 |
| 17.85 | 591 |
| 17.9 | 551 |
| 17.95 | 511 |
| 18 | 573 |
| 18.05 | 571 |
| 18.1 | 601 |
| 18.15 | 561 |
| 18.2 | 632 |
| 18.25 | 633 |
| 18.3 | 600 |
| 18.35 | 519 |
| 18.4 | 621 |
| 18.45 | 619 |
| 18.5 | 583 |
| 18.55 | 581 |
| 18.6 | 522 |
| 18.65 | 520 |
| 18.7 | 602 |
| 18.75 | 582 |
| 18.8 | 523 |
| 18.85 | 518 |
| 18.9 | 630 |
| 18.95 | 572 |
| 19 | 622 |
| 19.05 | 623 |
| 19.1 | 560 |
| 19.15 | 640 |
| 19.2 | 629 |
| 19.25 | 631 |
| 19.3 | 632 |
| 19.35 | 590 |
| 19.4 | 619 |
| 19.45 | 623 |
| 19.5 | 590 |
| 19.55 | 549 |
| 19.6 | 631 |
| 19.65 | 629 |
| 19.7 | 631 |
| 19.75 | 593 |
| 19.8 | 641 |
| 19.85 | 639 |
| 19.9 | 670 |
| 19.95 | 590 |
| 20 | 629 |
| 20.05 | 630 |
| 20.1 | 681 |
| 20.15 | 629 |
| 20.2 | 632 |
| 20.25 | 631 |
| 20.3 | 620 |
| 20.35 | 549 |
| 20.4 | 561 |
| 20.45 | 559 |
| 20.5 | 628 |
| 20.55 | 610 |
| 20.6 | 651 |
| 20.65 | 649 |
| 20.7 | 629 |
| 20.75 | 622 |
| 20.8 | 611 |
| 20.85 | 612 |
| 20.9 | 619 |
| 20.95 | 580 |
| 21 | 631 |
| 21.05 | 632 |
| 21.1 | 599 |
| 21.15 | 639 |
| 21.2 | 641 |
| 21.25 | 639 |
| 21.3 | 641 |
| 21.35 | 590 |
| 21.4 | 669 |
| 21.45 | 671 |
| 21.5 | 591 |
| 21.55 | 680 |
| 21.6 | 682 |
| 21.65 | 681 |
| 21.7 | 579 |
| 21.75 | 559 |
| 21.8 | 650 |
| 21.85 | 653 |
| 21.9 | 621 |
| 21.95 | 650 |
| 22 | 633 |
| 22.05 | 632 |
| 22.1 | 582 |
| 22.15 | 609 |
| 22.2 | 609 |
| 22.25 | 611 |
| 22.3 | 673 |
| 22.35 | 548 |
| 22.4 | 590 |
| 22.45 | 591 |
| 22.5 | 579 |
| 22.55 | 620 |
| 22.6 | 562 |
| 22.65 | 562 |
| 22.7 | 581 |
| 22.75 | 591 |
| 22.8 | 592 |
| 22.85 | 590 |
| 22.9 | 581 |
| 22.95 | 610 |
| 23 | 619 |
| 23.05 | 620 |
| 23.1 | 568 |
| 23.15 | 593 |
| 23.2 | 631 |
| 23.25 | 629 |
| 23.3 | 541 |
| 23.35 | 588 |
| 23.4 | 631 |
| 23.45 | 632 |
| 23.5 | 633 |
| 23.55 | 610 |
| 23.6 | 599 |
| 23.65 | 598 |
| 23.7 | 542 |
| 23.75 | 519 |
| 23.8 | 541 |
| 23.85 | 539 |
| 23.9 | 630 |
| 23.95 | 610 |
| 24 | 603 |
| 24.05 | 602 |
| 24.1 | 682 |
| 24.15 | 751 |
| 24.2 | 849 |
| 24.25 | 851 |
| 24.3 | 961 |
| 24.35 | 1069 |
| 24.4 | 929 |
| 24.45 | 932 |
| 24.5 | 818 |
| 24.55 | 668 |
| 24.6 | 559 |
| 24.65 | 562 |
| 24.7 | 502 |
| 24.75 | 502 |
| 24.8 | 501 |
| 24.85 | 503 |
| 24.9 | 508 |
| 24.95 | 501 |
| 25 | 580 |
| 25.05 | 580 |
| 25.1 | 531 |
| 25.15 | 538 |
| 25.2 | 502 |
| 25.25 | 501 |
| 25.3 | 518 |
| 25.35 | 490 |
| 25.4 | 520 |
| 25.45 | 520 |
| 25.5 | 540 |
| 25.55 | 563 |
| 25.6 | 480 |
| 25.65 | 481 |
| 25.7 | 502 |
| 25.75 | 442 |
| 25.8 | 509 |
| 25.85 | 513 |
| 25.9 | 470 |
| 25.95 | 481 |
| 26 | 479 |
| 26.05 | 478 |
| 26.1 | 509 |
| 26.15 | 473 |
| 26.2 | 470 |
| 26.25 | 491 |
| 26.3 | 451 |
| 26.35 | 489 |
| 26.4 | 490 |
| 26.45 | 479 |
| 26.5 | 538 |
| 26.55 | 478 |
| 26.6 | 481 |
| 26.65 | 531 |
| 26.7 | 469 |
| 26.75 | 519 |
| 26.8 | 518 |
| 26.85 | 460 |
| 26.9 | 479 |
| 26.95 | 468 |
| 27 | 469 |
| 27.05 | 472 |
| 27.1 | 482 |
| 27.15 | 443 |
| 27.2 | 439 |
| 27.25 | 448 |
| 27.3 | 418 |
| 27.35 | 439 |
| 27.4 | 440 |
| 27.45 | 471 |
| 27.5 | 408 |
| 27.55 | 422 |
| 27.6 | 419 |
| 27.65 | 428 |
| 27.7 | 423 |
| 27.75 | 442 |
| 27.8 | 439 |
| 27.85 | 462 |
| 27.9 | 443 |
| 27.95 | 479 |
| 28 | 480 |
| 28.05 | 430 |
| 28.1 | 479 |
| 28.15 | 421 |
| 28.2 | 420 |
| 28.25 | 433 |
| 28.3 | 462 |
| 28.35 | 412 |
| 28.4 | 408 |
| 28.45 | 480 |
| 28.5 | 371 |
| 28.55 | 452 |
| 28.6 | 451 |
| 28.65 | 429 |
| 28.7 | 413 |
| 28.75 | 438 |
| 28.8 | 441 |
| 28.85 | 391 |
| 28.9 | 419 |
| 28.95 | 429 |
| 29 | 429 |
| 29.05 | 430 |
| 29.1 | 430 |
| 29.15 | 489 |
| 29.2 | 490 |
| 29.25 | 461 |
| 29.3 | 470 |
| 29.35 | 480 |
| 29.4 | 481 |
| 29.45 | 532 |
| 29.5 | 458 |
| 29.55 | 458 |
| 29.6 | 462 |
| 29.65 | 500 |
| 29.7 | 478 |
| 29.75 | 489 |
| 29.8 | 490 |
| 29.85 | 479 |
| 29.9 | 519 |
| 29.95 | 532 |
| 30 | 531 |
| 30.05 | 579 |
| 30.1 | 602 |
| 30.15 | 613 |
| 30.2 | 611 |
| 30.25 | 529 |
| 30.3 | 432 |
| 30.35 | 453 |
| 30.4 | 449 |
| 30.45 | 430 |
| 30.5 | 429 |
| 30.55 | 461 |
| 30.6 | 459 |
| 30.65 | 449 |
| 30.7 | 453 |
| 30.75 | 493 |
| 30.8 | 491 |
| 30.85 | 479 |
| 30.9 | 459 |
| 30.95 | 420 |
| 31 | 418 |
| 31.05 | 371 |
| 31.1 | 411 |
| 31.15 | 379 |
| 31.2 | 380 |
| 31.25 | 381 |
| 31.3 | 419 |
| 31.35 | 451 |
| 31.4 | 452 |
| 31.45 | 451 |
| 31.5 | 390 |
| 31.55 | 371 |
| 31.6 | 371 |
| 31.65 | 363 |
| 31.7 | 398 |
| 31.75 | 391 |
| 31.8 | 389 |
| 31.85 | 371 |
| 31.9 | 390 |
| 31.95 | 399 |
| 32 | 399 |
| 32.05 | 399 |
| 32.1 | 412 |
| 32.15 | 379 |
| 32.2 | 380 |
| 32.25 | 381 |
| 32.3 | 371 |
| 32.35 | 371 |
| 32.4 | 370 |
| 32.45 | 382 |
| 32.5 | 350 |
| 32.55 | 343 |
| 32.6 | 343 |
| 32.65 | 340 |
| 32.7 | 383 |
| 32.75 | 350 |
| 32.8 | 353 |
| 32.85 | 358 |
| 32.9 | 390 |
| 32.95 | 339 |
| 33 | 338 |
| 33.05 | 370 |
| 33.1 | 320 |
| 33.15 | 330 |
| 33.2 | 328 |
| 33.25 | 331 |
| 33.3 | 310 |
| 33.35 | 368 |
| 33.4 | 368 |
| 33.45 | 349 |
| 33.5 | 311 |
| 33.55 | 312 |
| 33.6 | 309 |
| 33.65 | 412 |
| 33.7 | 318 |
| 33.75 | 352 |
| 33.8 | 353 |
| 33.85 | 339 |
| 33.9 | 353 |
| 33.95 | 378 |
| 34 | 380 |
| 34.05 | 310 |
| 34.1 | 341 |
| 34.15 | 350 |
| 34.2 | 350 |
| 34.25 | 369 |
| 34.3 | 360 |
| 34.35 | 313 |
| 34.4 | 309 |
| 34.45 | 403 |
| 34.5 | 309 |
| 34.55 | 350 |
| 34.6 | 348 |
| 34.65 | 402 |
| 34.7 | 340 |
| 34.75 | 341 |
| 34.8 | 329 |
| 34.85 | 330 |
| 34.9 | 360 |
| 34.95 | 359 |
| 35 | 359 |
| 35.05 | 339 |
| 35.1 | 371 |
| 35.15 | 372 |
| 35.2 | 329 |
| 35.25 | 369 |
| 35.3 | 390 |
| 35.35 | 388 |
| 35.4 | 328 |
| 35.45 | 381 |
| 35.5 | 372 |
| 35.55 | 370 |
| 35.6 | 393 |
| 35.65 | 351 |
| 35.7 | 398 |
| 35.75 | 399 |
| 35.8 | 438 |
| 35.85 | 460 |
| 35.9 | 458 |
| 35.95 | 460 |
| 36 | 513 |
| 36.05 | 488 |
| 36.1 | 388 |
| 36.15 | 391 |
| 36.2 | 442 |
| 36.25 | 392 |
| 36.3 | 372 |
| 36.35 | 368 |
| 36.4 | 373 |
| 36.45 | 393 |
| 36.5 | 371 |
| 36.55 | 370 |
| 36.6 | 360 |
| 36.65 | 341 |
| 36.7 | 301 |
| 36.75 | 301 |
| 36.8 | 331 |
| 36.85 | 373 |
| 36.9 | 372 |
| 36.95 | 369 |
| 37 | 381 |
| 37.05 | 332 |
| 37.1 | 359 |
| 37.15 | 360 |
| 37.2 | 362 |
| 37.25 | 381 |
| 37.3 | 389 |
| 37.35 | 392 |
| 37.4 | 371 |
| 37.45 | 401 |
| 37.5 | 361 |
| 37.55 | 359 |
| 37.6 | 400 |
| 37.65 | 362 |
| 37.7 | 390 |
| 37.75 | 392 |
| 37.8 | 390 |
| 37.85 | 351 |
| 37.9 | 441 |
| 37.95 | 442 |
| 38 | 460 |
| 38.05 | 441 |
| 38.1 | 589 |
| 38.15 | 589 |
| 38.2 | 562 |
| 38.25 | 561 |
| 38.3 | 561 |
| 38.35 | 559 |
| 38.4 | 421 |
| 38.45 | 448 |
| 38.5 | 380 |
| 38.55 | 383 |
| 38.6 | 350 |
| 38.65 | 389 |
| 38.7 | 331 |
| 38.75 | 333 |
| 38.8 | 349 |
| 38.85 | 329 |
| 38.9 | 341 |
| 38.95 | 343 |
| 39 | 350 |
| 39.05 | 382 |
| 39.1 | 361 |
| 39.15 | 358 |
| 39.2 | 329 |
| 39.25 | 342 |
| 39.3 | 318 |
| 39.35 | 319 |
| 39.4 | 351 |
| 39.45 | 420 |
| 39.5 | 360 |
| 39.55 | 363 |
| 39.6 | 402 |
| 39.65 | 369 |
| 39.7 | 422 |
| 39.75 | 418 |
| 39.8 | 389 |
| 39.85 | 383 |
| 39.9 | 372 |
| 39.95 | 372 |
| 40 | 382 |
| 40.05 | 401 |
| 40.1 | 321 |
| 40.15 | 320 |
| 40.2 | 302 |
| 40.25 | 361 |
| 40.3 | 351 |
| 40.35 | 348 |
| 40.4 | 343 |
| 40.45 | 340 |
| 40.5 | 339 |
| 40.55 | 342 |
| 40.6 | 358 |
| 40.65 | 320 |
| 40.7 | 330 |
| 40.75 | 331 |
| 40.8 | 352 |
| 40.85 | 358 |
| 40.9 | 319 |
| 40.95 | 320 |
| 41 | 300 |
| 41.05 | 310 |
| 41.1 | 311 |
| 41.15 | 311 |
| 41.2 | 351 |
| 41.25 | 311 |
| 41.3 | 379 |
| 41.35 | 382 |
| 41.4 | 343 |
| 41.45 | 332 |
| 41.5 | 291 |
| 41.55 | 291 |
| 41.6 | 360 |
| 41.65 | 290 |
| 41.7 | 292 |
| 41.75 | 289 |
| 41.8 | 340 |
| 41.85 | 309 |
| 41.9 | 343 |
| 41.95 | 342 |
| 42 | 340 |
| 42.05 | 401 |
| 42.1 | 343 |
| 42.15 | 342 |
| 42.2 | 289 |
| 42.25 | 311 |
| 42.3 | 308 |
| 42.35 | 311 |
| 42.4 | 322 |
| 42.45 | 319 |
| 42.5 | 302 |
| 42.55 | 299 |
| 42.6 | 360 |
| 42.65 | 320 |
| 42.7 | 372 |
| 42.75 | 370 |
| 42.8 | 319 |
| 42.85 | 312 |
| 42.9 | 329 |
| 42.95 | 328 |
| 43 | 310 |
| 43.05 | 331 |
| 43.1 | 330 |
| 43.15 | 332 |
| 43.2 | 379 |
| 43.25 | 342 |
| 43.3 | 349 |
| 43.35 | 349 |
| 43.4 | 361 |
| 43.45 | 322 |
| 43.5 | 360 |
| 43.55 | 360 |
| 43.6 | 398 |
| 43.65 | 343 |
| 43.7 | 341 |
| 43.75 | 372 |
| 43.8 | 340 |
| 43.85 | 312 |
| 43.9 | 310 |
| 43.95 | 319 |
| 44 | 282 |
| 44.05 | 329 |
| 44.1 | 332 |
| 44.15 | 269 |
| 44.2 | 271 |
| 44.25 | 270 |
| 44.3 | 270 |
| 44.35 | 311 |
| 44.4 | 323 |
| 44.45 | 303 |
| 44.5 | 302 |
| 44.55 | 280 |
| 44.6 | 299 |
| 44.65 | 301 |
| 44.7 | 299 |
| 44.75 | 302 |
| 44.8 | 311 |
| 44.85 | 359 |
| 44.9 | 362 |
| 44.95 | 280 |
| 45 | 288 |
| 45.05 | 272 |
| 45.1 | 271 |
| 45.15 | 288 |
| 45.2 | 311 |
| 45.25 | 279 |
| 45.3 | 281 |
| 45.35 | 329 |
| 45.4 | 323 |
| 45.45 | 322 |
| 45.5 | 320 |
| 45.55 | 343 |
| 45.6 | 303 |
| 45.65 | 292 |
| 45.7 | 290 |
| 45.75 | 393 |
| 45.8 | 363 |
| 45.85 | 349 |
| 45.9 | 350 |
| 45.95 | 323 |
| 46 | 342 |
| 46.05 | 362 |
| 46.1 | 361 |
| 46.15 | 292 |
| 46.2 | 349 |
| 46.25 | 332 |
| 46.3 | 333 |
| 46.35 | 309 |
| 46.4 | 290 |
| 46.45 | 362 |
| 46.5 | 358 |
| 46.55 | 333 |
| 46.6 | 329 |
| 46.65 | 369 |
| 46.7 | 372 |
| 46.75 | 309 |
| 46.8 | 360 |
| 46.85 | 319 |
| 46.9 | 322 |
| 46.95 | 340 |
| 47 | 340 |
| 47.05 | 332 |
| 47.1 | 330 |
| 47.15 | 331 |
| 47.2 | 339 |
| 47.25 | 322 |
| 47.3 | 320 |
| 47.35 | 323 |
| 47.4 | 269 |
| 47.45 | 273 |
| 47.5 | 273 |
| 47.55 | 323 |
| 47.6 | 302 |
| 47.65 | 339 |
| 47.7 | 340 |
| 47.75 | 320 |
| 47.8 | 291 |
| 47.85 | 291 |
| 47.9 | 288 |
| 47.95 | 323 |
| 48 | 272 |
| 48.05 | 300 |
| 48.1 | 300 |
| 48.15 | 319 |
| 48.2 | 281 |
| 48.25 | 290 |
| 48.3 | 291 |
| 48.35 | 320 |
| 48.4 | 319 |
| 48.45 | 272 |
| 48.5 | 269 |
| 48.55 | 291 |
| 48.6 | 301 |
| 48.65 | 239 |
| 48.7 | 240 |
| 48.75 | 280 |
| 48.8 | 289 |
| 48.85 | 270 |
| 48.9 | 270 |
| 48.95 | 322 |
| 49 | 303 |
| 49.05 | 270 |
| 49.1 | 272 |
| 49.15 | 262 |
| 49.2 | 292 |
| 49.25 | 328 |
| 49.3 | 332 |
| 49.35 | 259 |
| 49.4 | 272 |
| 49.45 | 263 |
| 49.5 | 262 |
| 49.55 | 270 |
| 49.6 | 261 |
| 49.65 | 258 |
| 49.7 | 261 |
| 49.75 | 291 |
| 49.8 | 251 |
| 49.85 | 273 |
| 49.9 | 272 |
| 49.95 | 293 |
| 50 | 340 |
| 50.05 | 279 |
| 50.1 | 278 |
| 50.15 | 272 |
| 50.2 | 278 |
| 50.25 | 301 |
| 50.3 | 302 |
| 50.35 | 270 |
| 50.4 | 301 |
| 50.45 | 292 |
| 50.5 | 290 |
| 50.55 | 292 |
| 50.6 | 282 |
| 50.65 | 290 |
| 50.7 | 290 |
| 50.75 | 272 |
| 50.8 | 281 |
| 50.85 | 279 |
| 50.9 | 281 |
| 50.95 | 310 |
| 51 | 281 |
| 51.05 | 263 |
| 51.1 | 263 |
| 51.15 | 250 |
| 51.2 | 251 |
| 51.25 | 280 |
| 51.3 | 279 |
| 51.35 | 232 |
| 51.4 | 230 |
| 51.45 | 223 |
| 51.5 | 221 |
| 51.55 | 222 |
| 51.6 | 201 |
| 51.65 | 190 |
| 51.7 | 191 |
| 51.75 | 209 |
| 51.8 | 221 |
| 51.85 | 200 |
| 51.9 | 202 |
| 51.95 | 202 |
| 52 | 228 |
| 52.05 | 259 |
| 52.1 | 258 |
| 52.15 | 230 |
| 52.2 | 240 |
| 52.25 | 240 |
| 52.3 | 230 |
| 52.35 | 241 |
| 52.4 | 249 |
| 52.45 | 248 |
| 52.5 | 222 |
| 52.55 | 253 |
| 52.6 | 232 |
| 52.65 | 232 |
| 52.7 | 262 |
| 52.75 | 210 |
| 52.8 | 229 |
| 52.85 | 229 |
| 52.9 | 208 |
| 52.95 | 241 |
| 53 | 238 |
| 53.05 | 239 |
| 53.1 | 232 |
| 53.15 | 219 |
| 53.2 | 212 |
| 53.25 | 209 |
| 53.3 | 192 |
| 53.35 | 220 |
| 53.4 | 230 |
| 53.45 | 232 |
| 53.5 | 222 |
| 53.55 | 210 |
| 53.6 | 222 |
| 53.65 | 220 |
| 53.7 | 220 |
| 53.75 | 189 |
| 53.8 | 240 |
| 53.85 | 242 |
| 53.9 | 270 |
| 53.95 | 242 |
| 54 | 213 |
| 54.05 | 209 |
| 54.1 | 229 |
| 54.15 | 221 |
| 54.2 | 170 |
| 54.25 | 170 |
| 54.3 | 239 |
| 54.35 | 219 |
| 54.4 | 219 |
| 54.45 | 222 |
| 54.5 | 161 |
| 54.55 | 222 |
| 54.6 | 203 |
| 54.65 | 200 |
| 54.7 | 212 |
| 54.75 | 229 |
| 54.8 | 219 |
| 54.85 | 219 |
| 54.9 | 222 |
| 54.95 | 199 |
| 55 | 211 |
| 55.05 | 210 |
| 55.1 | 201 |
| 55.15 | 193 |
| 55.2 | 192 |
| 55.25 | 192 |
| 55.3 | 198 |
| 55.35 | 200 |
| 55.4 | 219 |
| 55.45 | 220 |
| 55.5 | 213 |
| 55.55 | 240 |
| 55.6 | 212 |
| 55.65 | 209 |
| 55.7 | 253 |
| 55.75 | 192 |
| 55.8 | 240 |
| 55.85 | 241 |
| 55.9 | 160 |
| 55.95 | 189 |
| 56 | 211 |
| 56.05 | 209 |
| 56.1 | 180 |
| 56.15 | 211 |
| 56.2 | 210 |
| 56.25 | 212 |
| 56.3 | 232 |
| 56.35 | 178 |
| 56.4 | 199 |
| 56.45 | 199 |
| 56.5 | 180 |
| 56.55 | 200 |
| 56.6 | 213 |
| 56.65 | 209 |
| 56.7 | 202 |
| 56.75 | 213 |
| 56.8 | 238 |
| 56.85 | 239 |
| 56.9 | 241 |
| 56.95 | 212 |
| 57 | 232 |
| 57.05 | 230 |
| 57.1 | 209 |
| 57.15 | 208 |
| 57.2 | 189 |
| 57.25 | 189 |
| 57.3 | 203 |
| 57.35 | 190 |
| 57.4 | 213 |
| 57.45 | 212 |
| 57.5 | 192 |
| 57.55 | 201 |
| 57.6 | 183 |
| 57.65 | 180 |
| 57.7 | 203 |
| 57.75 | 171 |
| 57.8 | 221 |
| 57.85 | 223 |
| 57.9 | 219 |
| 57.95 | 209 |
| 58 | 210 |
| 58.05 | 210 |
| 58.1 | 230 |
| 58.15 | 179 |
| 58.2 | 152 |
| 58.25 | 152 |
| 58.3 | 179 |
| 58.35 | 179 |
| 58.4 | 250 |
| 58.45 | 248 |
| 58.5 | 202 |
| 58.55 | 189 |
| 58.6 | 200 |
| 58.65 | 199 |
| 58.7 | 209 |
| 58.75 | 179 |
| 58.8 | 160 |
| 58.85 | 159 |
| 58.9 | 202 |
| 58.95 | 202 |
| 59 | 199 |
| 59.05 | 201 |
| 59.1 | 199 |
| 59.15 | 220 |
| 59.2 | 230 |
| 59.25 | 231 |
| 59.3 | 211 |
| 59.35 | 229 |
| 59.4 | 199 |
| 59.45 | 202 |
| 59.5 | 223 |
| 59.55 | 230 |
| 59.6 | 211 |
| 59.65 | 210 |
| 59.7 | 201 |
| 59.75 | 231 |
| 59.8 | 231 |
| 59.85 | 232 |
| 59.9 | 202 |
| 59.95 | 210 |
| 60 | 201 |
| 60.05 | 201 |
| 60.1 | 201 |
| 60.15 | 212 |
| 60.2 | 180 |
| 60.25 | 178 |
| 60.3 | 210 |
| 60.35 | 242 |
| 60.4 | 189 |
| 60.45 | 191 |
| 60.5 | 240 |
| 60.55 | 220 |
| 60.6 | 192 |
| 60.65 | 188 |
| 60.7 | 222 |
| 60.75 | 182 |
| 60.8 | 183 |
| 60.85 | 239 |
| 60.9 | 210 |
| 60.95 | 220 |
| 61 | 222 |
| 61.05 | 221 |
| 61.1 | 171 |
| 61.15 | 200 |
| 61.2 | 199 |
| 61.25 | 190 |
| 61.3 | 178 |
| 61.35 | 192 |
| 61.4 | 193 |
| 61.45 | 211 |
| 61.5 | 220 |
| 61.55 | 213 |
| 61.6 | 213 |
| 61.65 | 170 |
| 61.7 | 219 |
| 61.75 | 188 |
| 61.8 | 191 |
| 61.85 | 168 |
| 61.9 | 189 |
| 61.95 | 162 |
| 62 | 160 |
| 62.05 | 148 |
| 62.1 | 169 |
| 62.15 | 183 |
| 62.2 | 179 |
| 62.25 | 211 |
| 62.3 | 193 |
| 62.35 | 180 |
| 62.4 | 180 |
| 62.45 | 201 |
| 62.5 | 179 |
| 62.55 | 232 |
| 62.6 | 232 |
| 62.65 | 238 |
| 62.7 | 219 |
| 62.75 | 213 |
| 62.8 | 208 |
| 62.85 | 212 |
| 62.9 | 240 |
| 62.95 | 223 |
| 63 | 222 |
| 63.05 | 181 |
| 63.1 | 172 |
| 63.15 | 153 |
| 63.2 | 151 |
| 63.25 | 172 |
| 63.3 | 162 |
| 63.35 | 178 |
| 63.4 | 180 |
| 63.45 | 232 |
| 63.5 | 162 |
| 63.55 | 221 |
| 63.6 | 218 |
| 63.65 | 182 |
| 63.7 | 200 |
| 63.75 | 200 |
| 63.8 | 200 |
| 63.85 | 211 |
| 63.9 | 190 |
| 63.95 | 140 |
| 64 | 141 |
| 64.05 | 192 |
| 64.1 | 191 |
| 64.15 | 172 |
| 64.2 | 170 |
| 64.25 | 219 |
| 64.3 | 192 |
| 64.35 | 179 |
| 64.4 | 179 |
| 64.45 | 149 |
| 64.5 | 220 |
| 64.55 | 220 |
| 64.6 | 219 |
| 64.65 | 162 |
| 64.7 | 189 |
| 64.75 | 192 |
| 64.8 | 190 |
| 64.85 | 212 |
| 64.9 | 201 |
| 64.95 | 200 |
| 65 | 202 |
| 65.05 | 219 |
| 65.1 | 190 |
| 65.15 | 192 |
| 65.2 | 191 |
| 65.25 | 189 |
| 65.3 | 192 |
| 65.35 | 180 |
| 65.4 | 181 |
| 65.45 | 148 |
| 65.5 | 220 |
| 65.55 | 169 |
| 65.6 | 172 |
| 65.65 | 159 |
| 65.7 | 193 |
| 65.75 | 158 |
| 65.8 | 158 |
| 65.85 | 170 |
| 65.9 | 181 |
| 65.95 | 179 |
| 66 | 181 |
| 66.05 | 192 |
| 66.1 | 179 |
| 66.15 | 200 |
| 66.2 | 202 |
| 66.25 | 179 |
| 66.3 | 159 |
| 66.35 | 202 |
| 66.4 | 199 |
| 66.45 | 193 |
| 66.5 | 200 |
| 66.55 | 202 |
| 66.6 | 202 |
| 66.65 | 168 |
| 66.7 | 198 |
| 66.75 | 178 |
| 66.8 | 183 |
| 66.85 | 200 |
| 66.9 | 171 |
| 66.95 | 169 |
| 67 | 170 |
| 67.05 | 222 |
| 67.1 | 222 |
| 67.15 | 192 |
| 67.2 | 193 |
| 67.25 | 159 |
| 67.3 | 200 |
| 67.35 | 209 |
| 67.4 | 212 |
| 67.45 | 152 |
| 67.5 | 239 |
| 67.55 | 192 |
| 67.6 | 189 |
| 67.65 | 198 |
| 67.7 | 179 |
| 67.75 | 162 |
| 67.8 | 163 |
| 67.85 | 200 |
| 67.9 | 159 |
| 67.95 | 222 |
| 68 | 220 |
| 68.05 | 178 |
| 68.1 | 190 |
| 68.15 | 172 |
| 68.2 | 171 |
| 68.25 | 209 |
| 68.3 | 180 |
| 68.35 | 219 |
| 68.4 | 219 |
| 68.45 | 201 |
| 68.5 | 149 |
| 68.55 | 193 |
| 68.6 | 190 |
| 68.65 | 180 |
| 68.7 | 159 |
| 68.75 | 200 |
| 68.8 | 199 |
| 68.85 | 171 |
| 68.9 | 211 |
| 68.95 | 168 |
| 69 | 171 |
| 69.05 | 178 |
| 69.1 | 159 |
| 69.15 | 191 |
| 69.2 | 189 |
| 69.25 | 169 |
| 69.3 | 171 |
| 69.35 | 171 |
| 69.4 | 181 |
| 69.45 | 159 |
| 69.5 | 209 |
| 69.55 | 211 |
| 69.6 | 168 |
| 69.65 | 171 |
| 69.7 | 169 |
| 69.75 | 170 |
| 69.8 | 162 |
| 69.85 | 158 |
| 69.9 | 153 |
| 69.95 | 150 |
| 70 | 211 |
| 70.05 | 178 |
| 70.1 | 213 |
| 70.15 | 210 |
| 70.2 | 192 |
| 70.25 | 190 |
| 70.3 | 191 |
| 70.35 | 189 |
| 70.4 | 179 |
| 70.45 | 212 |
| 70.5 | 180 |
| 70.55 | 179 |
| 70.6 | 171 |
| 70.65 | 213 |
| 70.7 | 152 |
| 70.75 | 148 |
| 70.8 | 179 |
| 70.85 | 220 |
| 70.9 | 233 |
| 70.95 | 231 |
| 71 | 203 |
| 71.05 | 138 |
| 71.1 | 198 |
| 71.15 | 202 |
| 71.2 | 191 |
| 71.25 | 209 |
| 71.3 | 171 |
| 71.35 | 169 |
| 71.4 | 199 |
| 71.45 | 171 |
| 71.5 | 191 |
| 71.55 | 192 |
| 71.6 | 181 |
| 71.65 | 169 |
| 71.7 | 200 |
| 71.75 | 201 |
| 71.8 | 201 |
| 71.85 | 201 |
| 71.9 | 202 |
| 71.95 | 202 |
| 72 | 162 |
| 72.05 | 188 |
| 72.1 | 228 |
| 72.15 | 231 |
| 72.2 | 211 |
| 72.25 | 209 |
| 72.3 | 268 |
| 72.35 | 271 |
| 72.4 | 220 |
| 72.45 | 230 |
| 72.5 | 239 |
| 72.55 | 243 |
| 72.6 | 279 |
| 72.65 | 253 |
| 72.7 | 252 |
| 72.75 | 251 |
| 72.8 | 230 |
| 72.85 | 209 |
| 72.9 | 248 |
| 72.95 | 248 |
| 73 | 258 |
| 73.05 | 201 |
| 73.1 | 171 |
| 73.15 | 172 |
| 73.2 | 189 |
| 73.25 | 210 |
| 73.3 | 179 |
| 73.35 | 181 |
| 73.4 | 210 |
| 73.45 | 160 |
| 73.5 | 180 |
| 73.55 | 182 |
| 73.6 | 208 |
| 73.65 | 198 |
| 73.7 | 158 |
| 73.75 | 161 |
| 73.8 | 191 |
| 73.85 | 200 |
| 73.9 | 190 |
| 73.95 | 191 |
| 74 | 201 |
| 74.05 | 208 |
| 74.1 | 212 |
| 74.15 | 210 |
| 74.2 | 212 |
| 74.25 | 229 |
| 74.3 | 211 |
| 74.35 | 210 |
| 74.4 | 181 |
| 74.45 | 150 |
| 74.5 | 172 |
| 74.55 | 169 |
| 74.6 | 199 |
| 74.65 | 182 |
| 74.7 | 180 |
| 74.75 | 180 |
| 74.8 | 219 |
| 74.85 | 179 |
| 74.9 | 190 |
| 74.95 | 189 |
| 75 | 171 |
| 75.05 | 222 |
| 75.1 | 200 |
| 75.15 | 199 |
| 75.2 | 212 |
| 75.25 | 179 |
| 75.3 | 179 |
| 75.35 | 181 |
| 75.4 | 171 |
| 75.45 | 190 |
| 75.5 | 172 |
| 75.55 | 170 |
| 75.6 | 229 |
| 75.65 | 189 |
| 75.7 | 180 |
| 75.75 | 181 |
| 75.8 | 168 |
| 75.85 | 230 |
| 75.9 | 191 |
| 75.95 | 192 |
| 76 | 192 |
| 76.05 | 199 |
| 76.1 | 179 |
| 76.15 | 178 |
| 76.2 | 189 |
| 76.25 | 180 |
| 76.3 | 202 |
| 76.35 | 201 |
| 76.4 | 180 |
| 76.45 | 201 |
| 76.5 | 190 |
| 76.55 | 188 |
| 76.6 | 200 |
| 76.65 | 172 |
| 76.7 | 220 |
| 76.75 | 220 |
| 76.8 | 219 |
| 76.85 | 210 |
| 76.9 | 178 |
| 76.95 | 180 |
| 77 | 190 |
| 77.05 | 181 |
| 77.1 | 223 |
| 77.15 | 219 |
| 77.2 | 208 |
| 77.25 | 180 |
| 77.3 | 161 |
| 77.35 | 160 |
| 77.4 | 182 |
| 77.45 | 202 |
| 77.5 | 221 |
| 77.55 | 222 |
| 77.6 | 200 |
| 77.65 | 179 |
| 77.7 | 282 |
| 77.75 | 211 |
| 77.8 | 203 |
| 77.85 | 171 |
| 77.9 | 171 |
| 77.95 | 228 |
| 78 | 203 |
| 78.05 | 209 |
| 78.1 | 213 |
| 78.15 | 221 |
| 78.2 | 211 |
| 78.25 | 213 |
| 78.3 | 210 |
| 78.35 | 200 |
| 78.4 | 192 |
| 78.45 | 218 |
| 78.5 | 222 |
| 78.55 | 201 |
| 78.6 | 190 |
| 78.65 | 181 |
| 78.7 | 178 |
| 78.75 | 182 |
| 78.8 | 213 |
| 78.85 | 221 |
| 78.9 | 222 |
| 78.95 | 202 |
| 79 | 192 |
| 79.05 | 190 |
| 79.1 | 191 |
| 79.15 | 220 |
| 79.2 | 221 |
| 79.25 | 239 |
| 79.3 | 242 |
| 79.35 | 213 |
| 79.4 | 212 |
| 79.45 | 212 |
| 79.5 | 210 |
| 79.55 | 198 |
| 79.6 | 240 |
| 79.65 | 189 |
| 79.7 | 188 |
| 79.75 | 162 |
| 79.8 | 228 |
| 79.85 | 201 |
| 79.9 | 198 |
| 79.95 | 202 |
| 80 | 191 |

| 10.1 | 252 |
| --- | --- |
| 10.15 | 292 |
| 10.2 | 330 |
| 10.25 | 328 |
| 10.3 | 330 |
| 10.35 | 300 |
| 10.4 | 320 |
| 10.45 | 350 |
| 10.5 | 350 |
| 10.55 | 349 |
| 10.6 | 283 |
| 10.65 | 299 |
| 10.7 | 299 |
| 10.75 | 383 |
| 10.8 | 318 |
| 10.85 | 353 |
| 10.9 | 349 |
| 10.95 | 319 |
| 11 | 358 |
| 11.05 | 322 |
| 11.1 | 322 |
| 11.15 | 320 |
| 11.2 | 400 |
| 11.25 | 328 |
| 11.3 | 332 |
| 11.35 | 361 |
| 11.4 | 350 |
| 11.45 | 340 |
| 11.5 | 342 |
| 11.55 | 350 |
| 11.6 | 349 |
| 11.65 | 341 |
| 11.7 | 338 |
| 11.75 | 330 |
| 11.8 | 330 |
| 11.85 | 371 |
| 11.9 | 371 |
| 11.95 | 331 |
| 12 | 390 |
| 12.05 | 349 |
| 12.1 | 348 |
| 12.15 | 353 |
| 12.2 | 391 |
| 12.25 | 369 |
| 12.3 | 370 |
| 12.35 | 368 |
| 12.4 | 313 |
| 12.45 | 359 |
| 12.5 | 358 |
| 12.55 | 399 |
| 12.6 | 349 |
| 12.65 | 369 |
| 12.7 | 370 |
| 12.75 | 382 |
| 12.8 | 432 |
| 12.85 | 381 |
| 12.9 | 381 |
| 12.95 | 361 |
| 13 | 352 |
| 13.05 | 390 |
| 13.1 | 391 |
| 13.15 | 353 |
| 13.2 | 390 |
| 13.25 | 382 |
| 13.3 | 383 |
| 13.35 | 412 |
| 13.4 | 382 |
| 13.45 | 361 |
| 13.5 | 362 |
| 13.55 | 389 |
| 13.6 | 449 |
| 13.65 | 410 |
| 13.7 | 408 |
| 13.75 | 359 |
| 13.8 | 410 |
| 13.85 | 421 |
| 13.9 | 422 |
| 13.95 | 413 |
| 14 | 422 |
| 14.05 | 422 |
| 14.1 | 419 |
| 14.15 | 409 |
| 14.2 | 493 |
| 14.25 | 489 |
| 14.3 | 353 |
| 14.35 | 440 |
| 14.4 | 442 |
| 14.45 | 441 |
| 14.5 | 443 |
| 14.55 | 442 |
| 14.6 | 461 |
| 14.65 | 463 |
| 14.7 | 408 |
| 14.75 | 438 |
| 14.8 | 432 |
| 14.85 | 433 |
| 14.9 | 458 |
| 14.95 | 473 |
| 15 | 419 |
| 15.05 | 420 |
| 15.1 | 441 |
| 15.15 | 391 |
| 15.2 | 431 |
| 15.25 | 430 |
| 15.3 | 450 |
| 15.35 | 419 |
| 15.4 | 411 |
| 15.45 | 410 |
| 15.5 | 468 |
| 15.55 | 458 |
| 15.6 | 419 |
| 15.65 | 422 |
| 15.7 | 429 |
| 15.75 | 420 |
| 15.8 | 409 |
| 15.85 | 413 |
| 15.9 | 392 |
| 15.95 | 472 |
| 16 | 399 |
| 16.05 | 398 |
| 16.1 | 400 |
| 16.15 | 453 |
| 16.2 | 391 |
| 16.25 | 388 |
| 16.3 | 462 |
| 16.35 | 412 |
| 16.4 | 470 |
| 16.45 | 468 |
| 16.5 | 501 |
| 16.55 | 440 |
| 16.6 | 492 |
| 16.65 | 493 |
| 16.7 | 469 |
| 16.75 | 421 |
| 16.8 | 390 |
| 16.85 | 388 |
| 16.9 | 423 |
| 16.95 | 432 |
| 17 | 429 |
| 17.05 | 429 |
| 17.1 | 478 |
| 17.15 | 421 |
| 17.2 | 460 |
| 17.25 | 459 |
| 17.3 | 442 |
| 17.35 | 453 |
| 17.4 | 471 |
| 17.45 | 473 |
| 17.5 | 470 |
| 17.55 | 441 |
| 17.6 | 432 |
| 17.65 | 428 |
| 17.7 | 462 |
| 17.75 | 471 |
| 17.8 | 462 |
| 17.85 | 463 |
| 17.9 | 510 |
| 17.95 | 468 |
| 18 | 453 |
| 18.05 | 451 |
| 18.1 | 421 |
| 18.15 | 459 |
| 18.2 | 449 |
| 18.25 | 448 |
| 18.3 | 470 |
| 18.35 | 469 |
| 18.4 | 500 |
| 18.45 | 501 |
| 18.5 | 480 |
| 18.55 | 479 |
| 18.6 | 479 |
| 18.65 | 478 |
| 18.7 | 548 |
| 18.75 | 510 |
| 18.8 | 542 |
| 18.85 | 541 |
| 18.9 | 530 |
| 18.95 | 522 |
| 19 | 530 |
| 19.05 | 530 |
| 19.1 | 461 |
| 19.15 | 553 |
| 19.2 | 460 |
| 19.25 | 462 |
| 19.3 | 451 |
| 19.35 | 559 |
| 19.4 | 528 |
| 19.45 | 529 |
| 19.5 | 530 |
| 19.55 | 533 |
| 19.6 | 549 |
| 19.65 | 550 |
| 19.7 | 552 |
| 19.75 | 489 |
| 19.8 | 573 |
| 19.85 | 571 |
| 19.9 | 551 |
| 19.95 | 548 |
| 20 | 558 |
| 20.05 | 562 |
| 20.1 | 560 |
| 20.15 | 539 |
| 20.2 | 509 |
| 20.25 | 509 |
| 20.3 | 599 |
| 20.35 | 510 |
| 20.4 | 600 |
| 20.45 | 602 |
| 20.5 | 542 |
| 20.55 | 561 |
| 20.6 | 561 |
| 20.65 | 562 |
| 20.7 | 540 |
| 20.75 | 479 |
| 20.8 | 573 |
| 20.85 | 573 |
| 20.9 | 581 |
| 20.95 | 572 |
| 21 | 553 |
| 21.05 | 548 |
| 21.1 | 563 |
| 21.15 | 532 |
| 21.2 | 543 |
| 21.25 | 542 |
| 21.3 | 538 |
| 21.35 | 541 |
| 21.4 | 579 |
| 21.45 | 582 |
| 21.5 | 612 |
| 21.55 | 633 |
| 21.6 | 589 |
| 21.65 | 589 |
| 21.7 | 523 |
| 21.75 | 540 |
| 21.8 | 561 |
| 21.85 | 562 |
| 21.9 | 563 |
| 21.95 | 570 |
| 22 | 591 |
| 22.05 | 591 |
| 22.1 | 598 |
| 22.15 | 619 |
| 22.2 | 571 |
| 22.25 | 569 |
| 22.3 | 629 |
| 22.35 | 568 |
| 22.4 | 601 |
| 22.45 | 601 |
| 22.5 | 590 |
| 22.55 | 558 |
| 22.6 | 553 |
| 22.65 | 552 |
| 22.7 | 591 |
| 22.75 | 559 |
| 22.8 | 539 |
| 22.85 | 540 |
| 22.9 | 581 |
| 22.95 | 518 |
| 23 | 520 |
| 23.05 | 619 |
| 23.1 | 613 |
| 23.15 | 629 |
| 23.2 | 628 |
| 23.25 | 582 |
| 23.3 | 563 |
| 23.35 | 600 |
| 23.4 | 599 |
| 23.45 | 538 |
| 23.5 | 558 |
| 23.55 | 559 |
| 23.6 | 559 |
| 23.65 | 509 |
| 23.7 | 522 |
| 23.75 | 491 |
| 23.8 | 489 |
| 23.85 | 499 |
| 23.9 | 469 |
| 23.95 | 492 |
| 24 | 488 |
| 24.05 | 471 |
| 24.1 | 493 |
| 24.15 | 532 |
| 24.2 | 531 |
| 24.25 | 502 |
| 24.3 | 530 |
| 24.35 | 511 |
| 24.4 | 512 |
| 24.45 | 448 |
| 24.5 | 440 |
| 24.55 | 442 |
| 24.6 | 440 |
| 24.65 | 471 |
| 24.7 | 449 |
| 24.75 | 488 |
| 24.8 | 492 |
| 24.85 | 511 |
| 24.9 | 508 |
| 24.95 | 419 |
| 25 | 419 |
| 25.05 | 468 |
| 25.1 | 549 |
| 25.15 | 441 |
| 25.2 | 442 |
| 25.25 | 471 |
| 25.3 | 420 |
| 25.35 | 488 |
| 25.4 | 490 |
| 25.45 | 428 |
| 25.5 | 432 |
| 25.55 | 498 |
| 25.6 | 498 |
| 25.65 | 443 |
| 25.7 | 411 |
| 25.75 | 501 |
| 25.8 | 502 |
| 25.85 | 473 |
| 25.9 | 408 |
| 25.95 | 450 |
| 26 | 449 |
| 26.05 | 459 |
| 26.1 | 430 |
| 26.15 | 420 |
| 26.2 | 422 |
| 26.25 | 459 |
| 26.3 | 442 |
| 26.35 | 410 |
| 26.4 | 411 |
| 26.45 | 449 |
| 26.5 | 411 |
| 26.55 | 423 |
| 26.6 | 419 |
| 26.65 | 430 |
| 26.7 | 499 |
| 26.75 | 522 |
| 26.8 | 522 |
| 26.85 | 452 |
| 26.9 | 408 |
| 26.95 | 418 |
| 27 | 419 |
| 27.05 | 422 |
| 27.1 | 429 |
| 27.15 | 461 |
| 27.2 | 461 |
| 27.25 | 420 |
| 27.3 | 403 |
| 27.35 | 379 |
| 27.4 | 379 |
| 27.45 | 409 |
| 27.5 | 400 |
| 27.55 | 362 |
| 27.6 | 358 |
| 27.65 | 393 |
| 27.7 | 412 |
| 27.75 | 409 |
| 27.8 | 411 |
| 27.85 | 420 |
| 27.9 | 412 |
| 27.95 | 398 |
| 28 | 401 |
| 28.05 | 392 |
| 28.1 | 408 |
| 28.15 | 392 |
| 28.2 | 392 |
| 28.25 | 430 |
| 28.3 | 392 |
| 28.35 | 360 |
| 28.4 | 361 |
| 28.45 | 472 |
| 28.5 | 402 |
| 28.55 | 369 |
| 28.6 | 372 |
| 28.65 | 390 |
| 28.7 | 370 |
| 28.75 | 402 |
| 28.8 | 400 |
| 28.85 | 383 |
| 28.9 | 379 |
| 28.95 | 369 |
| 29 | 372 |
| 29.05 | 372 |
| 29.1 | 360 |
| 29.15 | 368 |
| 29.2 | 373 |
| 29.25 | 410 |
| 29.3 | 399 |
| 29.35 | 458 |
| 29.4 | 461 |
| 29.45 | 480 |
| 29.5 | 529 |
| 29.55 | 562 |
| 29.6 | 561 |
| 29.65 | 541 |
| 29.7 | 529 |
| 29.75 | 508 |
| 29.8 | 510 |
| 29.85 | 459 |
| 29.9 | 479 |
| 29.95 | 509 |
| 30 | 509 |
| 30.05 | 443 |
| 30.1 | 483 |
| 30.15 | 383 |
| 30.2 | 379 |
| 30.25 | 372 |
| 30.3 | 369 |
| 30.35 | 352 |
| 30.4 | 351 |
| 30.45 | 359 |
| 30.5 | 360 |
| 30.55 | 370 |
| 30.6 | 370 |
| 30.65 | 382 |
| 30.7 | 361 |
| 30.75 | 350 |
| 30.8 | 351 |
| 30.85 | 331 |
| 30.9 | 352 |
| 30.95 | 350 |
| 31 | 349 |
| 31.05 | 399 |
| 31.1 | 342 |
| 31.15 | 339 |
| 31.2 | 339 |
| 31.25 | 350 |
| 31.3 | 370 |
| 31.35 | 321 |
| 31.4 | 319 |
| 31.45 | 350 |
| 31.5 | 328 |
| 31.55 | 333 |
| 31.6 | 370 |
| 31.65 | 371 |
| 31.7 | 382 |
| 31.75 | 379 |
| 31.8 | 380 |
| 31.85 | 382 |
| 31.9 | 312 |
| 31.95 | 309 |
| 32 | 339 |
| 32.05 | 312 |
| 32.1 | 300 |
| 32.15 | 300 |
| 32.2 | 321 |
| 32.25 | 282 |
| 32.3 | 321 |
| 32.35 | 322 |
| 32.4 | 299 |
| 32.45 | 320 |
| 32.5 | 311 |
| 32.55 | 313 |
| 32.6 | 343 |
| 32.65 | 310 |
| 32.7 | 341 |
| 32.75 | 339 |
| 32.8 | 328 |
| 32.85 | 359 |
| 32.9 | 300 |
| 32.95 | 302 |
| 33 | 330 |
| 33.05 | 369 |
| 33.1 | 379 |
| 33.15 | 382 |
| 33.2 | 429 |
| 33.25 | 363 |
| 33.3 | 371 |
| 33.35 | 373 |
| 33.4 | 403 |
| 33.45 | 361 |
| 33.5 | 400 |
| 33.55 | 399 |
| 33.6 | 353 |
| 33.65 | 329 |
| 33.7 | 342 |
| 33.75 | 338 |
| 33.8 | 400 |
| 33.85 | 341 |
| 33.9 | 320 |
| 33.95 | 319 |
| 34 | 350 |
| 34.05 | 293 |
| 34.1 | 358 |
| 34.15 | 362 |
| 34.2 | 369 |
| 34.25 | 291 |
| 34.3 | 341 |
| 34.35 | 343 |
| 34.4 | 311 |
| 34.45 | 358 |
| 34.5 | 341 |
| 34.55 | 338 |
| 34.6 | 361 |
| 34.65 | 339 |
| 34.7 | 369 |
| 34.75 | 371 |
| 34.8 | 392 |
| 34.85 | 333 |
| 34.9 | 352 |
| 34.95 | 349 |
| 35 | 382 |
| 35.05 | 340 |
| 35.1 | 348 |
| 35.15 | 352 |
| 35.2 | 313 |
| 35.25 | 300 |
| 35.3 | 361 |
| 35.35 | 360 |
| 35.4 | 369 |
| 35.45 | 308 |
| 35.5 | 332 |
| 35.55 | 332 |
| 35.6 | 351 |
| 35.65 | 320 |
| 35.7 | 340 |
| 35.75 | 339 |
| 35.8 | 330 |
| 35.85 | 379 |
| 35.9 | 351 |
| 35.95 | 352 |
| 36 | 380 |
| 36.05 | 380 |
| 36.1 | 363 |
| 36.15 | 362 |
| 36.2 | 412 |
| 36.25 | 348 |
| 36.3 | 402 |
| 36.35 | 400 |
| 36.4 | 362 |
| 36.45 | 371 |
| 36.5 | 393 |
| 36.55 | 388 |
| 36.6 | 340 |
| 36.65 | 339 |
| 36.7 | 351 |
| 36.75 | 350 |
| 36.8 | 338 |
| 36.85 | 311 |
| 36.9 | 342 |
| 36.95 | 339 |
| 37 | 328 |
| 37.05 | 322 |
| 37.1 | 300 |
| 37.15 | 301 |
| 37.2 | 331 |
| 37.25 | 358 |
| 37.3 | 362 |
| 37.35 | 358 |
| 37.4 | 399 |
| 37.45 | 298 |
| 37.5 | 329 |
| 37.55 | 333 |
| 37.6 | 310 |
| 37.65 | 322 |
| 37.7 | 321 |
| 37.75 | 322 |
| 37.8 | 329 |
| 37.85 | 328 |
| 37.9 | 369 |
| 37.95 | 370 |
| 38 | 309 |
| 38.05 | 332 |
| 38.1 | 281 |
| 38.15 | 279 |
| 38.2 | 309 |
| 38.25 | 330 |
| 38.3 | 320 |
| 38.35 | 319 |
| 38.4 | 342 |
| 38.45 | 321 |
| 38.5 | 340 |
| 38.55 | 339 |
| 38.6 | 342 |
| 38.65 | 361 |
| 38.7 | 370 |
| 38.75 | 369 |
| 38.8 | 331 |
| 38.85 | 343 |
| 38.9 | 321 |
| 38.95 | 319 |
| 39 | 379 |
| 39.05 | 300 |
| 39.1 | 328 |
| 39.15 | 333 |
| 39.2 | 352 |
| 39.25 | 283 |
| 39.3 | 291 |
| 39.35 | 291 |
| 39.4 | 322 |
| 39.45 | 372 |
| 39.5 | 361 |
| 39.55 | 358 |
| 39.6 | 341 |
| 39.65 | 370 |
| 39.7 | 369 |
| 39.75 | 371 |
| 39.8 | 372 |
| 39.85 | 388 |
| 39.9 | 342 |
| 39.95 | 338 |
| 40 | 361 |
| 40.05 | 340 |
| 40.1 | 338 |
| 40.15 | 328 |
| 40.2 | 340 |
| 40.25 | 309 |
| 40.3 | 311 |
| 40.35 | 382 |
| 40.4 | 311 |
| 40.45 | 340 |
| 40.5 | 342 |
| 40.55 | 328 |
| 40.6 | 283 |
| 40.65 | 278 |
| 40.7 | 282 |
| 40.75 | 288 |
| 40.8 | 362 |
| 40.85 | 320 |
| 40.9 | 323 |
| 40.95 | 298 |
| 41 | 322 |
| 41.05 | 309 |
| 41.1 | 312 |
| 41.15 | 353 |
| 41.2 | 342 |
| 41.25 | 361 |
| 41.3 | 363 |
| 41.35 | 360 |
| 41.4 | 359 |
| 41.45 | 340 |
| 41.5 | 342 |
| 41.55 | 341 |
| 41.6 | 299 |
| 41.65 | 309 |
| 41.7 | 312 |
| 41.75 | 331 |
| 41.8 | 291 |
| 41.85 | 309 |
| 41.9 | 312 |
| 41.95 | 319 |
| 42 | 351 |
| 42.05 | 311 |
| 42.1 | 309 |
| 42.15 | 338 |
| 42.2 | 300 |
| 42.25 | 332 |
| 42.3 | 329 |
| 42.35 | 331 |
| 42.4 | 351 |
| 42.45 | 341 |
| 42.5 | 343 |
| 42.55 | 321 |
| 42.6 | 323 |
| 42.65 | 309 |
| 42.7 | 309 |
| 42.75 | 300 |
| 42.8 | 340 |
| 42.85 | 280 |
| 42.9 | 279 |
| 42.95 | 289 |
| 43 | 289 |
| 43.05 | 293 |
| 43.1 | 291 |
| 43.15 | 299 |
| 43.2 | 291 |
| 43.25 | 290 |
| 43.3 | 289 |
| 43.35 | 322 |
| 43.4 | 280 |
| 43.45 | 332 |
| 43.5 | 330 |
| 43.55 | 292 |
| 43.6 | 269 |
| 43.65 | 313 |
| 43.7 | 309 |
| 43.75 | 262 |
| 43.8 | 313 |
| 43.85 | 290 |
| 43.9 | 291 |
| 43.95 | 289 |
| 44 | 310 |
| 44.05 | 290 |
| 44.1 | 292 |
| 44.15 | 290 |
| 44.2 | 291 |
| 44.25 | 298 |
| 44.3 | 299 |
| 44.35 | 280 |
| 44.4 | 262 |
| 44.45 | 299 |
| 44.5 | 302 |
| 44.55 | 261 |
| 44.6 | 229 |
| 44.65 | 250 |
| 44.7 | 253 |
| 44.75 | 280 |
| 44.8 | 243 |
| 44.85 | 309 |
| 44.9 | 311 |
| 44.95 | 263 |
| 45 | 262 |
| 45.05 | 240 |
| 45.1 | 239 |
| 45.15 | 299 |
| 45.2 | 281 |
| 45.25 | 289 |
| 45.3 | 289 |
| 45.35 | 301 |
| 45.4 | 280 |
| 45.45 | 260 |
| 45.5 | 263 |
| 45.55 | 242 |
| 45.6 | 289 |
| 45.65 | 270 |
| 45.7 | 269 |
| 45.75 | 249 |
| 45.8 | 278 |
| 45.85 | 260 |
| 45.9 | 262 |
| 45.95 | 302 |
| 46 | 242 |
| 46.05 | 248 |
| 46.1 | 251 |
| 46.15 | 268 |
| 46.2 | 252 |
| 46.25 | 241 |
| 46.3 | 241 |
| 46.35 | 280 |
| 46.4 | 250 |
| 46.45 | 281 |
| 46.5 | 281 |
| 46.55 | 280 |
| 46.6 | 232 |
| 46.65 | 219 |
| 46.7 | 220 |
| 46.75 | 240 |
| 46.8 | 241 |
| 46.85 | 221 |
| 46.9 | 220 |
| 46.95 | 253 |
| 47 | 238 |
| 47.05 | 261 |
| 47.1 | 259 |
| 47.15 | 242 |
| 47.2 | 242 |
| 47.25 | 263 |
| 47.3 | 262 |
| 47.35 | 231 |
| 47.4 | 260 |
| 47.45 | 241 |
| 47.5 | 240 |
| 47.55 | 310 |
| 47.6 | 283 |
| 47.65 | 259 |
| 47.7 | 260 |
| 47.75 | 281 |
| 47.8 | 262 |
| 47.85 | 269 |
| 47.9 | 271 |
| 47.95 | 292 |
| 48 | 223 |
| 48.05 | 242 |
| 48.1 | 239 |
| 48.15 | 271 |
| 48.2 | 251 |
| 48.25 | 253 |
| 48.3 | 250 |
| 48.35 | 239 |
| 48.4 | 259 |
| 48.45 | 231 |
| 48.5 | 233 |
| 48.55 | 243 |
| 48.6 | 261 |
| 48.65 | 262 |
| 48.7 | 260 |
| 48.75 | 240 |
| 48.8 | 283 |
| 48.85 | 279 |
| 48.9 | 248 |
| 48.95 | 230 |
| 49 | 261 |
| 49.05 | 261 |
| 49.1 | 220 |
| 49.15 | 241 |
| 49.2 | 249 |
| 49.25 | 249 |
| 49.3 | 271 |
| 49.35 | 222 |
| 49.4 | 230 |
| 49.45 | 230 |
| 49.5 | 251 |
| 49.55 | 230 |
| 49.6 | 228 |
| 49.65 | 228 |
| 49.7 | 212 |
| 49.75 | 190 |
| 49.8 | 229 |
| 49.85 | 230 |
| 49.9 | 199 |
| 49.95 | 222 |
| 50 | 189 |
| 50.05 | 188 |
| 50.1 | 223 |
| 50.15 | 231 |
| 50.2 | 202 |
| 50.25 | 198 |
| 50.3 | 200 |
| 50.35 | 219 |
| 50.4 | 212 |
| 50.45 | 209 |
| 50.5 | 221 |
| 50.55 | 169 |
| 50.6 | 219 |
| 50.65 | 220 |
| 50.7 | 189 |
| 50.75 | 200 |
| 50.8 | 231 |
| 50.85 | 231 |
| 50.9 | 180 |
| 50.95 | 223 |
| 51 | 182 |
| 51.05 | 180 |
| 51.1 | 180 |
| 51.15 | 208 |
| 51.2 | 202 |
| 51.25 | 200 |
| 51.3 | 161 |
| 51.35 | 210 |
| 51.4 | 200 |
| 51.45 | 198 |
| 51.5 | 169 |
| 51.55 | 202 |
| 51.6 | 183 |
| 51.65 | 180 |
| 51.7 | 180 |
| 51.75 | 220 |
| 51.8 | 189 |
| 51.85 | 188 |
| 51.9 | 198 |
| 51.95 | 189 |
| 52 | 192 |
| 52.05 | 189 |
| 52.1 | 183 |
| 52.15 | 212 |
| 52.2 | 192 |
| 52.25 | 188 |
| 52.3 | 181 |
| 52.35 | 179 |
| 52.4 | 192 |
| 52.45 | 188 |
| 52.5 | 159 |
| 52.55 | 182 |
| 52.6 | 198 |
| 52.65 | 201 |
| 52.7 | 202 |
| 52.75 | 209 |
| 52.8 | 179 |
| 52.85 | 180 |
| 52.9 | 208 |
| 52.95 | 188 |
| 53 | 201 |
| 53.05 | 199 |
| 53.1 | 202 |
| 53.15 | 193 |
| 53.2 | 189 |
| 53.25 | 188 |
| 53.3 | 150 |
| 53.35 | 180 |
| 53.4 | 200 |
| 53.45 | 199 |
| 53.5 | 158 |
| 53.55 | 181 |
| 53.6 | 150 |
| 53.65 | 153 |
| 53.7 | 171 |
| 53.75 | 143 |
| 53.8 | 150 |
| 53.85 | 150 |
| 53.9 | 172 |
| 53.95 | 180 |
| 54 | 209 |
| 54.05 | 210 |
| 54.1 | 159 |
| 54.15 | 189 |
| 54.2 | 169 |
| 54.25 | 172 |
| 54.3 | 169 |
| 54.35 | 179 |
| 54.4 | 170 |
| 54.45 | 169 |
| 54.5 | 169 |
| 54.55 | 153 |
| 54.6 | 142 |
| 54.65 | 140 |
| 54.7 | 170 |
| 54.75 | 149 |
| 54.8 | 188 |
| 54.85 | 188 |
| 54.9 | 159 |
| 54.95 | 150 |
| 55 | 151 |
| 55.05 | 153 |
| 55.1 | 169 |
| 55.15 | 190 |
| 55.2 | 178 |
| 55.25 | 181 |
| 55.3 | 162 |
| 55.35 | 181 |
| 55.4 | 182 |
| 55.45 | 181 |
| 55.5 | 159 |
| 55.55 | 150 |
| 55.6 | 168 |
| 55.65 | 172 |
| 55.7 | 158 |
| 55.75 | 178 |
| 55.8 | 160 |
| 55.85 | 161 |
| 55.9 | 150 |
| 55.95 | 172 |
| 56 | 162 |
| 56.05 | 161 |
| 56.1 | 162 |
| 56.15 | 150 |
| 56.2 | 179 |
| 56.25 | 179 |
| 56.3 | 172 |
| 56.35 | 151 |
| 56.4 | 140 |
| 56.45 | 140 |
| 56.5 | 148 |
| 56.55 | 159 |
| 56.6 | 132 |
| 56.65 | 131 |
| 56.7 | 182 |
| 56.75 | 179 |
| 56.8 | 171 |
| 56.85 | 168 |
| 56.9 | 172 |
| 56.95 | 131 |
| 57 | 193 |
| 57.05 | 190 |
| 57.1 | 168 |
| 57.15 | 151 |
| 57.2 | 162 |
| 57.25 | 161 |
| 57.3 | 180 |
| 57.35 | 141 |
| 57.4 | 140 |
| 57.45 | 172 |
| 57.5 | 149 |
| 57.55 | 149 |
| 57.6 | 153 |
| 57.65 | 160 |
| 57.7 | 131 |
| 57.75 | 180 |
| 57.8 | 180 |
| 57.85 | 189 |
| 57.9 | 170 |
| 57.95 | 160 |
| 58 | 160 |
| 58.05 | 182 |
| 58.1 | 169 |
| 58.15 | 179 |
| 58.2 | 180 |
| 58.25 | 152 |
| 58.3 | 148 |
| 58.35 | 180 |
| 58.4 | 180 |
| 58.45 | 133 |
| 58.5 | 140 |
| 58.55 | 150 |
| 58.6 | 152 |
| 58.65 | 180 |
| 58.7 | 129 |
| 58.75 | 152 |
| 58.8 | 149 |
| 58.85 | 150 |
| 58.9 | 142 |
| 58.95 | 149 |
| 59 | 152 |
| 59.05 | 172 |
| 59.1 | 151 |
| 59.15 | 122 |
| 59.2 | 120 |
| 59.25 | 183 |
| 59.3 | 148 |
| 59.35 | 142 |
| 59.4 | 142 |
| 59.45 | 139 |
| 59.5 | 172 |
| 59.55 | 142 |
| 59.6 | 138 |
| 59.65 | 130 |
| 59.7 | 162 |
| 59.75 | 148 |
| 59.8 | 148 |
| 59.85 | 169 |
| 59.9 | 161 |
| 59.95 | 128 |
| 60 | 129 |
| 60.05 | 181 |
| 60.1 | 180 |
| 60.15 | 150 |
| 60.2 | 149 |
| 60.25 | 151 |
| 60.3 | 152 |
| 60.35 | 149 |
| 60.4 | 151 |
| 60.45 | 149 |
| 60.5 | 169 |
| 60.55 | 142 |
| 60.6 | 139 |
| 60.65 | 131 |
| 60.7 | 159 |
| 60.75 | 170 |
| 60.8 | 173 |
| 60.85 | 152 |
| 60.9 | 142 |
| 60.95 | 193 |
| 61 | 189 |
| 61.05 | 160 |
| 61.1 | 119 |
| 61.15 | 171 |
| 61.2 | 172 |
| 61.25 | 133 |
| 61.3 | 159 |
| 61.35 | 162 |
| 61.4 | 160 |
| 61.45 | 139 |
| 61.5 | 159 |
| 61.55 | 141 |
| 61.6 | 141 |
| 61.65 | 139 |
| 61.7 | 172 |
| 61.75 | 149 |
| 61.8 | 148 |
| 61.85 | 153 |
| 61.9 | 159 |
| 61.95 | 131 |
| 62 | 132 |
| 62.05 | 130 |
| 62.1 | 168 |
| 62.15 | 150 |
| 62.2 | 149 |
| 62.25 | 162 |
| 62.3 | 121 |
| 62.35 | 159 |
| 62.4 | 160 |
| 62.45 | 110 |
| 62.5 | 162 |
| 62.55 | 150 |
| 62.6 | 152 |
| 62.65 | 138 |
| 62.7 | 170 |
| 62.75 | 130 |
| 62.8 | 131 |
| 62.85 | 163 |
| 62.9 | 143 |
| 62.95 | 133 |
| 63 | 131 |
| 63.05 | 141 |
| 63.1 | 159 |
| 63.15 | 149 |
| 63.2 | 152 |
| 63.25 | 153 |
| 63.3 | 129 |
| 63.35 | 163 |
| 63.4 | 161 |
| 63.45 | 112 |
| 63.5 | 128 |
| 63.55 | 130 |
| 63.6 | 132 |
| 63.65 | 132 |
| 63.7 | 160 |
| 63.75 | 150 |
| 63.8 | 153 |
| 63.85 | 123 |
| 63.9 | 103 |
| 63.95 | 180 |
| 64 | 180 |
| 64.05 | 120 |
| 64.1 | 139 |
| 64.15 | 121 |
| 64.2 | 121 |
| 64.25 | 160 |
| 64.3 | 148 |
| 64.35 | 130 |
| 64.4 | 131 |
| 64.45 | 131 |
| 64.5 | 138 |
| 64.55 | 130 |
| 64.6 | 130 |
| 64.65 | 141 |
| 64.7 | 160 |
| 64.75 | 129 |
| 64.8 | 130 |
| 64.85 | 132 |
| 64.9 | 162 |
| 64.95 | 163 |
| 65 | 160 |
| 65.05 | 142 |
| 65.1 | 130 |
| 65.15 | 140 |
| 65.2 | 143 |
| 65.25 | 108 |
| 65.3 | 119 |
| 65.35 | 132 |
| 65.4 | 133 |
| 65.45 | 131 |
| 65.5 | 129 |
| 65.55 | 109 |
| 65.6 | 113 |
| 65.65 | 108 |
| 65.7 | 171 |
| 65.75 | 108 |
| 65.8 | 112 |
| 65.85 | 152 |
| 65.9 | 140 |
| 65.95 | 142 |
| 66 | 132 |
| 66.05 | 142 |
| 66.1 | 142 |
| 66.15 | 143 |
| 66.2 | 101 |
| 66.25 | 159 |
| 66.3 | 129 |
| 66.35 | 129 |
| 66.4 | 122 |
| 66.45 | 129 |
| 66.5 | 110 |
| 66.55 | 112 |
| 66.6 | 133 |
| 66.65 | 120 |
| 66.7 | 133 |
| 66.75 | 129 |
| 66.8 | 130 |
| 66.85 | 161 |
| 66.9 | 109 |
| 66.95 | 108 |
| 67 | 150 |
| 67.05 | 152 |
| 67.1 | 142 |
| 67.15 | 141 |
| 67.2 | 108 |
| 67.25 | 119 |
| 67.3 | 99 |
| 67.35 | 103 |
| 67.4 | 131 |
| 67.45 | 172 |
| 67.5 | 112 |
| 67.55 | 110 |
| 67.6 | 121 |
| 67.65 | 132 |
| 67.7 | 123 |
| 67.75 | 118 |
| 67.8 | 139 |
| 67.85 | 109 |
| 67.9 | 132 |
| 67.95 | 128 |
| 68 | 109 |
| 68.05 | 149 |
| 68.1 | 118 |
| 68.15 | 123 |
| 68.2 | 142 |
| 68.25 | 121 |
| 68.3 | 99 |
| 68.35 | 101 |
| 68.4 | 130 |
| 68.45 | 152 |
| 68.5 | 149 |
| 68.55 | 150 |
| 68.6 | 149 |
| 68.65 | 138 |
| 68.7 | 120 |
| 68.75 | 118 |
| 68.8 | 122 |
| 68.85 | 120 |
| 68.9 | 139 |
| 68.95 | 142 |
| 69 | 132 |
| 69.05 | 150 |
| 69.1 | 143 |
| 69.15 | 140 |
| 69.2 | 113 |
| 69.25 | 110 |
| 69.3 | 109 |
| 69.35 | 112 |
| 69.4 | 150 |
| 69.45 | 152 |
| 69.5 | 139 |
| 69.55 | 142 |
| 69.6 | 139 |
| 69.65 | 152 |
| 69.7 | 122 |
| 69.75 | 119 |
| 69.8 | 131 |
| 69.85 | 93 |
| 69.9 | 132 |
| 69.95 | 128 |
| 70 | 152 |
| 70.05 | 171 |
| 70.1 | 128 |
| 70.15 | 130 |
| 70.2 | 142 |
| 70.25 | 170 |
| 70.3 | 130 |
| 70.35 | 133 |
| 70.4 | 119 |
| 70.45 | 119 |
| 70.5 | 152 |
| 70.55 | 150 |
| 70.6 | 120 |
| 70.65 | 108 |
| 70.7 | 128 |
| 70.75 | 128 |
| 70.8 | 112 |
| 70.85 | 131 |
| 70.9 | 109 |
| 70.95 | 112 |
| 71 | 120 |
| 71.05 | 122 |
| 71.1 | 139 |
| 71.15 | 141 |
| 71.2 | 111 |
| 71.25 | 140 |
| 71.3 | 113 |
| 71.35 | 112 |
| 71.4 | 152 |
| 71.45 | 123 |
| 71.5 | 128 |
| 71.55 | 131 |
| 71.6 | 141 |
| 71.65 | 99 |
| 71.7 | 129 |
| 71.75 | 131 |
| 71.8 | 99 |
| 71.85 | 122 |
| 71.9 | 132 |
| 71.95 | 130 |
| 72 | 162 |
| 72.05 | 132 |
| 72.1 | 112 |
| 72.15 | 112 |
| 72.2 | 109 |
| 72.25 | 131 |
| 72.3 | 119 |
| 72.35 | 119 |
| 72.4 | 163 |
| 72.45 | 131 |
| 72.5 | 102 |
| 72.55 | 103 |
| 72.6 | 139 |
| 72.65 | 129 |
| 72.7 | 132 |
| 72.75 | 133 |
| 72.8 | 133 |
| 72.85 | 129 |
| 72.9 | 142 |
| 72.95 | 138 |
| 73 | 151 |
| 73.05 | 109 |
| 73.1 | 139 |
| 73.15 | 139 |
| 73.2 | 122 |
| 73.25 | 121 |
| 73.3 | 140 |
| 73.35 | 139 |
| 73.4 | 138 |
| 73.45 | 148 |
| 73.5 | 162 |
| 73.55 | 159 |
| 73.6 | 112 |
| 73.65 | 118 |
| 73.7 | 140 |
| 73.75 | 138 |
| 73.8 | 131 |
| 73.85 | 129 |
| 73.9 | 150 |
| 73.95 | 152 |
| 74 | 131 |
| 74.05 | 112 |
| 74.1 | 122 |
| 74.15 | 120 |
| 74.2 | 102 |
| 74.25 | 98 |
| 74.3 | 150 |
| 74.35 | 149 |
| 74.4 | 121 |
| 74.45 | 139 |
| 74.5 | 141 |
| 74.55 | 140 |
| 74.6 | 121 |
| 74.65 | 128 |
| 74.7 | 129 |
| 74.75 | 111 |
| 74.8 | 129 |
| 74.85 | 99 |
| 74.9 | 99 |
| 74.95 | 121 |
| 75 | 140 |
| 75.05 | 160 |
| 75.1 | 161 |
| 75.15 | 132 |
| 75.2 | 99 |
| 75.25 | 141 |
| 75.3 | 141 |
| 75.35 | 121 |
| 75.4 | 79 |
| 75.45 | 120 |
| 75.5 | 121 |
| 75.55 | 160 |
| 75.6 | 132 |
| 75.65 | 119 |
| 75.7 | 119 |
| 75.75 | 122 |
| 75.8 | 100 |
| 75.85 | 130 |
| 75.9 | 128 |
| 75.95 | 111 |
| 76 | 142 |
| 76.05 | 99 |
| 76.1 | 101 |
| 76.15 | 140 |
| 76.2 | 149 |
| 76.25 | 121 |
| 76.3 | 121 |
| 76.35 | 130 |
| 76.4 | 121 |
| 76.45 | 109 |
| 76.5 | 110 |
| 76.55 | 109 |
| 76.6 | 129 |
| 76.65 | 142 |
| 76.7 | 140 |
| 76.75 | 119 |
| 76.8 | 120 |
| 76.85 | 128 |
| 76.9 | 131 |
| 76.95 | 150 |
| 77 | 142 |
| 77.05 | 122 |
| 77.1 | 119 |
| 77.15 | 122 |
| 77.2 | 142 |
| 77.25 | 141 |
| 77.3 | 141 |
| 77.35 | 102 |
| 77.4 | 122 |
| 77.45 | 112 |
| 77.5 | 110 |
| 77.55 | 120 |
| 77.6 | 112 |
| 77.65 | 109 |
| 77.7 | 111 |
| 77.75 | 110 |
| 77.8 | 110 |
| 77.85 | 110 |
| 77.9 | 111 |
| 77.95 | 118 |
| 78 | 132 |
| 78.05 | 121 |
| 78.1 | 120 |
| 78.15 | 128 |
| 78.2 | 112 |
| 78.25 | 110 |
| 78.3 | 111 |
| 78.35 | 130 |
| 78.4 | 118 |
| 78.45 | 120 |
| 78.5 | 123 |
| 78.55 | 112 |
| 78.6 | 109 |
| 78.65 | 120 |
| 78.7 | 121 |
| 78.75 | 90 |
| 78.8 | 109 |
| 78.85 | 102 |
| 78.9 | 98 |
| 78.95 | 111 |
| 79 | 133 |
| 79.05 | 113 |
| 79.1 | 111 |
| 79.15 | 122 |
| 79.2 | 119 |
| 79.25 | 90 |
| 79.3 | 90 |
| 79.35 | 122 |
| 79.4 | 132 |
| 79.45 | 111 |
| 79.5 | 110 |
| 79.55 | 140 |
| 79.6 | 150 |
| 79.65 | 109 |
| 79.7 | 111 |
| 79.75 | 98 |
| 79.8 | 108 |
| 79.85 | 120 |
| 79.9 | 118 |
| 79.95 | 140 |
| 80 | 181 |

Source for Fig. 3


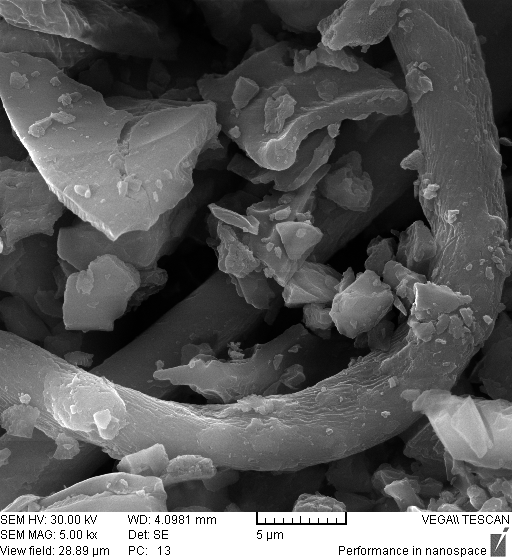


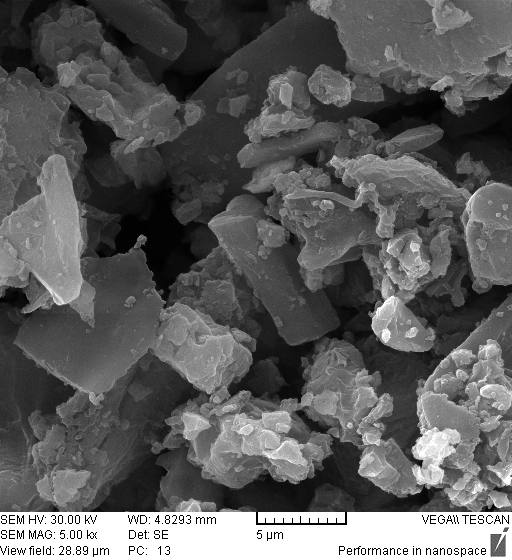


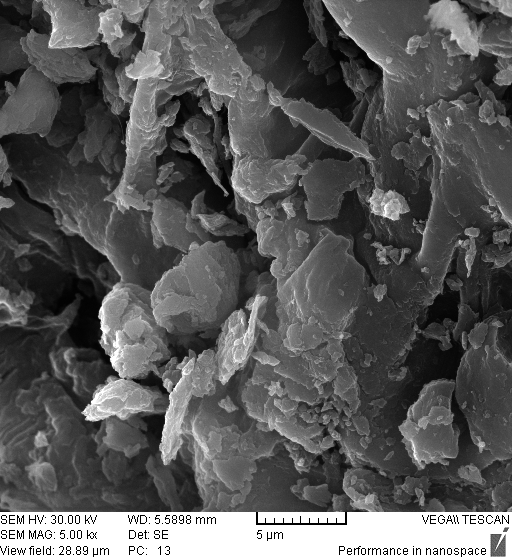


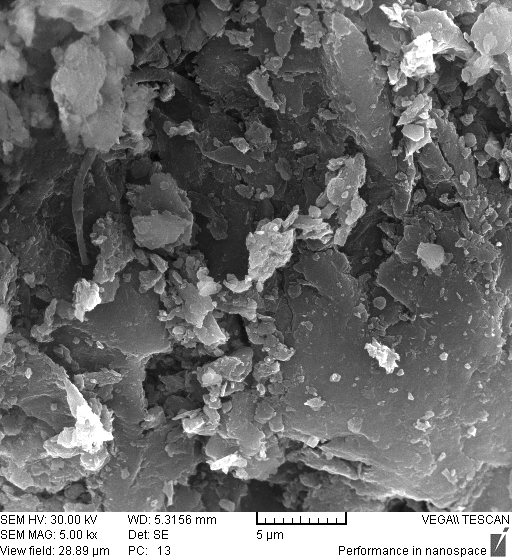


Source Fig. 4.

| pH |  |  |  |  |
| --- | --- | --- | --- | --- |
|  | ZSAC | BSO | SD | SD |
| 2 | 32 | 30 | 0.32 | 0.3 |
| 3 | 42 | 40 | 0.42 | 0.4 |
| 4 | 55 | 51 | 0.55 | 0.51 |
| 5 | 68 | 62 | 0.68 | 0.62 |
| 6 | 80 | 70 | 0.8 | 0.7 |
| 7 | 88 | 80 | 0.88 | 0.8 |
| 8 | 92 | 89 | 0.92 | 0.89 |
| 9 | 89 | 84 | 0.89 | 0.84 |
| 10 | 87 | 80 | 0.87 | 0.8 |

Source Fig. 5.

| 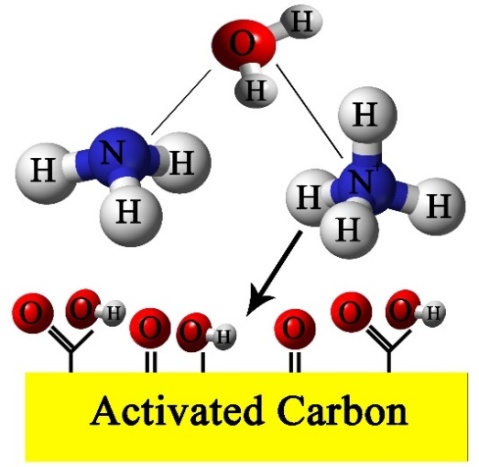  **(a)** | 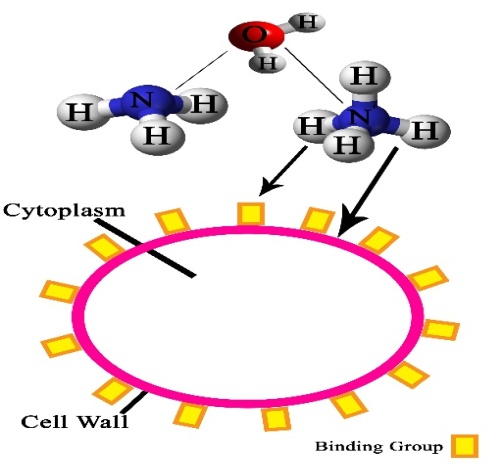  **(b)** |
| --- | --- |

Source Fig. 6.

| Time |  |  |  |  |
| --- | --- | --- | --- | --- |
|  | ZSAC | BSO | SD | SD |
| 10 | 62 | 55 | 0.62 | 0.55 |
| 20 | 70 | 64 | 0.7 | 0.64 |
| 30 | 76 | 72 | 0.76 | 0.72 |
| 40 | 83 | 80 | 0.83 | 0.8 |
| 50 | 88 | 85 | 0.88 | 0.85 |
| 60 | 92 | 89 | 0.92 | 0.89 |
| 70 | 94.2 | 92 | 0.942 | 0.92 |
| 80 | 95.6 | 94.5 | 0.956 | 0.945 |
| 90 | 95.6 | 94.6 | 0.956 | 0.946 |
| 100 | 95.6 | 94.6 | 0.956 | 0.946 |

Source Fig. 7.

| Dose |  |  |  |  |
| --- | --- | --- | --- | --- |
|  | ZSAC | BSO | SD | SD |
| 1 | 88.2 | 86 | 0.882 | 0.86 |
| 2 | 95.6 | 94.5 | 0.956 | 0.945 |
| 3 | 97.1 | 95.8 | 0.971 | 0.958 |
| 5 | 97.9 | 96.2 | 0.979 | 0.962 |
| 7 | 97.9 | 96.3 | 0.979 | 0.963 |
| 10 | 97.9 | 96.3 | 0.979 | 0.963 |

Source Fig. 8.

| Temp. |  |  |  |  |
| --- | --- | --- | --- | --- |
|  | ZSAC | BSO | SD | SD |
| 25 | 97.9 | 96.2 | 0.979 | 0.962 |
| 30 | 95.1 | 93.1 | 0.951 | 0.931 |
| 40 | 90.2 | 88.2 | 0.902 | 0.882 |
| 50 | 83.5 | 80 | 0.835 | 0.8 |
| 60 | 75.4 | 71.3 | 0.754 | 0.713 |

Source Table 3.

| Isotherm | Parameters | ZSAC | BSO |
| --- | --- | --- | --- |
| Langmuir  ZSAC: y = 0.0388x + 0.4255  BSO: y = 0.134x + 0.2728 | q_max_ (mg/g) | 25.77 | 7.46 |
|  | b (L/mg) | 0.09 | 0.49 |
|  | R^2^ | 0.991 | 0.987 |
| Freundlich  ZSAC: y = 2.3362x + 0.8589  BSO: y = 1.7159x + 0.6606 | K_f_ | 7.23 | 4.58 |
|  | n | 2.33 | 1.72 |
|  | R^2^ | 0.998 | 0.991 |

Source for Table 4.

| Reference | q_max_ (mg/g) | Bio-adsorbent |
| --- | --- | --- |
| [4] | 17.19 | Activated carbon |
| [5] | 1.73 | *Posidonia oceanica* fibers |
| [6] | 0.255 | Light expanded clay aggregate (LECA) |
| [7] | 9.31 | Multi-walled carbon nanotubes |
| [8] | 5.38 | Pin sawdust and wheat straw biochars |
| [9] | 19 | Volcanic tuff |
| [10] | 19.34 and 4.50 | Organic acid modified activated carbon and activated carbon |
| Present work | 25.77 | ZSAC |
| Present work | 7.46 | BSO |

Source for Fig. 9 & Table 5.

| ZSAC |  |  |  |  |
| --- | --- | --- | --- | --- |
| 1/T | T | kd | Delta G | Lnkd |
| 0.003356 | 298 | 9.32381 | -5.53269 | 2.232571 |
| 0.0033 | 303 | 3.881633 | -3.41742 | 1.356256 |
| 0.003195 | 313 | 1.840816 | -1.58832 | 0.610209 |
| 0.003096 | 323 | 1.012121 | -0.03236 | 0.012048 |
| 0.003003 | 333 | 0.613008 | 1.355197 | -0.48938 |

| BSO |  |  |  |  |
| --- | --- | --- | --- | --- |
| 1/T | T | kd | Delta G | Lnkd |
| 0.003356 | 298 | 5.063158 | -12.5474 | 1.62199 |
| 0.0033 | 303 | 2.698551 | -6.79967 | 0.992715 |
| 0.003195 | 313 | 1.494915 | -3.89113 | 0.40207 |
| 0.003096 | 323 | 0.8 | -2.14885 | -0.22314 |
| 0.003003 | 333 | 0.496864 | -1.37593 | -0.69944 |
